# Rapid evolution driven by translocation-associated selection during meiosis

**DOI:** 10.1038/s44319-026-00820-6

**Published:** 2026-06-16

**Authors:** Xuming Zeng, Mengdong Zhang, Xuanxuan Liu, Xionglei He, Li Liu

**Affiliations:** 1 State Key Laboratory of Biocontrol, MOE Key Laboratory of Gene Function and Regulation, School of Life Sciences, Sun Yat-Sen University, Guangzhou, 510275 China; 2 Innovation Center for Evolutionary Synthetic Biology, Sun Yat-Sen University, Guangzhou, 510275 China

**Keywords:** Cell Cycle, Evolution & Ecology

## Abstract

The evolution of allele frequencies in a population is often ascribed to differential fitness among organisms carrying different alleles. However, selection during meiosis can substantially influence this process. Here, we studied the evolution of a hybrid yeast population over six meiotic generations and observed rapid allele frequency dynamics at many genomic loci. By tracking the whole population and analyzing single gametes we discovered that biased segregation pattern during meiosis can drive rapid evolutionary changes at translocation-linked loci. The inter-chromosomal translocation present in one parental strain creates quadrivalent structures that promote adjacent-1 segregation coupled with crossover formation over alternate segregation during meiosis. This ultimately increases the proportion of unbalanced gametes, and consequently alters allele frequencies in the population. These findings demonstrate that meiotic selection operates more broadly than previously recognized and constitutes a significant evolutionary force affecting population allele frequencies. Given the prevalence of inter-chromosomal translocations, biased segregation pattern may complement the established role of translocations in shaping evolutionary outcomes.

## Synopsis

Translocation-associated quadrivalent segregation is non-random during meiosis, with selection favoring crossover-associated adjacent-1 segregation over alternate segregation. This meiotic-level selection can rapidly reshape population allele frequencies and may be widespread across species.

- During meiosis in hybrids heterozygous for reciprocal translocations, adjacent-1 segregation occurred significantly more frequently than alternate segregation across all crosses.
- Crossover-associated adjacent-1 segregation was predominant, accounting for 76%–93% of adjacent-1 events.
- Segregation bias associated with inter-chromosomal translocations may be an important factor influencing allele frequency dynamics, with frequency shifts extending ~300 kb from translocation breakpoints on chromosomes VIII and XVI within a short timeframe.

## Abstract (web summary)

Translocation-associated quadrivalent segregation is non-random during meiosis, with selection favoring crossover-associated adjacent-1 segregation over alternate segregation. This meiotic-level selection can rapidly reshape population allele frequencies and may be widespread across species.

## Graphical abstract

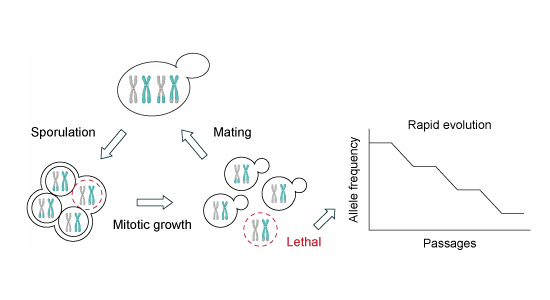

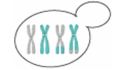

## Introduction

Natural populations are dynamic systems that evolve over time, with genetic material transmitted through generations following the fundamental principles of Mendelian inheritance (Dobzhansky, 1995). This evolutionary process shapes the genetic composition of populations and drives adaptation to changing environments. At the population level, evolutionary change manifests primarily through shifts in allele frequencies (Wright, 1931). This process is largely driven by the differential fitness advantages conferred by specific alleles to the organisms carrying them, ultimately influencing the genetic makeup of subsequent generations (Orr, 2009).

Central to genetic transmission is meiosis, a specialized cellular division process that produces haploid gametes such as eggs, sperm, and spores (Handel and Schimenti, 2010). The fundamental nature of meiosis is elegantly captured in Mendel’s Law of Segregation, which posits that parental alleles have an equal probability of transmission to offspring (Laird and Lange, 2011). However, increasing cases are discovered where the fairness of meiosis is disrupted (Wolf et al, 2022). Recent advances in genomic technologies have begun to reveal the molecular mechanisms underlying these phenomena, including meiotic drive systems where specific alleles manipulate the meiotic process to ensure their preferential transmission (Lindholm et al, 2016; Searle and Pardo-Manuel de Villena, 2024; Wolf et al, 2022).

This phenomenon becomes particularly relevant in systems with chromosomal rearrangements. For instance, Zanders et al reported a case in hybrid crosses between fission yeast species *Schizosaccharomyces pombe* and *Schizosaccharomyces kambucha*, where chromosomal rearrangements and related recombination defects serve as major causes of hybrid infertility, allowing meiosis to complete normally while producing predominantly inviable gametes (Zanders et al, 2014). Furthermore, numerous studies have demonstrated that multiple types of chromosomal rearrangements in humans induce high risks of producing unbalanced gametes during meiosis, leading to various reproductive challenges, including infertility, recurrent miscarriages, and genetic disorders in offspring (Kato et al, 2016; Simpson et al, 2019; Yu et al, 2023).

The effect of chromosomal rearrangements in producing unbalanced gametes with reduced viability has been extensively studied in the context of speciation and species isolation barriers (Charron et al, 2014; Delneri et al, 2003; Hou et al, 2014; Koszul et al, 2006; Leducq et al, 2016; Liti et al, 2006). From this perspective, chromosomal rearrangements act as physical barriers, especially in interspecies contexts, and represent important evolutionary forces. However, we sought to determine whether selection operates within meiotic processes in this context to influence gamete production. Traditionally, gametic selection in reproductive biology refers to differential gamete viability, motility advantages in sperm competition, and preferential fertilization success (Immler and Otto, 2018). Beyond these post-meiotic mechanisms, we reasoned that selection may act directly during the meiotic process, creating a biased segregation pattern that significantly deviates from Mendelian expectations. Delneri et al have reported offspring viability rates exceeding theoretical expectations in hybrids carrying heterozygous reciprocal translocations (RTs), raising the possibility that non-random segregation patterns may occur during meiosis (Delneri et al, 2003). This would introduce an additional layer of evolutionary pressure that operates at the chromosomal level during meiosis, ultimately affecting organismal fitness and population dynamics.

**Figure 1:**
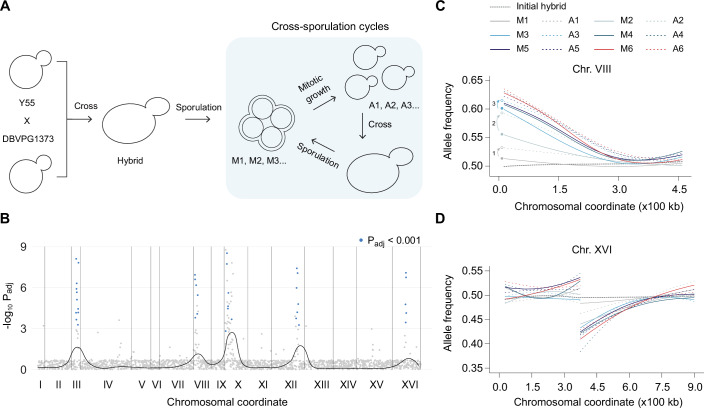
Significant deviations from Mendelian segregation ratios of allele frequencies across the genome. (**A**) Flowchart of successive cross-meiosis experiments in a yeast hybrid from the cross between Y55 and DBVPG1373. Mn represents the *n*th round of meiotic products, and An represents the nth round of mitotic growth products. Genomic DNA was extracted at both Mn and An time points for allele frequency determination by sequencing. (**B**) Five genomic regions, located on chromosomes III, VIII, X, XII, and XVI, exhibit significant deviations in allele frequency, with values falling below 0.4 or exceeding 0.6. The y-axis displays −log_10_ *P*_adj_ values from Kolmogorov–Smirnov tests, calculated using a sliding window approach to compare allele frequencies between sixth-round meiotic products and initial hybrids. Sites exceeding the statistical significance threshold are highlighted in blue, while the black curve represents the fitted line. (**C**, **D**) Allele frequency across chromosomes VIII and XVI changes from the initial hybrid diploid to the sixth round. The dotted line represents the initial hybrid, the solid lines represent the meiotic products collected after 7 days of sporulation (M1–M6), and the dashed lines are mitotic growth products collected after 16 h of growth in YPD medium (A1–A6). Curves were fitted from discrete SNP allele frequencies, and one SNP marker was selected every 200 bp for curve fitting for minimizing the effect of variable SNP density across regions. Values from the same round are shown in the same color. The cycle of sporulation (7 days) followed by mitotic growth (16 h) and mating (72 h) was repeated for six rounds. Source data are available online for this figure.

In this study, we conducted successive cross-meiosis experiments using a yeast hybrid population to trace allele frequency variations over time (Fig. 1A). The initial hybrid population was derived from a cross between two *Saccharomyces cerevisiae* (*S. cerevisiae*) strains: Y55 and DBVPG1373. Our analysis revealed rapid allele frequency dynamics at many genomic loci. Detailed genomic and tetrad analyses uncovered that at translocation-linked loci, frequency changes stemmed from biased segregation patterns in translocation-containing quadrivalents, where adjacent-1 segregation associated with crossovers was strongly favored over alternate segregation, yielding gametes with improved viability compared to classical adjacent-1 products but altered genotypic compositions. This segregation bias shifts the distribution of gametic genotypes, thereby driving changes in population allele frequencies. Our study reveals that meiotic selection through biased segregation patterns can contribute significantly to allele frequency dynamics in populations.

## Results

### Rapid allele frequency changes over time in a yeast hybrid population

**Figure EV1:**
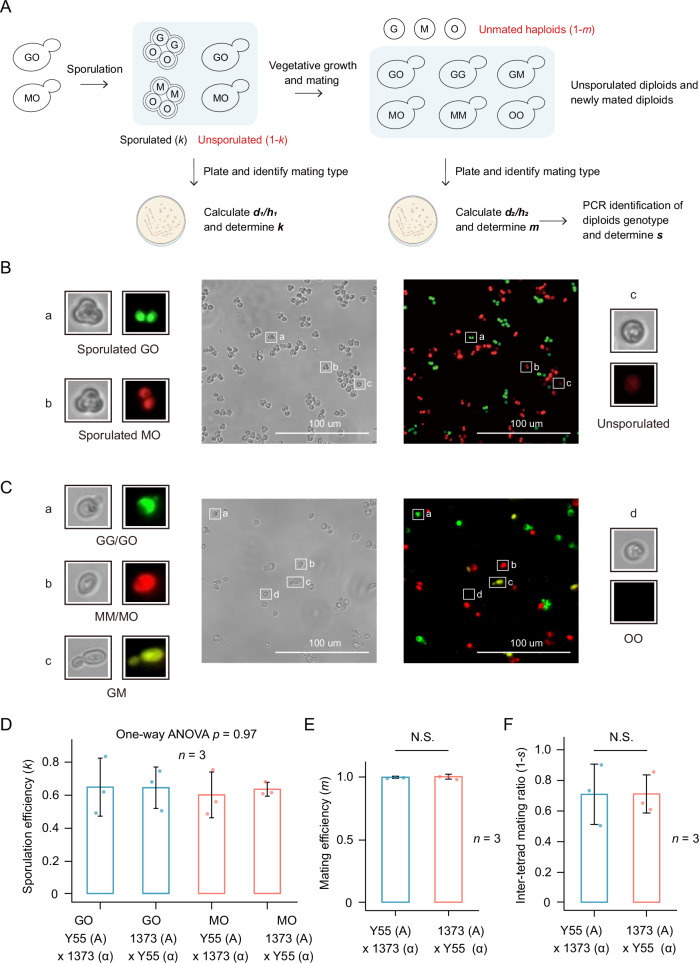
Quantitative analysis of intra- and inter- tetrad mating of spores. (**A**) Schematic illustration of the experiment. GO (GFP/-) and MO (mScarlet/-) strains were sporulated separately and then mixed at equal proportions in rich medium to allow vegetative growth and mating. Cell populations after sporulation and after mating were plated to determine the numbers of haploids and diploids, as well as the proportion of GM (GFP/mScarlet) cells, which were used to estimate the relative proportions of intra- and inter-tetrad mating. *k* sporulation rate, *m* mating efficiency, *s* proportion of intra-tetrad mating, *d1/h1* and *d2/h2* observed diploid-to-haploid ratios at the corresponding stages. Genotype: GG, GFP/GFP; MM, mScarlet/mScarlet; OO, −/−. (**B**) Representative microscopy images of cells after sporulation (before vegetative growth). Bright-field images are shown on the left, and the merged GFP and mScarlet fluorescence images are shown on the right. Representative cells marked by white boxes are magnified besides. (**C**) Representative microscopy images of cells after mating. Bright-field images are shown on the left, and merged GFP and mScarlet fluorescence images are shown on the right. Representative cells marked by white boxes are magnified besides. (**D**) Sporulation rates of the four different initial diploid genotypes. Data were shown as mean ± SD from three biological replicates per group (*n* = 3 biological replicates). No significant difference among groups was detected by one-way ANOVA (*p* = 0.97). (**E**) Mating efficiency of the reciprocal crosses. Data are shown as mean ± SD from three biological replicates per group (*n* = 3 biological replicates). No significant difference was detected by a two-sided Student’s *t*-test (*p* = 0.66). (**F**) Proportion of inter-tetrad mating of the reciprocal crosses. Data were shown as mean ± SD from three biological replicates per group (*n* = 3 biological replicates). No significant difference was detected by a two-sided Student’s *t*-test (*p* = 0.99).

We first performed a cross between Y55 (MATa) and DBVPG1373 (MATα). The hybrid has 65,830 heterozygous SNP markers distributed across the genome (Dataset EV1), which can be used to trace the genotypes of the products of cross and meiotic division (Methods). Three clones of hybrids were used for independent replicates. The hybrids were first manipulated to undergo meiosis, leading to the formation of haploid spores. The sporulation efficiency was 52.6–58.6% (Appendix Fig. S1). Then, the meiotic products from each group experienced a period of mitotic growth and were induced to cross again by changing the culture conditions subsequently. To determine whether mating during this step occurred predominantly within tetrads or between tetrads, we performed a fluorescent marker assay. We estimated that ~73% of newly formed diploids arose from inter-tetrad mating (Fig. EV1; Appendix Methods), indicating that inter-tetrad mating accounts for the majority of diploid re-formation events under our experimental conditions. The products of the secondary round of cross were then subjected to the next round of meiosis. This procedure was repeated for a total of six rounds (Fig. 1A). Genomic DNA of products from each round of meiotic division and mitotic growth were extracted and sequenced using whole-genome sequencing (WGS) (Methods). The allele frequency variations across each chromosome were monitored by the analysis of parental allele frequency and sequencing coverage of strains in each round.

In the initial hybrids, allele frequencies of both parental strains were equally distributed at 0.5 across all chromosomes (Appendix Fig. S2). We used DBVPG1373 allele frequencies as the reference for subsequent analyses. To identify regions with significant allele frequency changes following cross-meiosis experiments, we compared allele frequencies between sixth-round meiotic products and initial hybrids using a sliding window approach (Methods). Five genomic regions, located on chromosomes III, VIII, X, XII, and XVI, exhibited significant deviations in allele frequency, with values falling below 0.4 or exceeding 0.6 (*P*_adj_ < 0.001; Fig. 1B). To understand the progression of these variations, we examined allele frequencies in these regions across all rounds of meiotic division and mitotic growth (Dataset EV2). These regions showed increasingly pronounced deviations from 0.5 as the experiment progressed (Fig. 1C,D; Appendix Fig. S3). For instance, a ~300 kb region on the upstream end of chromosome VIII demonstrated preferential inheritance of the DBVPG1373 allele, with allele frequency rapidly rising to ~0.65, particularly during the first three rounds (Fig. 1C; Appendix Fig. S4). In contrast, a ~300 kb region on chromosome XVI exhibited preferential inheritance of the Y55 allele (Fig. 1D; Appendix Fig. S4).

This raised the question of whether these rapid allele frequency changes were driven by fitness advantages during mitotic growth or by differential spore viability during meiosis. Notably, the allele frequencies of meiotic products differed from those observed in mitotic growth products within the same round, but consistently matched the allele frequencies of the mitotic growth products from the preceding round, a pattern particularly evident on chromosome VIII (Fig. 1C). This pattern suggests that all spores from the previous round participated fully in the subsequent cross-meiosis events. Given the short duration of the mitotic growth phase (fewer than eight divisions), it is unlikely that strong selection for fitness advantages occurred during this period. We therefore propose that while the meiotic process produced four spores, differential spore viability and biased genomic composition may have driven the observed rapid evolution of population allele frequencies.

### Hybrid meiosis produces spores with biased allele inheritance

**Figure 2:**
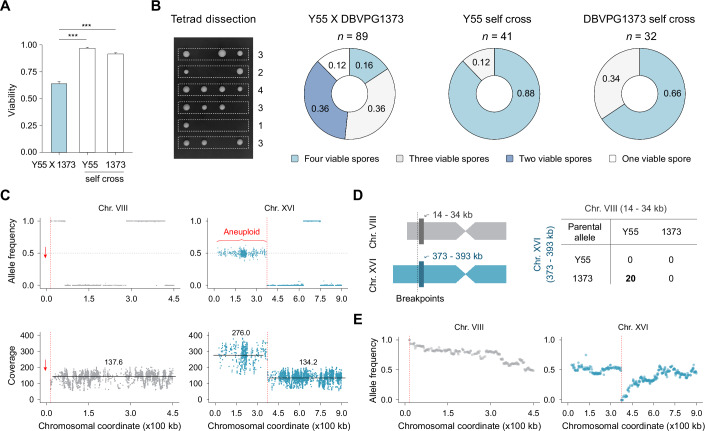
Hybrid produces inviable spores that affect allele frequency in progeny. (**A**) The spore viability of tetrads from hybrids of Y55 and DBVPG1373 and self-crosses of each parental strain. Bars represent the mean ± SD of three independent replicate crosses (*n* = 3 biological replicates). One-way ANOVA indicated a significant difference among groups (*p* = 3.15 × 10^−6^), followed by Tukey HSD post hoc test; *** indicates *p* < 0.001. Exact Tukey HSD post hoc test *p* values were: Y55×DBVPG1373 vs. Y55 self-cross, *p* = 3.73 × 10^−6^; Y55×DBVPG1373 vs. DBVPG1373 self-cross, *p* = 1.06 × 10^−5^. (**B**) Summary of tetrads producing different numbers of viable spores in hybrid and self-crosses of Y55 and DBVPG1373. *n* represents the total number of tetrads dissected in each group. (**C**) Allele frequencies and sequencing coverage of SNP markers in XVI-A spores. The average coverages in chromosomes VIII and XVI are labeled. The red arrow indicates the lost region on chromosome VIII. The red dashed lines indicate the breakpoints. (**D**) Summary of parental alleles in specific regions on chromosomes VIII and XVI in XVI-A spores. The red regions in the schematic on the left indicate the specific regions on chromosomes VIII and XVI. (**E**) Allele frequency variations across chromosomes VIII and XVI in a spore population composed of randomly selected spores. The red dashed lines indicate the breakpoints. Source data are available online for this figure.

To test this hypothesis, we evaluated the spore viability of the initial hybrids during meiosis using tetrad dissection. For the hybrids of Y55 (MATa) and DBVPG1373 (MATα), we dissected 89 tetrads and obtained 227 viable spores. The spore viability (63.8%) was significantly lower than that of spores derived from the self-crosses of Y55 and DBVPG1373, which were 94.6 and 91.5%, respectively (Methods, Fig. 2A). Tetrad analysis allowed us to assess spore viability from individual meiotic events. Most tetrads from the self-crosses produced four viable spores, with proportions of 87.8% (36/41) for Y55 and 65.6% (21/32) for DBVPG1373 (Fig. 2B). In contrast, only 15.7% (14/89) of the hybrid tetrads generated four viable spores. The majority of hybrid tetrads (71.9%, 64/89) produced three or two viable spores (Fig. 2B). These results support the hypothesis that the observed allele frequency shifts stem from the meiotic process in hybrids. While meiosis generates four spores per tetrad, some spores are inviable, leading to preferential inheritance of certain alleles.

**Figure EV2:**
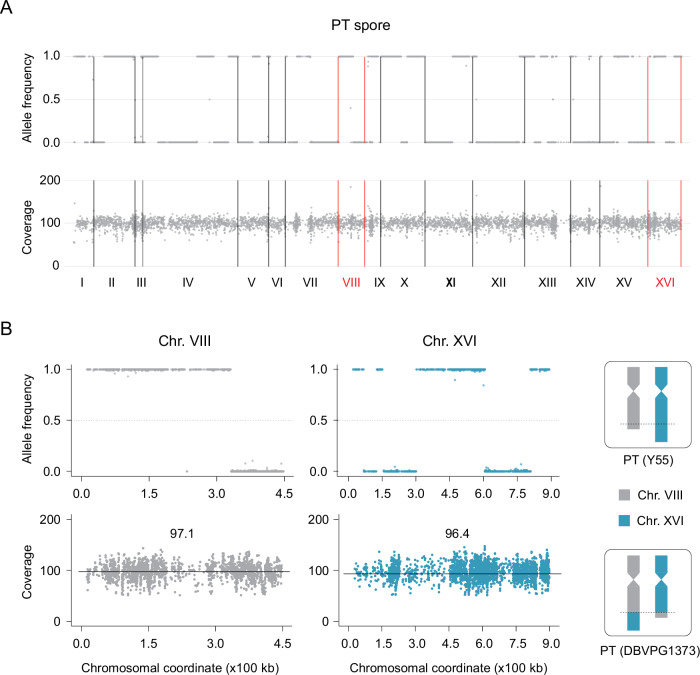
Allele frequencies and sequencing coverage of SNP markers in PT spores. (**A**) The upper panel shows the genome-wide distribution of allele frequencies, and the lower panel shows the corresponding sequencing coverage of a representative PT spore. (**B**) Enlarged views of Chr. VIII and Chr. XVI. The upper panel shows the allele frequencies along Chr. VIII and Chr. XVI, and the lower panels show the corresponding sequencing coverage. The black solid lines on the coverage panels indicate the average coverage, with the exact value shown above. In PT spores, the allele at each genomic position is expected to originate from either Y55 or DBVPG1373, and the sequencing coverage is uniform across the genome.

To further investigate the genotypes of meiotic products, we randomly selected 111 spores from the meiotic products of the initial hybrids for WGS individually (Dataset EV3). Given that each spore is haploid, the allele at each genomic position should originate from either Y55 or DBVPG1373, exclusively. Uniform sequencing coverage across the genome was also expected, as each genomic region should be present in a single copy in the haploid genome. The majority of spores (79.3%, 88/111) exhibited the expected allele frequency distribution and sequencing coverage, which we designated as the parental type (PT) (Fig. EV2).

**Figure EV3:**
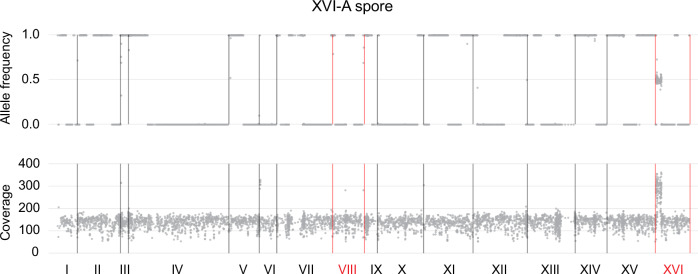
Allele frequencies and sequencing coverage of SNP markers in XVI-A spores. The upper panel shows the genome-wide distribution of allele frequencies, and the lower panel shows the corresponding sequencing coverage of a representative XVI-A spore.

However, a secondary pattern was observed in 18.0% (20/111) of the spores, characterized by heterozygous allele states and approximately double sequencing coverage for a ~370 kb region on the upstream end of chromosome XVI (Figs. 2C and EV3). We classified these spores as XVI-A spores. Another notable feature of XVI-A spores was the loss of a ~15 kb region on the upstream end of chromosome VIII (Fig. 2C). This region contained only a few SNP markers due to its proximity to the chromosome end, and these alleles were absent in XVI-A spores but present in PT spores (Appendix Fig. S5). Interestingly, all XVI-A spores exhibited absolute inheritance of DBVPG1373 alleles in a specific region on chromosome VIII (14–34 kb) and Y55 alleles in a region on chromosome XVI (373–393 kb) (Fig. 2D). This suggests that the combination of Y55 alleles on chromosome VIII and DBVPG1373 alleles on chromosome XVI may be lethal, and the allele states of chromosomes VIII and XVI were coupled in XVI-A spores due to a potential rescue mechanism. Among the remaining three spores, one exhibited whole-chromosome aneuploidy of chromosome VIII, and the other two displayed segmental aneuploidy of chromosome XVI (Appendix Fig. S6).

We then examined the parental allele frequencies within the XVI-A spore population (Methods). Coupled allele frequency variations on chromosomes VIII and XVI were also observed, consistent with the results from bulk sequencing of the meiotic product in the first round (Fig. 2E). However, no consistent signals were detected in the related regions on chromosomes III, X, or XII, indicating these regions were not linked with XVI-A spores (Appendix Fig. S7).

To rule out potential bias introduced by mating type, we performed a reciprocal cross of DBVPG1373 (MATa) and Y55 (MATα) and randomly selected 101 spores for WGS (Dataset EV4). The same patterns were observed in the spore population derived from the reciprocal cross (Appendix Fig. S8). Furthermore, we analyzed the mating types of the sequenced spores and found no significant deviation from the expected 1:1 ratio in either the Y55 × DBVPG1373 cross (62 MATa vs. 49 MATα, chi-square test: *p* = 0.22) or the reciprocal cross (49 MATa vs. 52 MATα, chi-square test: *p* = 0.76), confirming that viability and genomic composition of spores are independent of mating type (Dataset EV5).

### Allele frequency changes on chromosomes VIII and XVI are linked to a reciprocal translocation

**Figure 3:**
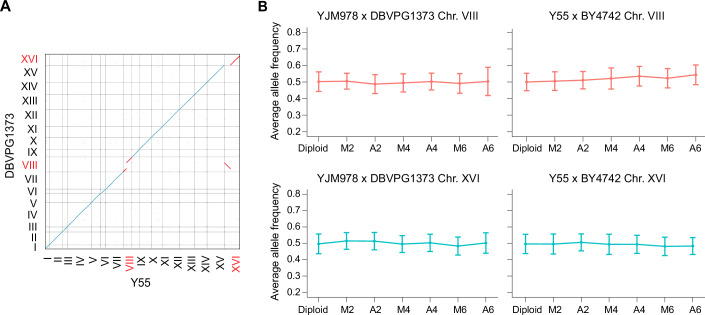
Allele frequency changes on chromosomes VIII and XVI are linked to a reciprocal translocation. (**A**) DBVPG1373 assembled chromosomes (vertical axis) align in a dot matrix plot to Y55 assembled chromosomes (horizontal axis). The red lines indicate the reciprocal translocation between chromosome VIII and XVI in DBVPG1373. (**B**) Dynamics of allele frequency variation in the hybrid strains YJM978 (MATa) × DBVPG1373 (MATα) and Y55 (MATa) × BY4742 (MATα). Allele frequencies were quantified for a defined region on VIII (14,867–54,867 bp) and XVI (373,654–413,654 bp). Average allele frequency at each time point is presented as mean ± SD from three biological replicates. Mn indicates the *n*th round of sporulation products, and An indicates the *n*th round of mitotic growth products. Source data are available online for this figure.

We re-sequenced the genomic DNA of the parental strains Y55 and DBVPG1373 using Nanopore sequencing and reassembled their genomes. Through this analysis, we identified a reciprocal translocation (RT) between positions 1–14,867 bp on chromosome VIII and 1–373,694 bp on chromosome XVI in DBVPG1373, consistent with previous reports on other *S. cerevisiae* strains (Pérez-Ortın et al, 2002) (Fig. 3A). This structural rearrangement explains the observed coupling of chromosomes VIII and XVI in the hybrid progeny. In contrast, comparison of the regions on chromosomes III, X, and XII revealed only minor structural differences in DBVPG1373 relative to Y55: a 337 bp insertion on chromosome III and deletions of 341 bp and 337 bp on chromosomes X and XII, respectively. These findings suggest that even small structural differences may cause allele frequency bias.

To confirm that the observed allele frequency changes on chromosomes VIII and XVI are specifically associated with the reciprocal translocation, we performed additional control crosses using YJM978 × DBVPG1373 and Y55 × BY4742. In these crosses, YJM978 shares the same chromosomal configuration as DBVPG1373 on chromosomes VIII and XVI, and BY4742 shares the same configuration as Y55, such that neither cross carries heterozygous reciprocal translocations. We did not observe significant allele frequency changes near the breakpoints in these control crosses, whereas such changes were detected only in crosses carrying the translocations (Fig. 3B). These results indicate that the observed allele frequency changes on chromosomes VIII and XVI are specifically associated with the presence of chromosomal rearrangements rather than other factors.

### Selection favors crossover-associated adjacent-1 segregation

**Figure EV4:**
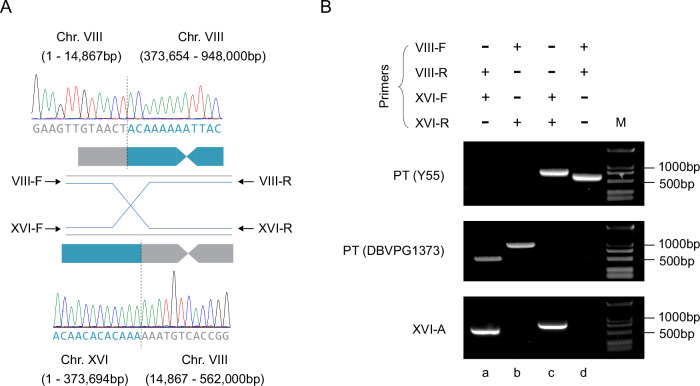
Schematic of the PCR-based karyotype analysis method. (**A**) To determine the chromosomal combinations of spores, four primers (VIII-F, VIII-R, XVI-F, and XVI-R) were used to amplify regions flanking the chromosomal breakpoints on Chr. VIII and Chr. XVI. The dashed lines indicate the position of chromosomal breakpoints and the Sanger sequencing results of these regions are shown on the figure. (**B**) The PCR result of different primer sets. If only VIII-F + VIII-R and XVI-F + XVI-R or VIII-F + XVI-R and XVI-F + VIII-R amplified successfully, the karyotype should be PT (Y55 or DBVPG1373). If only XVI-F + XVI-R and XVI-F + VIII-R amplified successfully, the karyotype is XVI-A. The PCR marker is DS2000, and the 500 and 1000 bp bands are labeled. Source data are available online for this figure.

We therefore focused on the RT between chromosomes VIII and XVI for further analysis. We developed a PCR-based karyotype method for efficient verification of parental alleles and chromosomal combinations in regions flanking the breakpoints on chromosomes VIII and XVI in individual gametes. This method enabled us to categorize meiotic products into previously identified types (PT and XVI-A spores) for each meiotic event (Methods, Fig. EV4). We validated our method through WGS of 16 spores from six tetrads, which demonstrated perfect concordance between PCR-based determinations and sequencing-derived chromosomal states (Appendix Fig. S9).

**Figure 4:**
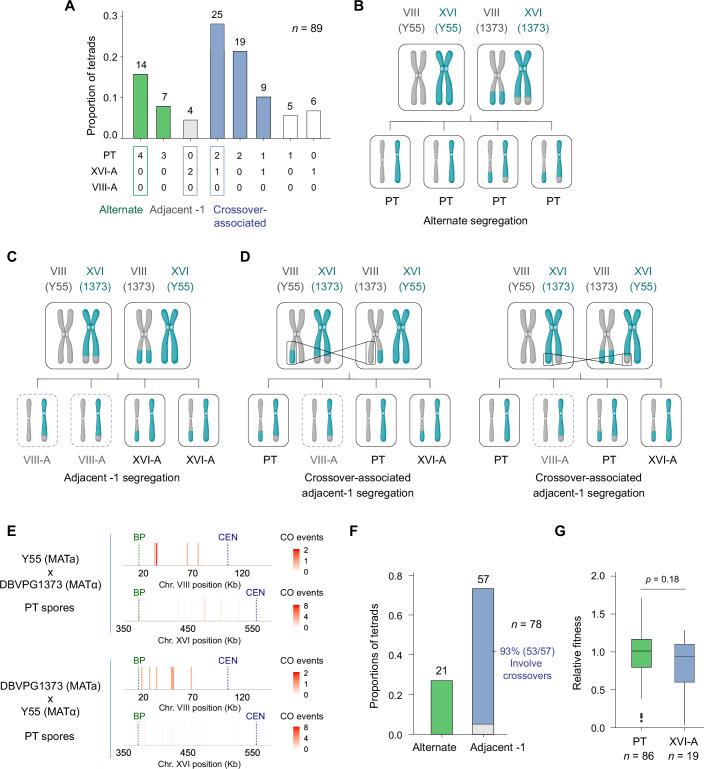
Adjacent segregation of the quadrivalent leads to unequal segregation. (**A**) Summary of tetrad dissection, including the number of viable spores and their types within a single tetrad. Tetrads are grouped based on the number of viable spores and spore types. Numbers above each bar indicate the count of tetrads for that category. A total of *n* = 89 tetrads were analyzed. (**B**–**D**) Analysis of 89 tetrads reveals three distinct segregation patterns. In alternate segregation (**B**), the states of chromosomes VIII and XVI correspond to either Y55 or DBVPG1373, resulting in four viable PT spores. In adjacent-1 segregation (**C**), two XVI-A spores are paired with two complementary VIII-A spores, but the VIII-A spores are inviable. In crossover-associated adjacent-1 segregation (**D**), one tetrad produces two PT spores, one XVI-A spore, and one inviable VIII-A spore. This pattern arises from a crossover event in the interstitial region (between the centromere and translocation breakpoint) of either chromosome VIII or XVI. (**E**) Crossover distribution in PT spores. Crossover positions in the interstitial regions of chromosomes VIII and XVI are shown for the forward cross (Y55 × DBVPG1373, *n* = 88 PT spores) and reciprocal cross (DBVPG1373 × Y55, *n* = 84 PT spores). (**F**) Proportions of tetrads undergoing alternate segregation and adjacent-1 segregation. When calculating the number of tetrads, the random loss of one spore during tetrad dissection or reduced fitness under certain genotype combinations is taken into account. As a result, tetrads with one fewer viable spore were also included in the corresponding segregation statistics. (**G**) The relative fitness of PT and XVI-A spores. Significance between groups was examined by the two-tailed Wilcoxon rank-sum test (*p* = 0.18). The center lines of the boxplot indicate the median, the lower and upper bounds of the box indicate the 25th and 75th percentiles, respectively, and whiskers extend to the minimum and maximum values within 1.5 times the interquartile range, with outliers shown as individual points. *n* = 86 for PT spores and *n* = 16 for XVI-A spores. Source data are available online for this figure.

Analysis of 89 tetrads uncovered three distinct segregation patterns of quadrivalents (Fig. 4A; Dataset EV6). In contrast to previous reports showing predominant alternate segregation, alternate segregation accounted for 15.7% (14 tetrads) of cases here, consistently yielding four PT spores (Fig. 4A,B). Importantly, the expected adjacent-1 segregation products (two XVI-A spores paired with two complementary VIII-A spores) were completely absent, likely due to the inviability of VIII-A spores caused by loss of 36 essential genes on chromosome XVI (Liu et al, 2015) (Fig. 4C). Instead, we identified four tetrads producing two XVI-A spores, indicating canonical adjacent-1 segregation (Fig. 4A).

Most remarkably, we discovered a prevalent novel pattern (28.1%, 25 tetrads) characterized by two PT spores and one XVI-A spore (Fig. 4A). The most parsimonious explanation for this pattern is that it results from adjacent-1 segregation coupled with a crossover event occurring in the interstitial region between the centromere and the breakpoint of either chromosome VIII or XVI. Such a crossover would alter the linkage between the centromere and the translocation breakpoint, resulting in the production of two PT spores, one XVI-A spore, and one inviable VIII-A spore (Fig. 4D).

To test this hypothesis, we analyzed the genomic composition of randomly selected spores to identify recombination events. Specifically, we searched for genotype transitions indicative of crossovers (i.e., a switch in parental genotype extending from the recombination site to the breakpoint) in chromosomes VIII and XVI in PT spores, which could arise either from alternate segregation or as viable products of crossover-associated adjacent-1 segregation (Methods, Fig. 4E). Among 88 PT spores, five and nine spores exhibited valid crossovers on chromosomes VIII and XVI, respectively. Similarly, in 84 PT spores derived from the reciprocal cross, we identified 7 and 14 crossovers on chromosomes VIII and XVI, respectively. These findings provide molecular evidence supporting the occurrence of crossovers in the predicted intervals, with a higher frequency observed on chromosome XVI. The distribution of crossover sites appeared random, with no significant positional bias (Fig. 4E). Compared to canonical adjacent-1 segregation, which producing two XVI-A spores, this segregation pattern yields a greater number of viable spores.

Considering the random loss of one spore during tetrad dissection or reduced fitness under certain genotype combinations, tetrads producing three PT spores could be attributed to alternate segregation. Similarly, tetrads producing two PT spores or one PT spore alongside one XVI-A spore could be attributed to adjacent-1 segregation with crossover events. In total, our analysis indicates that adjacent-1 segregation events (57 tetrads) occurred 2.7-fold more frequently than alternate segregation (21 tetrads), with 53 of 57 adjacent-1 cases involving crossovers (Fig. 4F). This unexpected predominance of adjacent-1 segregation challenges conventional models of RT segregation (Loidl et al, 1998; McKinlay Gardner et al, 2018). We measured the fitness of PT and XVI-A spores and found no significant differences between these two spore types (Fig. 4G). We also performed eight independent pairwise competitive growth experiments between PT and XVI-A spores, and the results showed that the proportions of the two spore types remained relatively stable over four passages of cultivation (Methods, Appendix Fig. S10). These results strongly suggest that selection acts on the segregation pattern of the quadrivalent itself.

### Selection operating at the quadrivalent level may be prevalent across species

**Figure 5:**
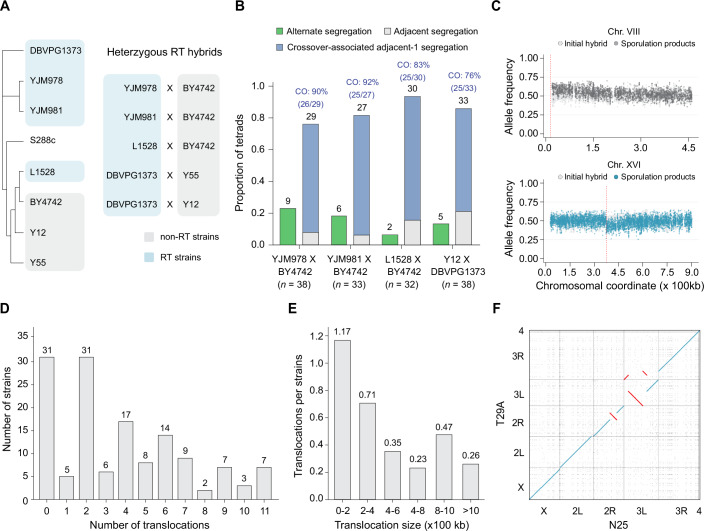
Unequal segregation associated with chromosomal translocations is prevalent. (**A**) Phylogenetic tree of S. cerevisiae strains used in this study. The right panel illustrates four additional crosses conducted between these strains. (**B**) Proportions of tetrads undergoing alternate segregation and adjacent-1 segregation in these four crosses. CO represents crossover events. (**C**) Allele frequency variations across chromosomes VIII and XVI in the meiotic products (colored) and the initial hybrid (gray) from the cross between Y12 and DBVPG1373. (**D**) Distribution of translocation across 140 *S. cerevisiae* strains. (**E**) Distribution of translocation with different sizes across 140 *S. cerevisiae* strains. (**F**) Dot matrix plot aligning T29A assembled chromosomes (vertical axis) with N25 assembled chromosomes (horizontal axis). The red lines indicate translocations. Source data are available online for this figure.

To exclude the effect of genetic distance between parental strains, we selected five additional *S. cerevisiae* strains to confirm this biased segregation pattern (Dataset EV7). The genomic similarity among these strains ranges from 0.98 to 0.99. Three strains (YJM978, YJM981, and L1528) carried identical RTs on chromosomes VIII and XVI as DBVPG1373, while Y12 and BY4742 matched Y55’s configuration. We conducted four additional crosses between these strains and dissected 30–40 tetrads from each hybrid (Fig. 5A; Dataset EV7). The chromosomal status of chromosomes VIII and XVI in each spore was determined using the PCR-based method. These heterozygous RT hybrids exhibited spore viability patterns closely resembling those observed in the Y55×DBVPG1373 cross (Appendix Fig. S11; Dataset EV8). Tetrads producing three viable spores were more frequent than those producing four viable spores.

Importantly, adjacent-1 segregation consistently occurred at a significantly higher frequency than alternate segregation across all crosses. Among the adjacent-1 segregation events, crossover-associated patterns were predominant, accounting for 76 to 92% of cases (Fig. 5B). We also examined the parental allele frequency of the meiotic products through WGS for each group. Coupled allele frequency variations on chromosomes VIII and XVI were also observed in all groups, further reinforcing the conserved nature of this segregation bias (Fig. 5C; Appendix Fig. S12).

We have demonstrated how RT generates non-random meiotic segregation patterns, with selection favoring crossover-associated adjacent-1 segregation over alternate segregation at the quadrivalent level, leading to rapid changes in allele frequencies within populations. Beyond their role in species formation (Rieseberg, 2001), translocations are also highly prevalent at the intraspecies level across diverse organisms (Damas et al, 2021; Mérot et al, 2020). To investigate this, we collected data on translocation events among intraspecies strains of *S. cerevisiae* and *Drosophila melanogaster* (Long et al, 2018; O’Donnell et al, 2023). In *S. cerevisiae*, across 140 strains, the average translocation frequency per genome is ~4 (Fig. 5D). Even for large translocations (>1000 kb), one translocation is observed for every four strains (Fig. 5E). Similarly, in *Drosophila melanogaster*, one of five analyzed strains exhibited a distinct translocation (Fig. 5F). Given the prevalence of translocation, the segregation bias observed in our study may have implications for understanding allele frequency dynamics in natural populations where such structural variants are segregating.

## Discussion

Evolutionary forces can operate at multiple levels simultaneously, from individual gametes to entire populations. Understanding these meiotic-level selection processes is crucial for comprehending the full spectrum of evolutionary dynamics and their implications for population genetic structure and speciation processes. In this study, we reveal rapid genomic evolution driven by a biased segregation pattern through serial cross-meiotic experiments in yeast hybrids. Such bias not only affects the number of viable gametes but also reshapes the genomic composition of the population. Dramatic allele frequency shifts were observed extending approximately 300 kb from RT breakpoints on chromosomes VIII and XVI within a short period (Fig. 3E), indicating that the RT acts as a powerful distorter of Mendelian segregation.

Chromosomal rearrangements such as RTs are widespread across diverse organisms (Damas et al, 2021; Mérot et al, 2020). While outcrossing rates in *S. cerevisiae* have been estimated to be as low as once per 50,000 generations under certain assumptions (Ruderfer et al, 2006), recent studies suggest that outcrossing may occur at considerably higher frequencies under natural conditions. When entire dense populations of sporulated yeast germinate simultaneously, outcrossing can occur at rates of 10–25% (Murphy and Zeyl, 2010). Furthermore, population genomic analyses of 1011 *S. cerevisiae* isolates have revealed mosaic genome structures in 150 strains, indicative of historical outcrossing and hybridization events (Peter et al, 2018). These findings suggest that opportunities for generating heterozygous reciprocal translocations through outcrossing are more frequent than previously appreciated.

In quadrivalents formed by heterozygous RT carriers, alternate segregation is typically the most frequently observed mode, followed by adjacent-1 segregation (Benet et al, 2005; Loidl et al, 1998; Oliver-Bonet et al, 2001; Verdoni et al, 2021). In some of these studies, adjacent-1 segregation products may be underrepresented among viable progeny when both translocated segments contain essential genes, potentially contributing to the observed predominance of alternate segregation. In our system, XVI-A spores remain viable because the small translocated fragment on chromosome VIII does not contain essential genes, allowing us to detect adjacent-1 segregation events that might otherwise be masked. However, our methodological approach, combining tetrad dissection with PCR-based karyotype analysis, revealed a strikingly different segregation pattern preference. In Y55 (MATa) and DBVPG1373 (MATα) hybrids, we observed an alternate: adjacent-1 segregation ratio of 1:2.7. This unusual pattern does not appear to result solely from the relatively short length of the translocated segment on chromosome VIII. In a previous study of heterozygous translocations involving t(I,VI) in yeast, breakpoints were mapped between positions 10–14 kb on *I*L and 133–145 kb on *VI*L (Loidl et al, 1998). Importantly, there are no essential genes within the 1–15 kb region on *I*L. Although the translocated segment length in t(I,VI) is comparable to that in our study, predominant alternate segregation was observed.

Similar patterns have been observed in pooled offspring populations, with most studies focusing on human sperm populations. In humans, RTs have an estimated frequency of ~1 in 500 (Verdoni et al, 2021). Benet et al, analyzed 237,042 spermatozoa from 46 RT carriers using fluorescence in situ hybridization across various chromosomal regions, and found that alternate segregation was most frequent (40.5%), followed by adjacent-1 (26.6%) (Benet et al, 2005). A separate WGS study of 3951 human blastocysts from RT carriers, where RTs spanned all human chromosomes, corroborated these findings (Yu et al, 2023). This discrepancy between our findings and previous studies likely arises from the difference between examining pooled gametes versus analyzing independent meiotic events. Theoretically, all PT spores should be derived from alternate segregation, while XVI-A spores should result from adjacent-1 segregation. In random spore populations, we observed a predominance of PT spores compared to XVI-A spores (79.3 vs. 18.0%), which could easily be misattributed to alternate segregation dominance if we hadn’t examined spores from the same tetrad separately. These results suggest that we may have overestimated the frequency of alternate segregation in RT carriers. On the other hand, selection may enhance offspring viability by acting on other aspects of the meiotic process.

The systematic bias toward crossover-associated adjacent-1 segregation observed in our yeast system has profound evolutionary implications that extend far beyond traditional models of meiotic selection, especially meiotic drive. Classical meiotic drive systems typically favor specific alleles through gametic competition or post-meiotic mechanisms, as exemplified by several well-documented cases across diverse taxa: the spore killer gene *wtf4* in the fission yeast *Schizosaccharomyces kambucha* (Nuckolls et al, 2017), Het-s in the fungus *Podospora anserine* (Dalstra et al, 2003), the sperm killer of segregation distorter (SD) in *Drosophila* (Courret et al, 2019), and the *t*-haplotype in mice (Ardlie, 1998). In contrast, RT-associated segregation distortion operates through chromosomal-level mechanisms that simultaneously affect multiple linked loci across extended genomic regions. The coordinated allele frequency changes observed in these regions reflect selection for tight linkage, leading to the consolidation of large gene blocks into single segregating units.

Although XVI-A spores in our study carried a ~300 kb duplication and a ~15 kb deletion, such chromosomal imbalances may nonetheless persist in populations over extended periods. Population genomic analyses of 1011 *S. cerevisiae* isolates revealed that 19.1% of natural strains contain chromosomal aneuploidies and large segmental duplications (Peter et al, 2018). Furthermore, genome structural analysis of 140 telomere-to-telomere assemblies identified 62 strains harboring deletions exceeding 15 kb (O’Donnell et al, 2023). These observations indicate that large-scale chromosomal imbalances, including those potentially arising from adjacent-1 segregation, can be tolerated and maintained in natural populations.

Our marker-based assay showed that diploid re-formation during the cross-sporulation cycle is dominated by inter-tetrad mating, with intra-tetrad mating accounting for the remaining fraction. We therefore explicitly evaluated the impact of intra-tetrad mating on allele frequency dynamics. These analyses showed that intra-tetrad mating moderates the effect by slowing allele frequency change relative to a fully inter-tetrad or fully random mating regime (Appendix Fig. S13, Appendix Methods). Nevertheless, it does not alter the central conclusion that translocation-associated selection during meiosis can drive rapid allele frequency change in this system.

In conclusion, our findings suggest that segregation bias associated with inter-chromosomal translocation may represent an important factor influencing allele frequency dynamics. Our work demonstrates that meiotic-level selection can rapidly reshape allele frequencies through chromosomal mechanisms. While we identified the chromosomal basis for dramatic allele frequency changes on chromosomes VIII and XVI, we were unable to identify a specific mechanism that could account for the observed allele frequency shifts on chromosomes III, X, and XII. The structural variations in these regions involve only several hundred base pairs of sequence differences, requiring additional high-resolution genomic approaches to establish definitive causal relationships. Despite these limitations, our findings highlight evolutionary dynamics at the meiotic level that may help connect chromosomal biology and population genetics, providing insights into the forces that could contribute to genetic diversity in natural populations.

## Methods

**Table:** Reagents and tools table

Reagent/resource	Reference or source	Identifier or catalog number
**Experimental models**		
Y55 (MATa ho::HygMX ura3::KanMX)	National Collection of Yeast Cultures	NCYC 3588
DBVPG1373 (MATα ho::HygMX ura3::KanMX)	National Collection of Yeast Cultures	NCYC 3620
Y12 (MATa ho::HygMX ura3::KanMX)	National Collection of Yeast Cultures	NCYC 3605
YJM978 (MATa ho::HygMX ura3::KanMX)	National Collection of Yeast Cultures	NCYC 3592
YJM981 (MATa ho::HygMX ura3::KanMX)	National Collection of Yeast Cultures	NCYC 3593
L1528 (MATa ho::HygMX ura3::KanMX)	National Collection of Yeast Cultures	NCYC 3599
BY4742 (MATα his3Δ1 leu2Δ0 met15Δ0 ura3Δ0)	American Type Culture Collection	ATCC 4010734
**Recombinant DNA**		
**Antibodies**		
**Oligonucleotides and other sequence-based reagents**		
PCR primers	This study	Dataset EV9
**Chemicals, enzymes and other reagents**		
Yeast extract	Thermo Scientific^TM^ OXOID^TM^	Cat. No. LP0021B
Peptone	HUANKAI Microbial	Cat. No. 050170B
Glucose anhydrous	Guangzhou Chemical Reagent Factory	Cat. No. IC08
Agar	MYM Biological Technology	Cat. No. MA0451
Potassium acetate	Sigma-Aldrich	Cat. No. P1190
Ura minus media	FunGenome	Cat. No. YGM003A-3
**Software**		
Flye v2.9.5	https://github.com/mikolmogorov/Flye	
Medaka v2.0.1	https://github.com/nanoporetech/medaka	
NextPolish v1.4.1	https://github.com/Nextomics/NextPolish	
Ragout v2.3	https://github.com/mikolmogorov/Ragout	
Mummer v4.0.0	https://github.com/mummer4/mummer	
Trimmomatic v0.39	https://github.com/usadellab/Trimmomatic	
BWA v0.7.17-r1198	https://github.com/lh3/bwa	
Samtools v1.13	https://github.com/samtools/samtools	
GATK v4.3.0.0	https://github.com/broadinstitute/gatk	
bcftools v1.19	https://github.com/samtools/bcftools	
**Other**		

### Yeast strains

This study included nine *Saccharomyces cerevisiae* strains, with their genotypes listed in Dataset EV7. All strains are heterothallic. Except for BY4741 (MATa, *his3*, *leu2*, *met15*, and *ura3*) and BY4742 (MATα, *his3*, *leu2*, *met15*, and *ura3*), other strains were purchased from the National Collection of Yeast Cultures. The mating type of each strain was confirmed via PCR using two primer sets: A-F + MAT-R and alpha-F + MAT-R. Amplification with only one primer set indicated a haploid clone, while amplification with both sets suggested a diploid. Primer sequences are provided in Dataset EV9.

### Successive cross-meiosis experiment procedure

To generate hybrid or parental diploids, haploid strains of opposite mating types were crossed. Haploid strains were initially streaked on YPD plates (1% yeast extract, 1% peptone, 2% dextrose, and 1.5% agar), and single colonies were picked and inoculated into YPD liquid medium. After overnight incubation, two corresponding haploid strains were mixed 1:1 and cultured for 48 h in fresh YPD medium. A 100 μL aliquot of the culture was collected, washed with sterile water, and plated onto fresh YPD plates. Diploid clones were identified via PCR using the aforementioned primers. Specifically, single clones were picked and lysed with 0.2 U of Lyticase (Sigma-Aldrich L2524) and incubated at 37 °C for 30 min following by 98 °C for 5 min. The lysate served as the PCR template. Three independent diploid clones were obtained per cross for subsequent experiments.

Diploid clones, after sampling for genomic extraction as initial controls, were then subjected to the sporulation process, a process that induces meiosis and the formation of haploid spores. In detail, the diploid clones cultured in 5 mL YPD medium were collected, then washed with sterile water and transferred to 15 mL sporulation medium (1% yeast extract, 10% potassium acetate, 0.5% dextrose, and 0.04% uracil), and incubated at 25 °C, 120 rpm for 7 days. After 7 days, 3 mL culture (~10^8^ cells/mL) were collected for genomic DNA extraction and 1 mL of the culture was harvested, washed and transferred to 15 mL YPD liquid medium for mitotic growth for 16 h. Following mitotic growth, 3 mL of cells were collected for genomic DNA extraction, and the remaining cells were transferred to 15 mL fresh YPD medium for random mating. After 72 h, cells were subjected to another round of sporulation using the same procedure. This cross and meiosis process was repeated for five rounds.

### Random spore isolation

After sporulation, 1 mL of the culture (~10^8^ cells) was harvested and treated with 50 U lyticase dissolved in Y1 buffer (1 M sorbitol and 0.1 M EDTA, pH 7.4) for 30 min at 30 °C in order to break the cell wall of tetrad asci. The cells were then washed and plated onto YPD plates, and incubated at 30 °C for two days. Single colonies were lysed, and haploid spores were identified via PCR using the primers described earlier.

### Fluorescent marker assay for mating randomness

Four viable haploid spores were obtained from a single tetrad of the hybrid Y55 (MATa) and DBVPG1373 (MATα) via tetrad dissection (see the “Tetrad dissection and calculation of spore viability” section). Mating types were identified by PCR (see the “Yeast strains” section), with two spores classified as MATa and two as MATα. For MATa spores, the NatMX6 cassette was integrated to obtain nourseothricin resistance. For MATα spores, GFP reporters were integrated with the URA3 marker. In addition, two haploid MATα spores from other tetrads were obtained in which mScarlet was integrated with the URA3 marker. Fluorescent expression was confirmed by fluorescence microscopy. These haploid strains were cultured separately overnight in YPD medium at 30 °C until saturation. The MATa and MATα cells were then mixed at a 1:1 ratio and allowed to mate for 48 h. The mating culture was subsequently diluted 1:300 and transferred to SC-URA (NAT⁺) medium, followed by incubation at 30 °C until saturation. Three independent replicates were performed.

To determine the ratio of the fluorescent diploids, 100 uL of the culture was harvested, washed twice with PBS, and diluted 100-fold to prepare the single-cell suspension for flow cytometry. Analysis was performed using the CytoFLEX LX flow cytometer (Beckman Coulter). GFP signals were detected in the B525-A channel (488 nm excitation, 525/40 nm emission), and mScarlet signals were detected in the Y610-A channel (561 nm excitation, 610/20 nm emission). A total of ~10,000 cells were collected for each sample. Yeast populations were gated based on forward scatter (FSC-A) and side scatter (SSC-A) to exclude debris and aggregates. Data were analyzed using CytExpert (Version 2.6) to quantify the proportions of diploid cells expressing GFP and mScarlet.

### Tetrad dissection and calculation of spore viability

Tetrad asci were digested as described in the random spore section, and then streaked onto a YPD plate for tetrad dissection using the MSM400 dissection microscope (Singer Instrument Company Ltd). Spores were grown on YPD plates and cultured for 48 h. One tetrad generates four spores. Viable spores formed colonies, and we carefully recorded the spore viability for each tetrad.

For each cross, spore viability was determined by calculating the ratio of viable spores to the total number of spores dissected. The viability of the self-cross of parental strains was used as a control for the corresponding hybrids.

### Genome extraction and sequencing

For meiotic division and mitotic growth products, 4 mL of the culture (~4 × 10^8^ cells) was collected, then washed with sterile water. Then, cells were performed genomic DNA extraction using the ALFA-SEQ Magnetic Yeast DNA Kit according to the manufacturer’s instruction (Findrop Biosafety technology (Guangzhou) Co.Ltd). Then the genomes were sequenced using the paired-end strategy on the DNBSEQ-T7RS platform at GenePlus and Genewiz by standard procedure, achieving an average coverage of ~100× across all samples.

Additionally, the genomes of strains Y55 (MATa) and DBVPG1373 (MATα) were extracted and sequenced on the DNBSEQ-T7RS platform at GenePlus, as well as through third-generation sequencing on the PromethION platform (Oxford Nanopore Technologies) at the Genome Center of Grandomics (Wuhan, China). For four other *S. cerevisiae* strains (YJM978, YJM981, L1528, Y12), genomic DNA was extracted and sequenced on the DNBSEQ-T7RS platform at GenePlus following standard procedures.

### De novo genome assembly and structure variation detection

Raw fastq files from Oxford Nanopore were used to assemble the genome draft using Flye (version: 2.9.5) (Kolmogorov et al, 2019). First, the assemblies were polished by Medaka (version: 2.0.1; github.com/nanoporetech/medaka) for one round using the same fastq data used for assembly. The Nanopore polished assemblies were then polished by NextPolish (version: 1.4.1) (Hu et al, 2020) for four rounds using NGS data. The hybrid-polished contigs were scaffolded against the S288C reference genome (GCF_000146045.2) using Ragout (version: 2.3) (Kolmogorov et al, 2018), and then manually checked to correct unscaffolded contigs to generate a complete assembly. The Y55 assembly was aligned against the DBVPG1373 assembly using Mummer (version 4.0.0) (Marçais et al, 2018) to detect structural variations.

### NGS read processing

The raw sequencing data of the WGS was processed using Trimmomatic (version 0.39) (Bolger et al, 2014) for quality control. After removing adapters, the reads with an average quality score less than 15 and shorter than 36 bases were filtered using the “SLIDINGWINDOW:4:15, -MINLEN 36” option. The average total reads were ~4 M. High-quality reads were aligned to the S288C reference using BWA mem (version 0.7.17-r1198, with “-M” option) (Li, 2013). The BAM files were sorted with Samtools (version 1.13) and duplicate reads were removed with GATK MarkDuplicates (version 4.3.0.0) (Van der Auwera and O’Connor, 2020).

### Detection of SNP markers between parental strains

For Y55 and DBVPG1373, SNP markers were identified using their de novo assembled genomes. For other strains, NGS data were used. Genomes of each parental strain were aligned to the S288C reference genome (version R64-1-1; http://www.yeastgenome.org), and SNP calling was performed using bcftools (version 1.19) (Danecek et al, 2021). The mapping quality was restricted to 60 with “--min-MQ 60” to filter out low-quality mappings. Detected SNPs differing between the two parental strains were designated as SNP markers.

### Allele frequency analysis

For bulk-seq data from meiotic division and mitotic growth products at each round, NGS data were processed as described above. The quality-controlled data were aligned to S288C, and then the SNP locus detected in the parental strains were selected. The mapping quality of these sites was also restricted to 60. The allele frequency at each polymorphic position was calculated by dividing the coverage of the RT strains (e.g., DBVPG1373) by the total coverage at that position. The sites with coverage less than 30 were filtered during the following analysis. To detect the variation in allele frequency between the sixth-round meiotic products and initial hybrids, SNPs within 2000 bp were considered as blocks. The Kolmogorov–Smirnov (KS) test was performed between the corresponding blocks, and the *p* values were adjusted for multiple testing by the Holm method. The median position and allele frequencies were used to represent the blocks for visualization. For NGS data of single spores, the allele states at each polymorphic position were analyzed to determine the parental source, and coverage was also calculated. The sites with coverage less than 30 were also filtered during the analysis.

### PCR-based karyotype analysis

To determine the chromosomal combinations of spores, four primers (VIII-F, VIII-R, XVI-F, and XVI-R) were used to amplify the regions flanking the chromosomal breakpoints of Chr. VIII and XVI. Primer sequences are provided in Dataset EV9.

For each spore dissected from a tetrad, four sets of primers were used for amplification testing, including VIII-F + VIII-R, XVI-F + XVI-R, VIII-F + XVI-R, and XVI-F + VIII-R. If only VIII-F + VIII-R and XVI-F + XVI-R amplified successfully, the chromosomal combination pattern should be PT (Y55 source). If only VIII-F + XVI-R and XVI-F + VIII-R amplified successfully, the chromosomal combination was PT (DBVPG1373 source). If only XVI-F + XVI-R and XVI-F + VIII-R amplified successfully, the chromosomal combination was classified as XVI-A. Finally, if only VIII-F + VIII-R and VIII-F + XVI-R successfully amplified, the chromosomal combination was assigned as VIII-A. These primers were applicable to all crosses performed in this study.

### Crossover event detection

We scanned SNP markers within the interstitial intervals, which were defined as the regions between the translocation breakpoint and the centromere of chromosomes VIII and XVI. The interstitial interval on chromosome VIII extends from the breakpoint at 14,867 bp to CEN8 (105,586–105,703 bp), and on chromosome XVI from the breakpoint at 373,694 bp to CEN16 (555,957–556,073 bp). A candidate recombination event was identified if the genotype at the centromere differed from the genotype at the translocation breakpoint. To ensure accuracy, all candidate events underwent manual inspection to exclude complex gene conversion events that did not alter the linkage between the centromere and the breakpoint. Consequently, a valid crossover event was defined as any event resulting in a reciprocal exchange of flanking markers, including both simple single crossovers and crossovers associated with adjacent gene conversion tracts.

### Growth rate measurement and pairwise competition experiment

A total of 88 PT spore clones and 20 XVI-A spore clones were grown in YPD medium to saturation at 30 °C for 2 days, then diluted 1:100 into 100 μL fresh YPD medium in 96-well plates. Three replicates of each spore were cultured in the same 96-well plate. The plates were incubated at 30 °C with shaking on a LogPhase 600 microplate reader (Agilent BioTek), and absorbance at 600 nm was measured hourly for 24 h until all samples reached saturation. The maximum growth rate (*V*_max_), defined as the maximum slope of each growth curve, was used to estimate the strain growth rate. The relative fitness for each spore was calculated using the formula: $F={2}^{{\rm{relative}}\,{\rm{growth}}\,{\rm{rate}}-1}$, where the relative growth rate is defined as the *V*_max_ of the PT or XVI-A spores divided by that of the parental control (Y55 MATa) (Shen et al, 2022).

Pairwise competition experiments were conducted between the PT and XVI-A strains collected from random spore analysis. The strains were independently grown in YPD medium to the stationary phase. Random pairwise strains with the same mating type were then mixed at a 1:1 ratio and inoculated into fresh YPD medium at a 1:300 dilution. Cells were incubated at 30 °C with shaking for 12 h, corresponding to the exponential growth phase (eight generations). Cultures were subsequently passaged into fresh medium using the same dilution ratio. At each passage, samples were collected for genomic DNA extraction and library preparation, following the procedures described in the “Genome extraction and sequencing” section. To quantify the relative abundance of PT and XVI-A cells at each passage, parental SNP loci were used as genetic markers. Whole-genome sequencing data from each parental strain were subjected to variant calling using bcftools, with parameters identical to those described in the “Detection of SNP markers between parental strains” section. The relative proportions of PT and XVI-A cells of each pair were determined by the mean allele frequencies of the SNP locus from the initial strains.

## Supplementary information

Appendix

Peer Review File

Dataset EV1

Dataset EV2

Dataset EV3

Dataset EV4

Dataset EV5

Dataset EV6

Dataset EV7

Dataset EV8

Dataset EV9

Source data Fig. 1

Source data Fig. 2

Source data Fig. 3

Source data Fig. 4

Source data Fig. 5

Figure EV4 Source Data

Expanded View Figures

## Data Availability

The raw sequence data reported in this paper have been deposited in the Genome Sequence Archive (Genomics, Proteomics & Bioinformatics 2021) in the National Genomics Data Center (Nucleic Acids Res 2022), China National Center for Bioinformation/Beijing Institute of Genomics, Chinese Academy of Sciences (GSA: CRA024135, CRA024336) that are publicly accessible at http://ngdc.cncb.ac.cn/gsa/s/Mb7wb6vG and https://ngdc.cncb.ac.cn/gsa/s/A1f750G7, respectively. Code to run the analyses is available at https://github.com/doorholez/RT_Drive_Script. The source data of this paper are collected in the following database record: biostudies:S-SCDT-10_1038-S44319-026-00820-6.
