# Supplementary material for: Rapid evolution driven by translocation-associated selection during meiosis
Source: EMBO Rep. 2026 Jun 16;27(14):4011–28. doi: 10.1038/s44319-026-00820-6 (PMC13400751; doi:10.1038/s44319-026-00820-6)
Supplement: Supplementary file 1 — Appendix [file 44319_2026_820_MOESM1_ESM.pdf]

Appendix to
“Rapid evolution driven by translocation-associated selection during meiosis”

Table of Contents

Appendix Methods ………………………………………… 1

Appendix Figure S1 ………………………………………… 6

Appendix Figure S2 ………………………………………… 7

Appendix Figure S3 ………………………………………… 8

Appendix Figure S4 ………………………………………… 9

Appendix Figure S5 ………………………………………… 10

Appendix Figure S6 ………………………………………… 11

Appendix Figure S7 ………………………………………… 12

Appendix Figure S8 ………………………………………… 13

Appendix Figure S9 ………………………………………… 14

Appendix Figure S10 ………………………………………… 15

Appendix Figure S11 ………………………………………… 16

Appendix Figure S12 ………………………………………… 17

Appendix Figure S13 ………………………………………… 18

Appendix Methods

Quantitative analysis of intra- and inter-tetrad mating of spores

The Gapp-GFP-URA3 and Gapp-mScarlet-URA3 were integrated into the intergenic region between MRX9 and RTK1 (Chr.IV 405330...405743 bp) in Y55 (MATa) and DBVPG1373 (MATalpha). The GFP-labeled Y55 (MATa) was crossed with WT DBVPG1373 (MATalpha) to obtain a GFP/- heterozygous diploid (GO), and crossed WT Y55 (MATa) with mScarlet-integrated DBVPG1373 (MATalpha) to obtain an mScarlet/- diploid (MO). At the same time, the above procedure was repeated using Y55 (MATalpha) and DBVPG1373 (MATa) strains to exclude the effect of reciprocal crosses. Three biological replicates were performed for each experiment.

GO and MO strains were cultured separately in 5 mL YPD medium at 30 °C, 250rpm shaking overnight. Cells were collected and washed, then transferred into 15 mL KAC sporulation medium. Sporulation was induced at 25 °C, 120 rpm for 7 days. After sporulation, cells were plated for random spore analysis, and the numbers of haploid and diploid clones were determined by PCR genotyping. Subsequently, 500 µl of each sporulated product was mixed in 15 mL YPD medium and incubated for 16 hours for vegetative growth. Cells were then transferred to 15 mL fresh YPD medium and incubated for 72 hours for mating. After mating, cells were plated again, and the numbers of haploid and diploid clones were determined by PCR genotyping. The proportion of GM diploids (GFP/mScarlet) carrying both GFP and mScarlet markers among diploid clones was determined by PCR. The proportions of intra- and inter-tetrad mating were estimated from the diploid-to-haploid ratios measured at the two plating steps as well as the proportion of GM diploids.

Estimating the proportion of intra-/inter-tetrad mating

To quantify the relative contributions of intra-tetrad and inter-tetrad mating, GO (GFP/-) and MO (mScarlet/-) strains were sporulated separately and then mixed in equal proportions in rich medium to allow vegetative growth and mating. The proportion of GM (GFP/mScarlet) diploids was subsequently determined. Under an idealized model, haploids carrying GFP and mScarlet would each represent 1/4 of the total post-sporulation population, and the expected proportion of GM diploids would therefore be 1/8 under random mating.

In practice, however, several factors reduce the observed proportion of GM diploids. First, diploids that fail to sporulate remain in the population and contribute to the total number of diploid cells. Second, spore lethality reduces the number of viable haploids available for mating and may do so unequally across genotypes. Third, not all viable haploids successfully mate to form diploids. As a result, the expected frequency of GM diploids is lower than 1/8 even under fully random mating.

To account for these effects, cells were plated both after sporulation and after the mating step, and the proportions of haploid and diploid colonies were measured at each stage. These measurements allowed us to estimate sporulation efficiency, the fraction of viable haploids, and mating efficiency more accurately. Combined with the observed proportion of GM diploids, these quantities were then used to infer the relative contributions of intra-tetrad and inter-tetrad mating under the genetic assumptions of the assay.

We defined the parameters as follows:

N: total initial number of the diploid cells.

k: fraction sporulated initial diploids.

v: viability of haploid spores produced by sporulation.

m: fraction of viable haploids that complete mating.

s: fraction of newly formed diploids arising from intra-tetrad mating; 1-***s*** corresponds to inter-tetrad mating.

H_1, H_2, D_1, D_2, h_1, h_2, d_1 and d_2: ***H*** and ***D*** denote the total numbers of haploids and diploids, respectively, whereas ***h*** and ***d*** denote the corresponding observed counts; subscript 1 indicates measurements taken after sporulation, and subscript 2 indicates measurements taken after mating.

The expressions for ***k*** (sporulation rate) can be derived from the first plating:

$$H_1 = 4kvN \quad (1)$$

$$D_1 = (1-k)N \quad (2)$$

At this step, the observed counts of diploids (d_1) and haploids (h_1) were recorded. Assuming that the observed ratio provides an unbiased estimate of the total ratio, that is, $d_1/h_1 \approx D_1/H_1$, the sporulation rate *k* can be derived from equations (1) and (2) as:

$$k = \frac{1}{4v\frac{d_1}{h_1} + 1} \quad (3)$$

During the second plating, H_2 and D_2 can be used to derive ***m*** (mating efficiency):

$$H_2 = 4kv(1-m)N \quad (4)$$

$$D_2 = N(1-k) + 2vkmN \quad (5)$$

From equation (4), it is clear that when $H_2 = 0$, $m = 1$.

When $H_2 > 0$, assuming $d_2/h_2 \approx D_2/H_2$, the mating efficiency ***m*** can be derived from equations (4) and (5) as:

$$m = \frac{4vk\frac{d_2}{h_2} - (1-k)}{2vk(2\frac{d_2}{h_2} + 1)} \tag{6}$$

The genotypes of diploid cells collected from the second plating were then identified, which include GO (GFP/-), MO (mScarlet/-), GG (GFP/GFP), MM (mScarlet/mScarlet), GM (GFP/mScarlet), and OO (-/-).

The non-sporulating diploids consist of the GO and MO. Under the assumption that the fluorescent locus is unlinked to the MAT locus or any lethal genotypes, their ratio should be GO:MO = 1:1. For the diploids generated by mating after sporulation, meiosis doubles the total cell number relative to the initial diploid population. Diploids formed through intra-tetrad mating include all genotypes except for GM, the ratio is expected to be GO:MO:GG:MM:OO = 4:4:1:1:2, and the ratio for inter-tetrad mating is GO:MO:GG:MM:GM:OO = 4:4:1:1:2:4. Among these, the GM genotype arises exclusively from inter-tetrad mating.

Considering the sporulation rate (***k***), spore viability (***v***), mating efficiency (***m***), and the proportion of cells undergoing inter-tetrad mating (1-***s***), the total number of GM generated through inter-tetrad mating is given by:

$$N(GM) = \frac{1}{8}kvm(1-s) \cdot 2N \tag{7}$$

Similarly, the total numbers of other genotypes are listed below:

Diploid genotype	Non-sporulated	Intra-tetrad mating	Inter-tetrad mating	Total number
GO	$\frac{1}{2}(1-k) \cdot N$	$\frac{1}{3}kvms \cdot 2N$	$\frac{1}{4}kvm(1-s) \cdot 2N$	$\frac{1}{2}(1-k) \cdot N + (\frac{1}{4}kvm + \frac{1}{12}kvms) \cdot 2N$
MO	$\frac{1}{2}(1-k) \cdot N$	$\frac{1}{3}kvms \cdot 2N$	$\frac{1}{4}kvm(1-s) \cdot 2N$	$\frac{1}{2}(1-k) \cdot N + (\frac{1}{4}kvm + \frac{1}{12}kvms) \cdot 2N$
GG	0	$\frac{1}{12}kvms \cdot 2N$	$\frac{1}{16}kvm(1-s) \cdot 2N$	$(\frac{1}{16}kvm + \frac{1}{48}kvms) \cdot 2N$
MM	0	$\frac{1}{12}kvms \cdot 2N$	$\frac{1}{16}kvm(1-s) \cdot 2N$	$(\frac{1}{16}kvm + \frac{1}{48}kvms) \cdot 2N$
GM	0	0	$\frac{1}{8}kvm(1-s) \cdot 2N$	$(\frac{1}{8}kvm - \frac{1}{8}kvms) \cdot 2N$
OO	0	$\frac{1}{6}kvms \cdot 2N$	$\frac{1}{4}kvm(1-s) \cdot 2N$	$(\frac{1}{4}kvm - \frac{1}{12}kvms) \cdot 2N$

Therefore, we can obtain the expected frequency of the GM diploids:

$$Freq(\text{GM}) = \frac{kvm(1-s)}{4(1-k+2kvm)} \tag{8}$$

The proportion of inter-tetrad mating (1-$\boldsymbol{s}$) can be determined as a function of the $\boldsymbol{k}$, $\boldsymbol{v}$, $\boldsymbol{m}$ and the frequency of GM diploids:

$$1-s = \frac{4(1-k+2kvm)}{kvm} \cdot Freq(\text{GM}) \tag{9}$$

Spore viability ($\boldsymbol{v}$) was previously measured in Fig. 2A, and the mean value from the three independent measurements ($\boldsymbol{v}$ = 0.638) was used for subsequent calculations. During the first plating, sporulation products from all GO and MO groups were plated. For each group, 24 cells were randomly selected to quantify the diploids-to-haploids ratio and $\boldsymbol{k}$ was calculated based on equation (3) (Fig. EV1D). There was no obvious difference in sporulation rate between GO and MO diploids (ANOVA, p = 0.97), so the mean value $\boldsymbol{k}$ = 0.612 was used as the estimate of the sporulation rate. This estimate was also not significantly different from the direct microscopic estimate shown in Extended Fig. 1 (ANOVA, p = 0.92).

After mating, cells were plated a second time. For each of the six replicates from the reciprocal crosses, 96 colonies were selected for mating-type identification. Most of the selected clones were diploid and the calculated mean mating efficiency was estimated to be $\boldsymbol{m}$ = 0.987 (Fig. EV1E).

Finally, among the 560 identified diploids, we determined the proportion of GM diploids and used equation (9) to calculate the estimated value 1 - $\boldsymbol{s}$ (Fig. EV1F). The results indicate that approximately 73% of newly formed diploids arose through inter-tetrad mating. Together, these measurements indicate that mating during the cross-sporulation cycle consists of both intra-tetrad and inter-tetrad mating, with inter-tetrad mating accounting for the majority of events.

Evaluating the effects of inter- and intra-tetrad mating on allele frequency

The mating process of the cross-sporulation cycle (Fig. 1A) consists of both inter-tetrad mating (random mating) and intra-tetrad mating (sister-spore mating). It is necessary to evaluate how different mating patterns affect the variation in allele frequency. In our case, the allele frequency variation is driven primarily by the lethality of VIII-A spores. Lethal genotypes are produced exclusively by Y55/DBVPG1373 hybrid diploids. To quantify the impact of mating patterns on the allele frequency dynamics, we employed an *in silico* simulation to model allele frequency trajectories across consecutive cycles of sporulation and mating.

1. Complete Inter-tetrad Mating Model

Each simulation cycle consists of two distinct stages: sporulation and mating. In sporulation stages, a subset of diploids (defined by the sporulation rate, s) undergoes meiosis. All diploids excluding the Y55/DBVPG1373 hybrids produce four viable spores with 2+2 parental genotypes. Y55/DBVPG1373 hybrids produce spores according to the three segregation patterns illustrated in Fig. 4A. In the subsequent mating stages, under a complete inter-tetrad model, all spores enter a well-mixed pool and mate randomly to form the next generation diploids. This model represents the upper bound for recombinant potential and rapid allele frequency shifts.

2. Complete Intra-tetrad Mating Model

In the intra-tetrad scenario, mating is primarily restricted to sister spores derived from the same meiotic event. The sporulation procedures remain identical to the inter-tetrad model. In mating stages, spores mate with partners of the opposite mating type within their own tetrad. In case of crossover-associated adjacent-1 segregation (3 viable spores) or adjacent-1 segregation (2 viable spores if surviving spores share identical mating type), the extra spores are permitted to mate randomly with spores from other tetrads. Critically, because intra-tetrad mating increases homozygosity and restricts the shuffling of alleles, its primary effect is to buffer the population against rapid change, thereby slowing the rate of allele frequency shift without altering the trend.

3. Simulation Implementation and Empirical Validation

To bridge these theoretical frameworks with experimental reality, we implemented a stochastic simulation using a custom Python script that tracks genotype and allele frequency trajectories across the population. Each simulation cycle begins by calculating the viable spores produced based on the sporulation rate (k = 0.6) and the specific segregation probabilities derived from our empirical tetrad dissection data (21 alternate, 4 adjacent-1, and 53 crossover-associated adjacent-1 segregation events; Fig. 4A). The mating stage was executed according to the logic of two models described above, and each simulation was run for six discrete generations to mimic the experiments in the main text. The resulting trajectories (Appendix Fig. 13) demonstrate that while intra-tetrad mating may influence the rate of allele frequency change, the fundamental drive toward increased allele frequency remains robust. These results confirm that the selective pressure from lethality of VIII-A spores is the dominant driver of the observed allele frequency dynamics.

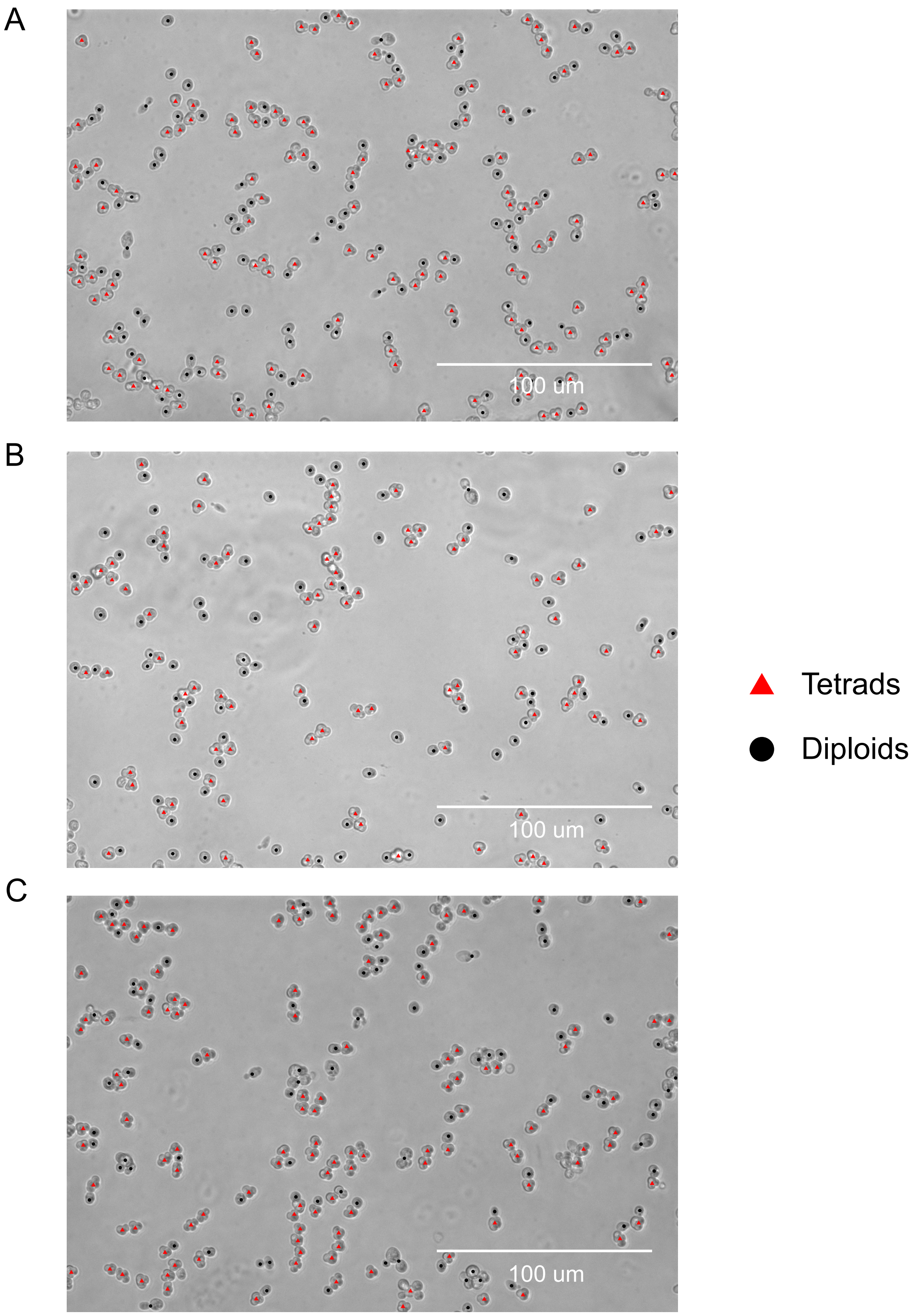

Appendix Figure S1: Assessment of sporulation efficiency by microscopic examination. Red triangles mark tetrads (four haploid spores enclosed within an ascus), and black circles mark diploid cells. Panels (A)-(C) show three biological replicates, with tetrad/total cell counts of 134/240, 91/173, and 126/215, corresponding to sporulation efficiencies of 55.8%, 52.6%, and 58.6%, respectively.

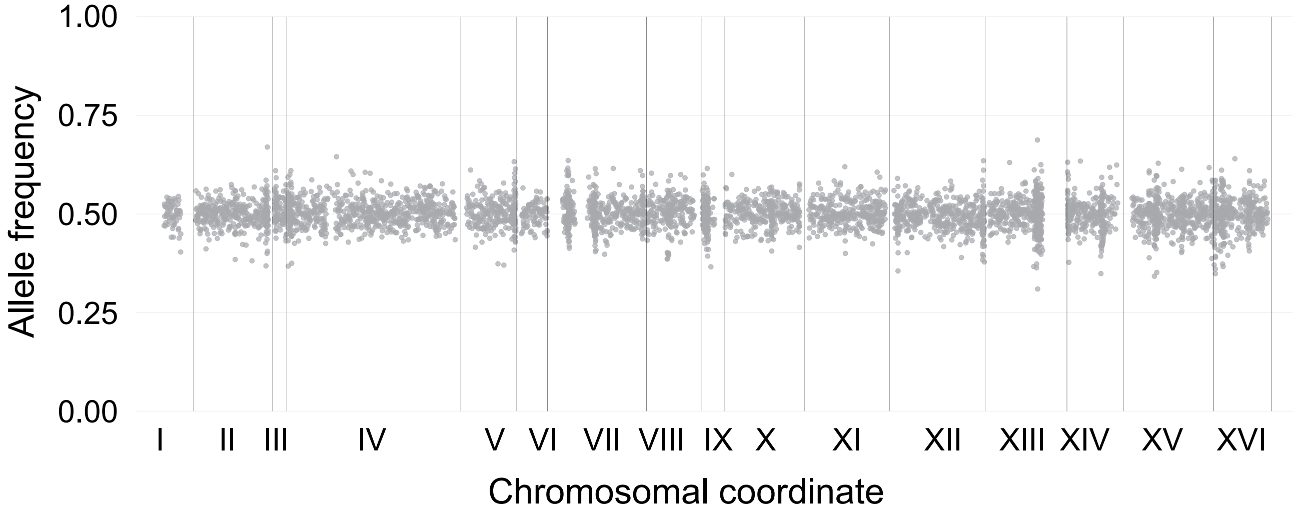

Appendix Figure S2: The allele frequency of the initial hybrids of Y55 (MATa) x DBVPG1373(MATα). The allele frequency is equally distributed at 0.5 across all chromosomes in the initial hybrids. The y-axis represents DBVPG1373 allele frequencies, which serve as the reference for subsequent analyses.

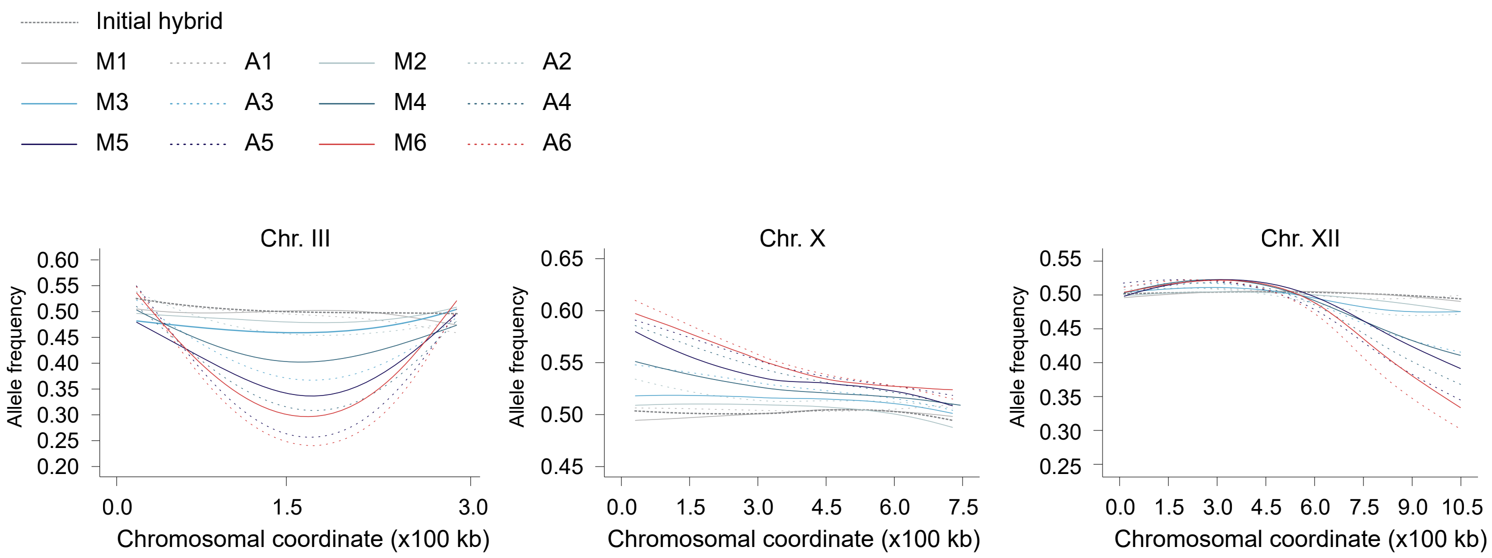

Appendix Figure S3: Allele frequency of chromosomes III, X and XII. The dotted line represents the initial hybrid, the solid lines represent the sporulation products and the dashed lines are mitotic growth products. Curves derived from meiotic products and mitotic growth products of the same round are shown in the same color.

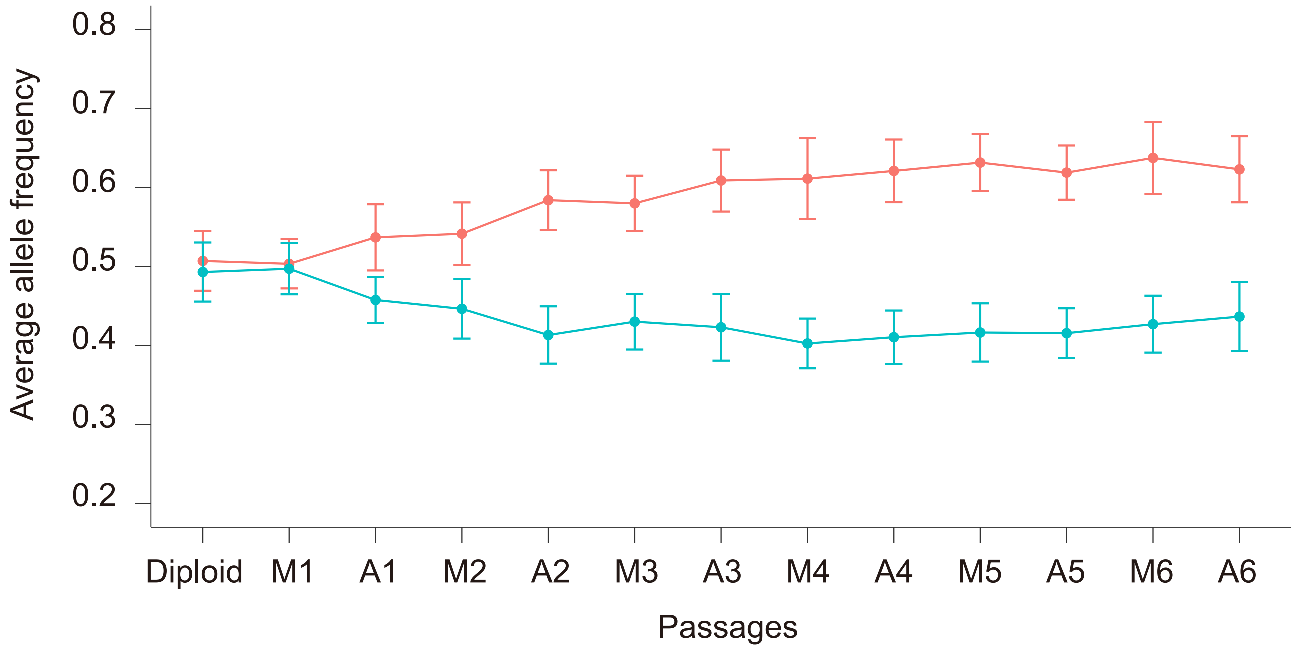

Appendix Figure S4: Average allele frequency within 50,000 bp right side of the chromosomal breakpoints. The average allele frequency was calculated using all SNPs detected in the regions spanning 14,867 – 64,867 bp on chromosome VIII and 373,654 – 423,654 bp on chromosome XVI. M_n represents the nth round of sporulation products, and A_n represents the nth round of mitotic growth products. Data are presented as mean ± SD from three independent replicates.

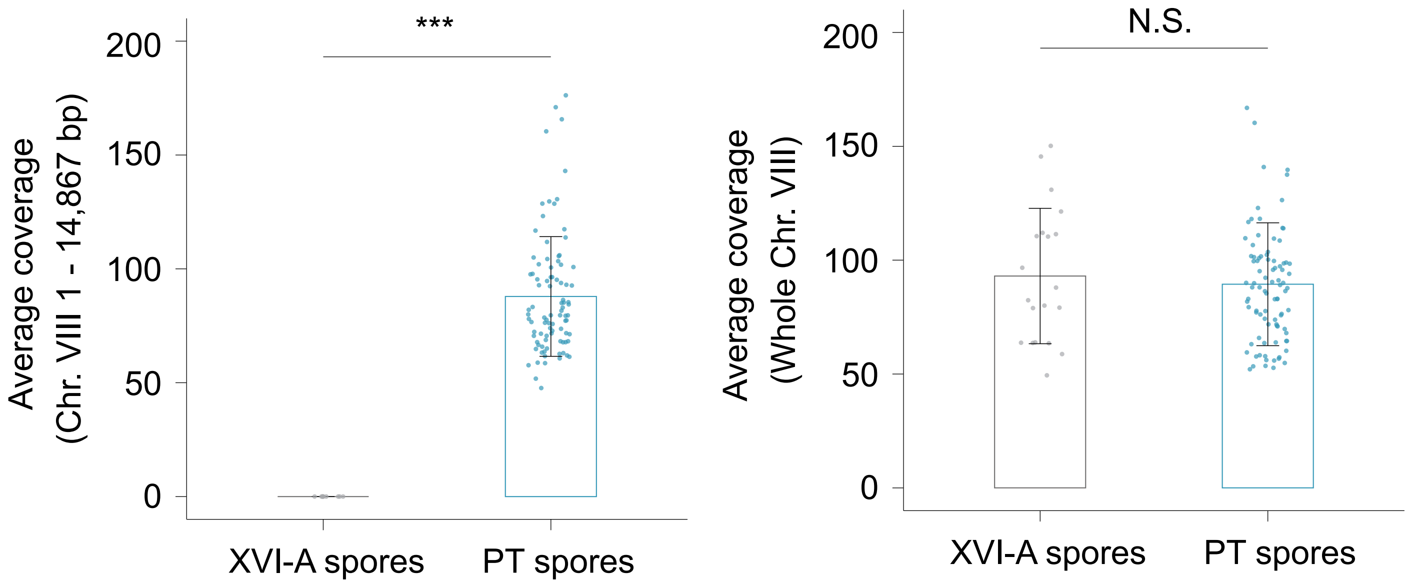

Appendix Figure S5: Average sequencing coverage of different regions on Chr.VIII. The left panel shows the average coverages of the 15 kb region at the upstream end of Chr. VIII and the right panel shows the average coverages of the entire Chr. VIII from XVI-A and PT spores. Significance between groups was examined using the Wilcoxon test, where *** represents $p < 0.001$ and N.S. indicates no significant difference.

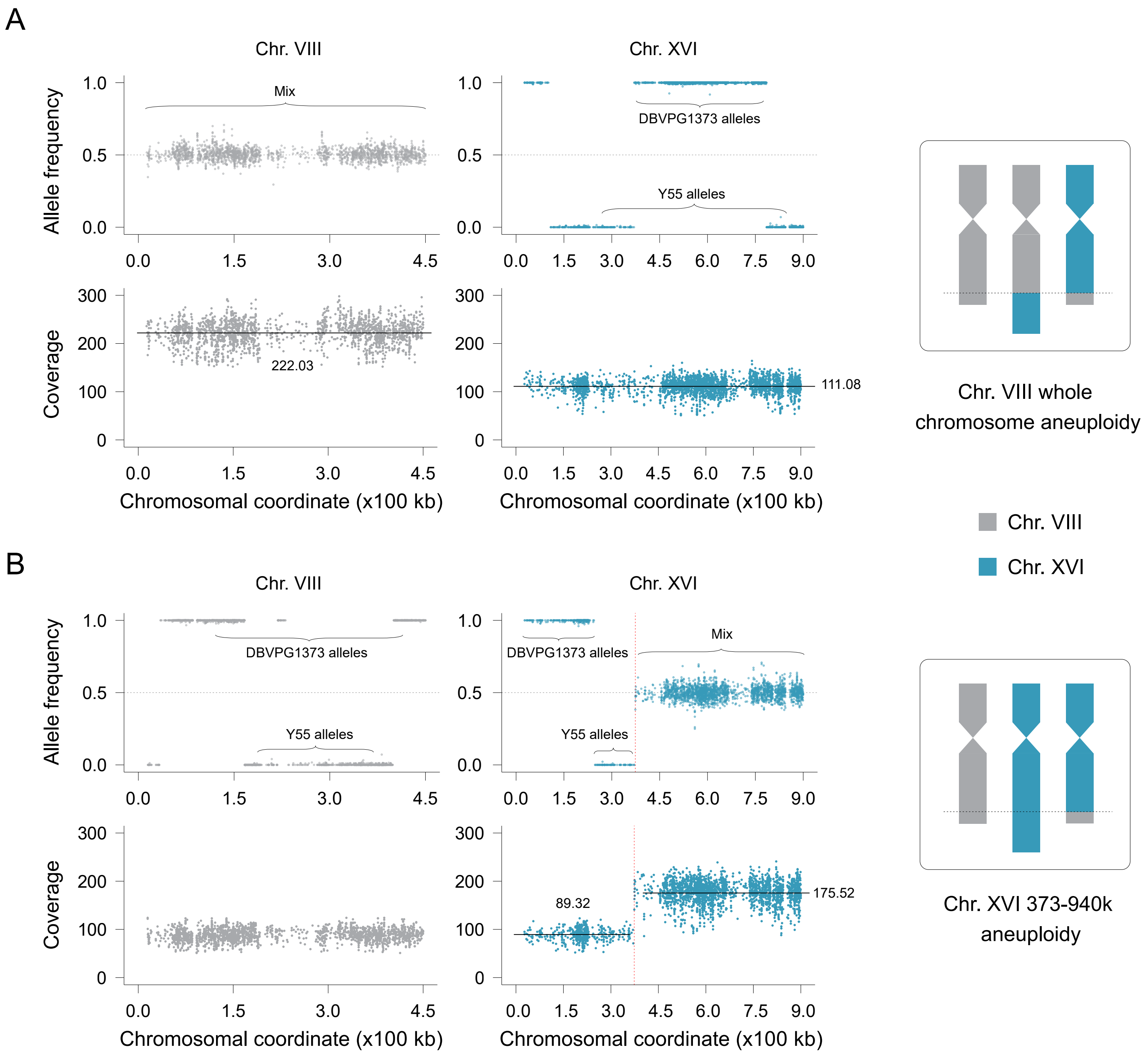

Appendix Figure S6: Allele frequencies and sequencing coverage of spores with two additional karyotypes. The upper panel shows the allele frequencies of Chr. VIII and Chr. XVI, and the lower panels show the corresponding sequencing coverage. The black solid lines on the coverage panels indicate the average coverage, with the exact value shown above. **(A)** For spores carrying whole-chromosome aneuploidy of chromosome VIII, the sequencing coverage of chromosome VIII is doubled, and the allele frequency is approximately 0.5. This indicates the simultaneous presence of Chr. VIII copies derived from both parental strains. **(B)** For spores with partial aneuploidy on 373–940 kb on Chr. XVI, a twofold increase in sequencing coverage and allele frequency approaches 0.5 can also be observed across this region, indicating the presence of two copies of Chr. XVI derived from both parents.

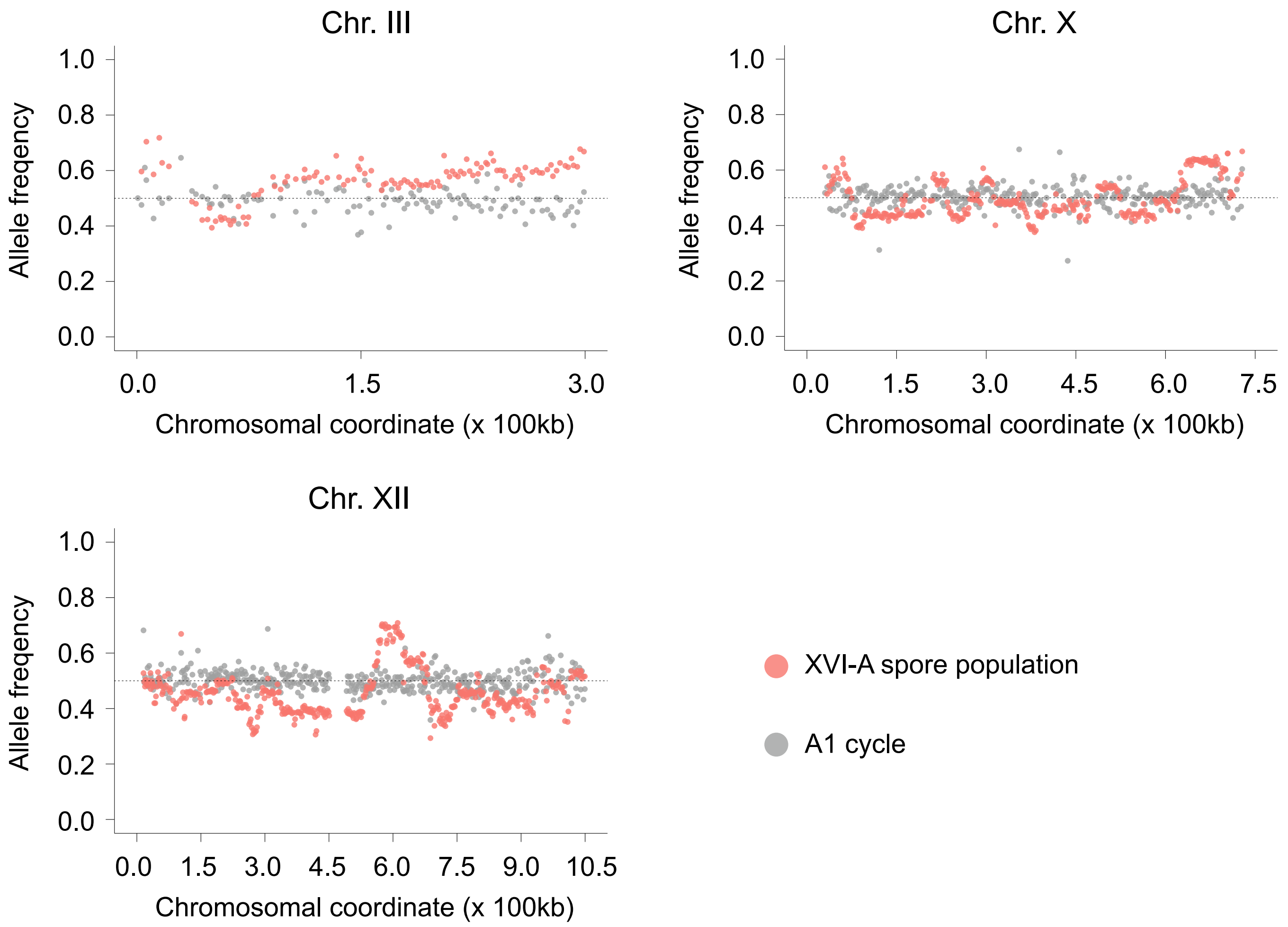

Appendix Figure S7: Allele frequency variations between XVI-A spore population and A1 cycle. The Allele frequencies of chromosomes III, X and XII from the spore population composed of randomly selected XVI-A spores are shown as red plots and the frequencies of the A1 round of mitotic growth product are represented as grey plots.

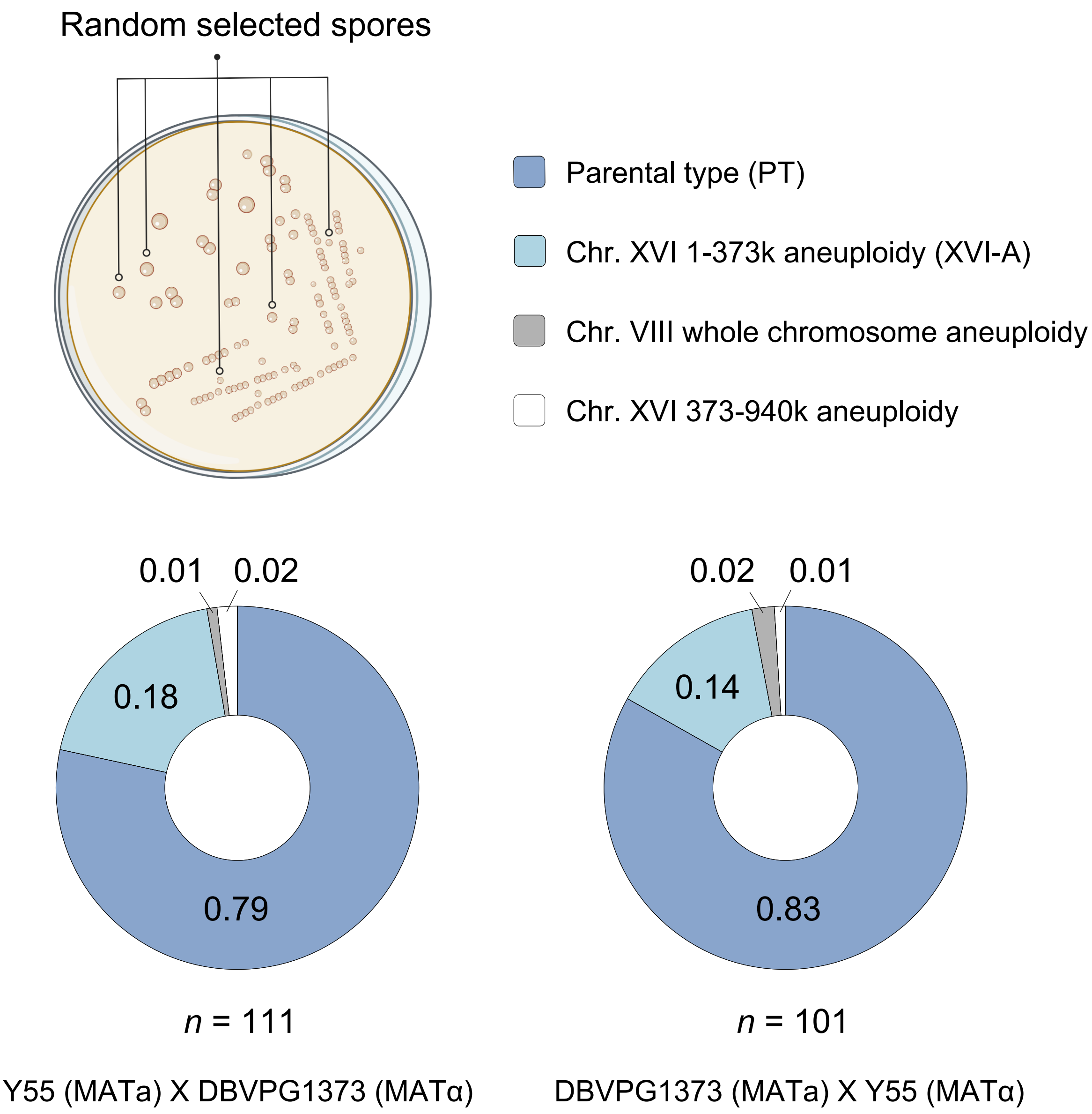

Appendix Figure S8: The proportions of different karyotypes of the spores from the reciprocal crosses between strains Y55 and DBVPG1373.

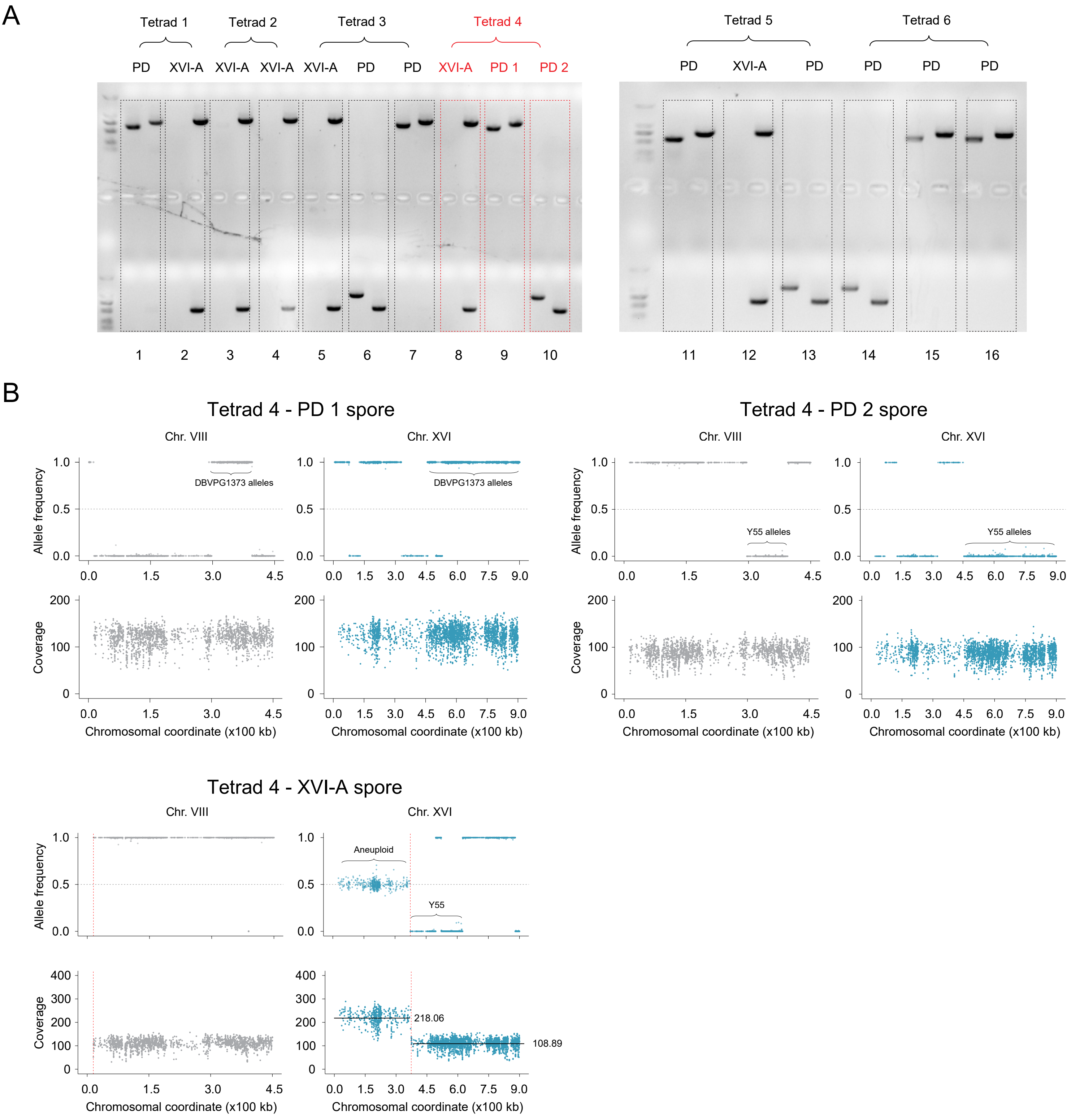

Appendix Figure S9: Validation of the PCR-based karyotype analysis method. (A) PCR results for 16 spores from 6 tetrads. Dashed squares indicate the PCR result for each spore, with the identified karyotype shown above the squares. **(B)** WGS results of three selected spores, which are highlighted with red dashed squares in panel (A). The karyotypes determined by WGS are consistent with those identified by PCR, demonstrating the reliability of the PCR-based karyotype analysis method.

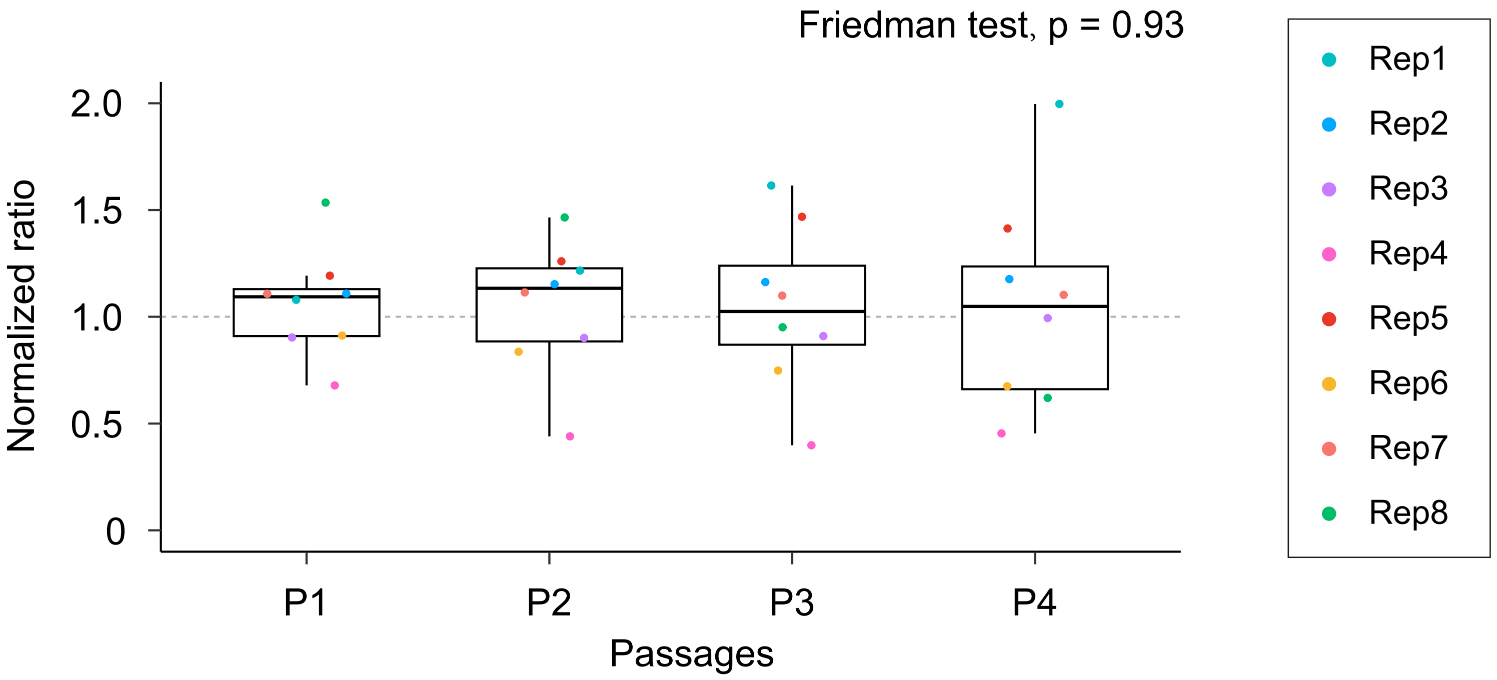

Appendix Figure S10: Pair-wise competition experiments indicate no significant fitness difference between the XVI-A and PT genotypes. The pair-wise competition experiment contained eight biological replicates, with each replicate consisting of a pair of XVI-A and PT yeast. The ratio of the two genotypes during competitive growth was determined based on their SNP frequencies. The SNP frequency of XVI-A was used as reference. The initial passage served as baseline, and the allele frequencies at subsequent passages (P1–P4) were divided by the corresponding initial frequencies to calculate normalized ratios. Boxplots show the median and interquartile range, with whiskers extending to 1.5×IQR, and each replicate is represented by jittered colored points. To assess whether normalized ratios differed across passages, an omnibus Friedman rank sum test was performed. No significant difference was detected among passages (Friedman $\chi^2 = 0.45$, df = 3, $p = 0.93$), indicating that competitive fitness remained stable across passages and there is no overall fitness difference between the XVI-A and PT strains under competitive growth.

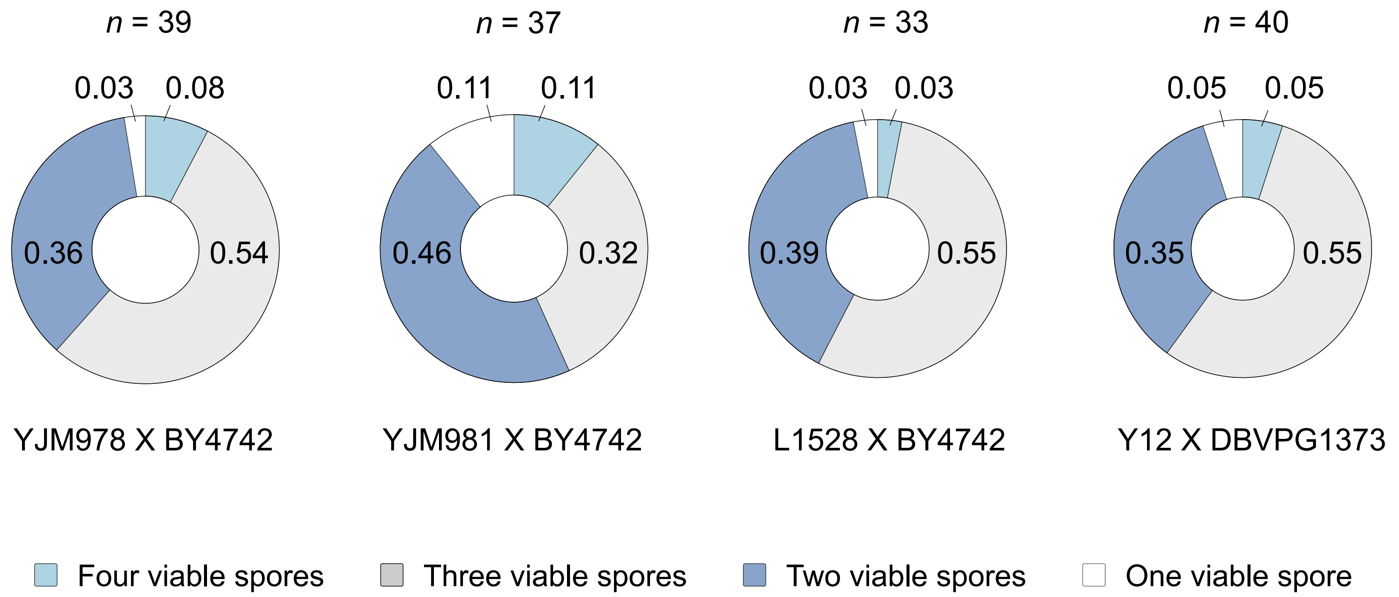

Appendix Figure S11: Proportions of viable spores from each tetrad of different parental strains.

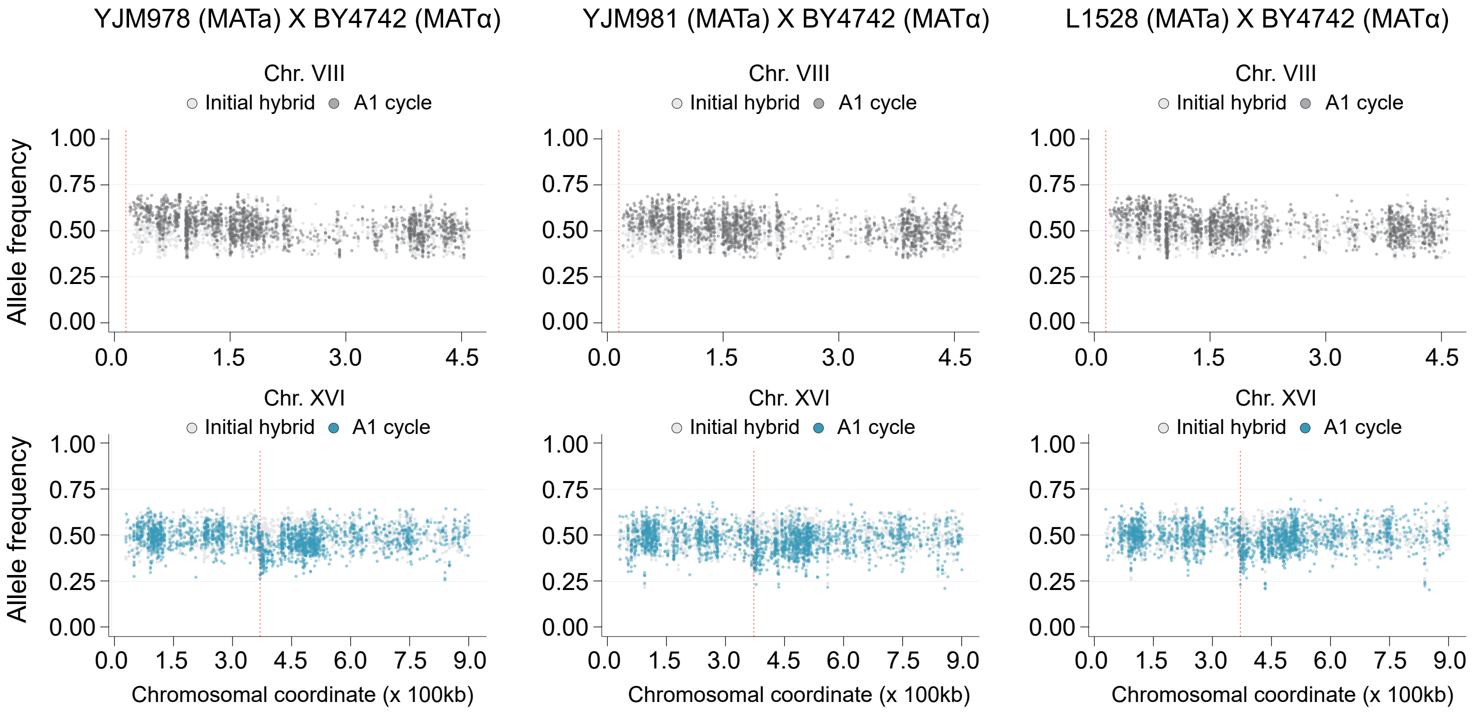

Appendix Figure S12: Allele frequency variations between initial hybrids and the A1 cycle. Three hybrid combinations, including YJM978 (MATa) × BY4742 (MATα), YJM981 (MATa) × BY4742 (MATα), and L1528 (MATa) × BY4742 (MATα), were independently subjected to cross-sporulation cycles. Allele frequencies of Chr. VIII and Chr. XVI were compared between the initial hybrids and the A1 cycle. The allele frequencies in the initial hybrids are shown as gray dots, Chr. VIII frequencies as dark black dots, and A1 cycle frequencies as blue dots. The frequency changes of these two chromosomes follow the same trend observed in the cross-sporulation experiment of Y55 × DBVPG1373.

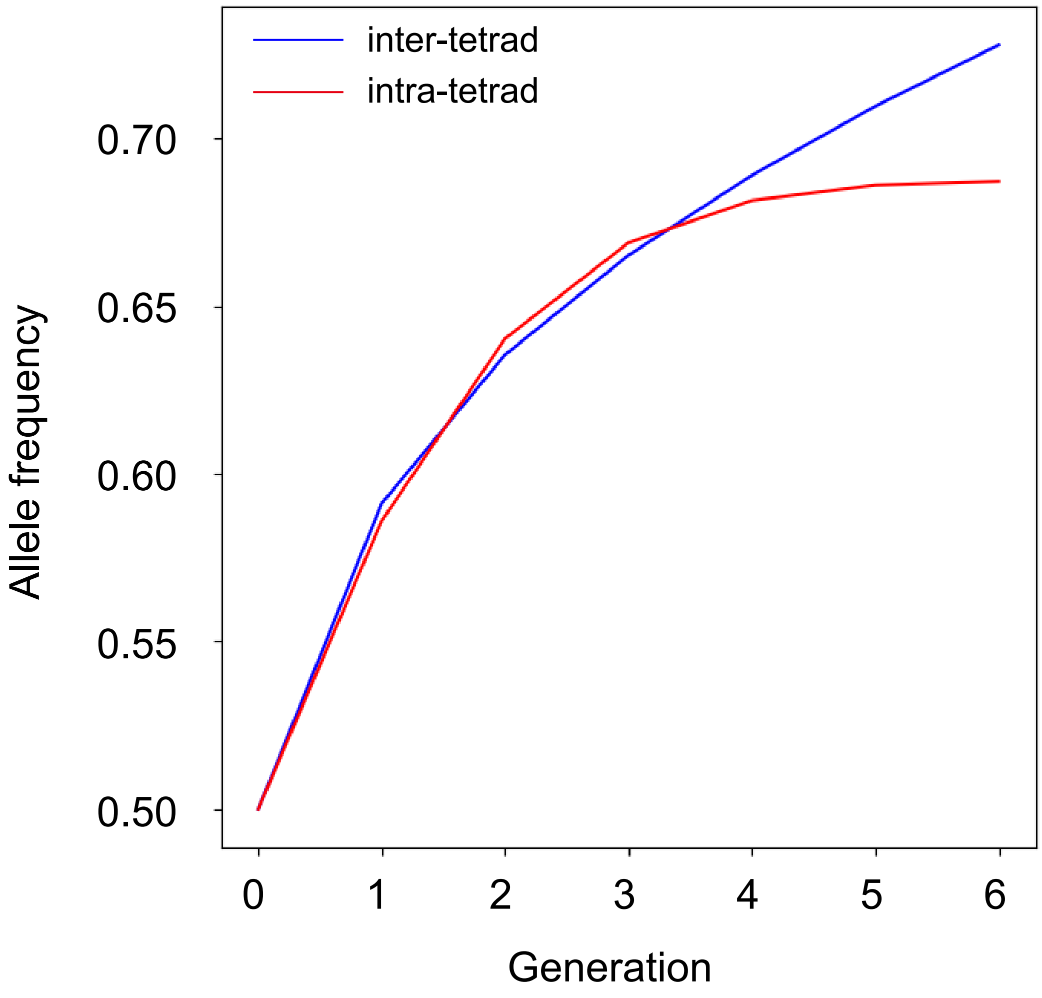

Appendix Figure S13: In silico simulations of allele-frequency shifts across consecutive cycles of sporulation and mating under different mating modes.
