# Supplementary material for: Rapid evolution driven by translocation-associated selection during meiosis
Source: EMBO Rep. 2026 Jun 16;27(14):4011–28. doi: 10.1038/s44319-026-00820-6 (PMC13400751; doi:10.1038/s44319-026-00820-6)
Supplement: Supplementary file 12 — Source data Fig. 1 [file 44319_2026_820_MOESM12_ESM.zip › Figure 1 Source Data/1B/Readme.docx]

Figure 1B was generated using data from three biological replicates of the initial diploid population and the sixth cross-sporulation cycle population from the Y55 (MATa) x DBVPG1373 (MATalpha) cross. In the header row, the prefix “AF” denotes allele frequency, “DP” denotes sequencing depth, “Initial” refers to the initial population, and “Sixth_round” refers to the sixth-round population of the cross-sporulation cycle. AF and Position represent the median values within each 2,000-bp window, whereas DP represents the mean sequencing depth within each window. The p value corresponds to the median value from the three pairwise comparisons (see *Method: Allele frequency analysis*).
