# Supplementary material for: Rapid evolution driven by translocation-associated selection during meiosis
Source: EMBO Rep. 2026 Jun 16;27(14):4011–28. doi: 10.1038/s44319-026-00820-6 (PMC13400751; doi:10.1038/s44319-026-00820-6)
Supplement: Supplementary file 13 — Source data Fig. 2 [file 44319_2026_820_MOESM13_ESM.zip › Figure 2 Source Data/2B/Readme.docx]

The tetrad dissection image shown in Figure 2B was cropped, rotated, and the contrast was adjusted with ImageJ. Both the original image and the processed image are provided.
