# Supplementary figures and images for: Rapid evolution driven by translocation-associated selection during meiosis
Source: EMBO Rep. 2026 Jun 16;27(14):4011–28. doi: 10.1038/s44319-026-00820-6 (PMC13400751; doi:10.1038/s44319-026-00820-6)

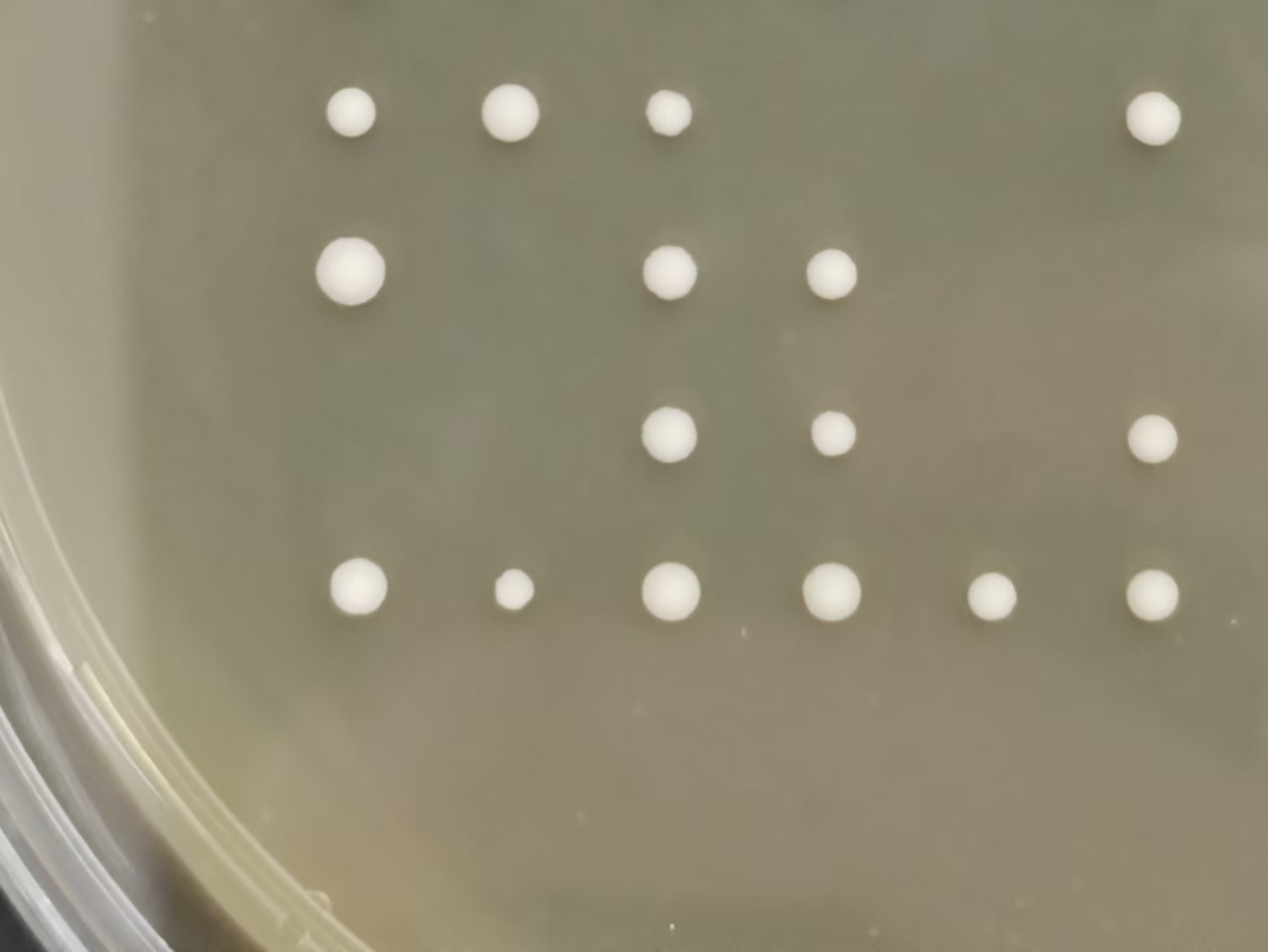

Supplement: Supplementary file 13 — Source data Fig. 2 [file 44319_2026_820_MOESM13_ESM.zip › Figure 2 Source Data/2B/Figure 2B Tetrad_Dissection_pic_original.jpg]

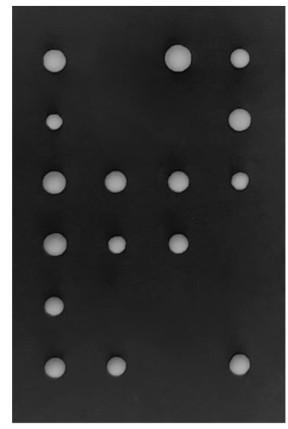

Supplement: Supplementary file 13 — Source data Fig. 2 [file 44319_2026_820_MOESM13_ESM.zip › Figure 2 Source Data/2B/Figure 2B Tetrad_Dissection_pic_processed.jpg]

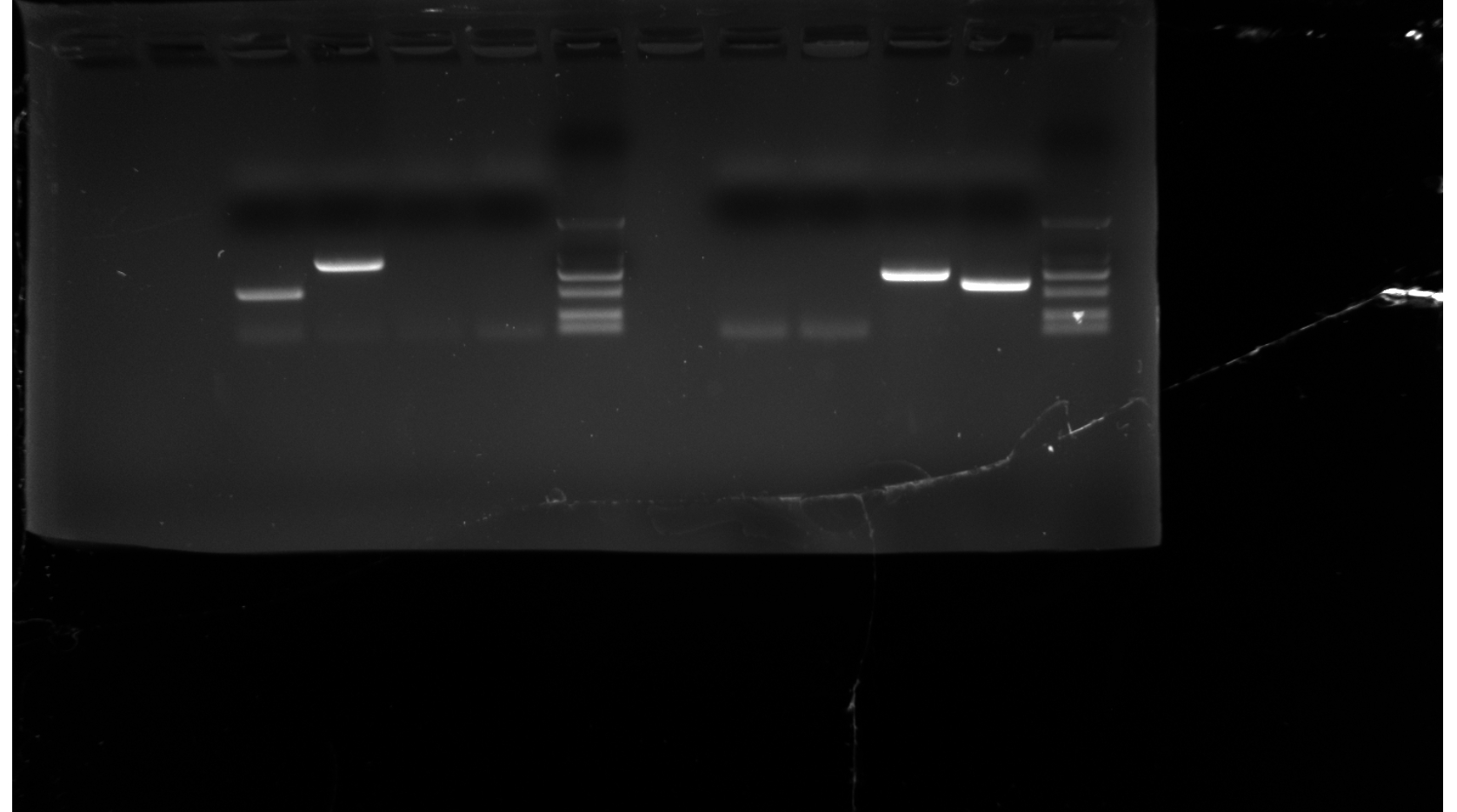

Supplement: Supplementary file 17 — Figure EV4 Source Data [file 44319_2026_820_MOESM17_ESM.zip › Figure EV4 Source Data/EV 4B PT spores (Y55 and DBVPG1373).jpg]

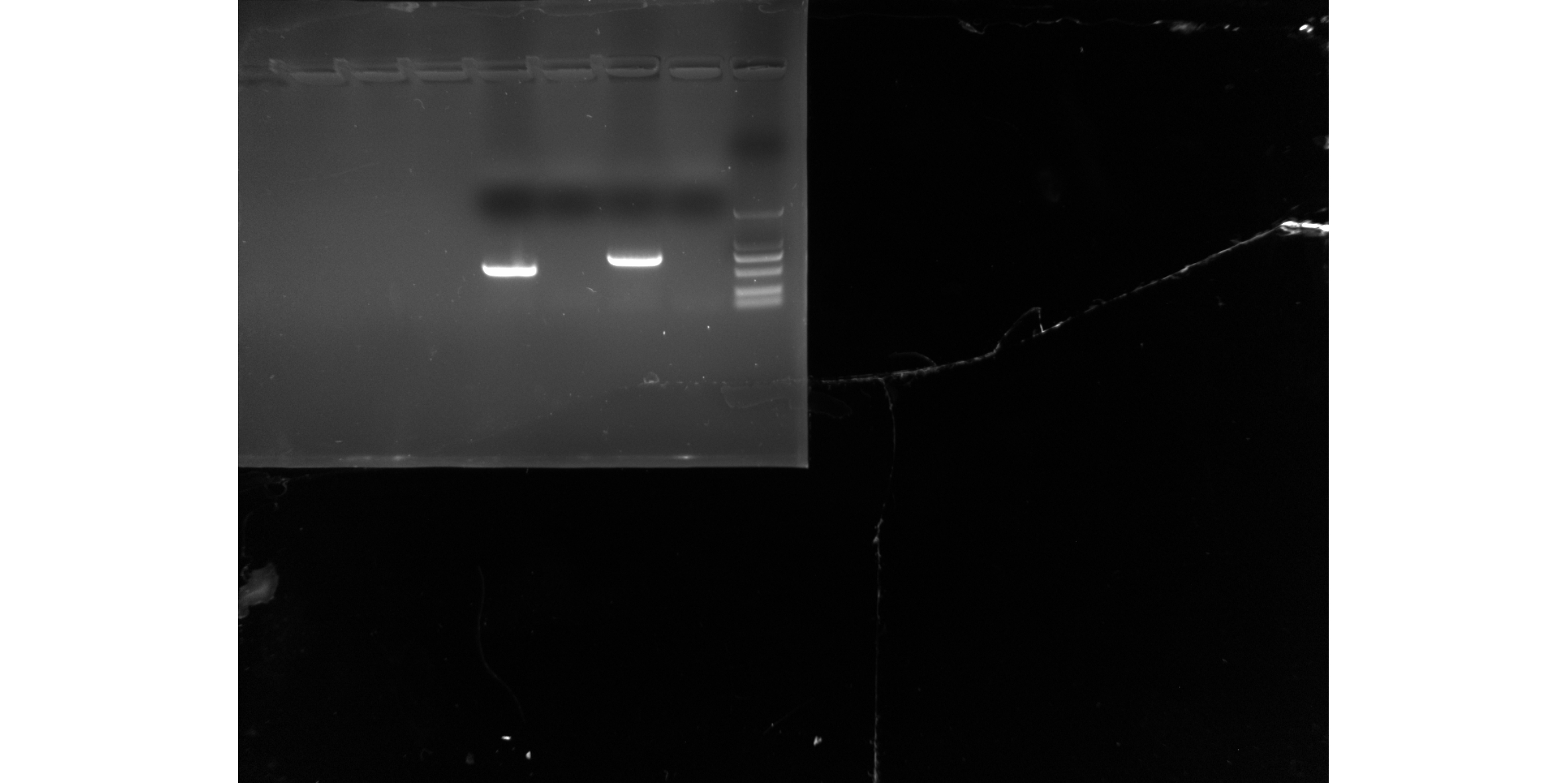

Supplement: Supplementary file 17 — Figure EV4 Source Data [file 44319_2026_820_MOESM17_ESM.zip › Figure EV4 Source Data/EV 4B XVI-A spore.jpg]
