# Supplementary material for: Rapid evolution driven by translocation-associated selection during meiosis
Source: EMBO Rep. 2026 Jun 16;27(14):4011–28. doi: 10.1038/s44319-026-00820-6 (PMC13400751; doi:10.1038/s44319-026-00820-6)
Supplement: Supplementary file 14 — Source data Fig. 3 [file 44319_2026_820_MOESM14_ESM.zip › Figure 3 Source Data/3A/README.docx]

Delta file produced by MUMmer3. Format definition see [MUMmer3 docs](https://github.com/marbl/MUMmer3/blob/4dc82cf6941043dec2c6880544a3811344ae5f4e/docs/nucmer.README#L181).

https://github.com/marbl/MUMmer3/blob/4dc82cf6941043dec2c6880544a3811344ae5f4e/docs/nucmer.README#L181
