# Supplementary material for: Rapid evolution driven by translocation-associated selection during meiosis
Source: EMBO Rep. 2026 Jun 16;27(14):4011–28. doi: 10.1038/s44319-026-00820-6 (PMC13400751; doi:10.1038/s44319-026-00820-6)
Supplement: Supplementary file 17 — Figure EV4 Source Data [file 44319_2026_820_MOESM17_ESM.zip › Figure EV4 Source Data/Readme.docx]

The gel image shown in EV 4B was cropped, assembled and the contrast was adjusted with ImageJ for better visualization. The original images are provided.
