# Supplementary material for: Rapid evolution driven by translocation-associated selection during meiosis
Source: EMBO Rep. 2026 Jun 16;27(14):4011–28. doi: 10.1038/s44319-026-00820-6 (PMC13400751; doi:10.1038/s44319-026-00820-6)
Supplement: Supplementary file 18 — Expanded View Figures [file 44319_2026_820_MOESM18_ESM.pdf]

Expanded View Figures

Figure EV1. Quantitative analysis of intra- and inter- tetrad mating of spores.

(**A**) Schematic illustration of the experiment. GO (GFP/-) and MO (mScarlet/-) strains were sporulated separately and then mixed at equal proportions in rich medium to allow vegetative growth and mating. Cell populations after sporulation and after mating were plated to determine the numbers of haploids and diploids, as well as the proportion of GM (GFP/mScarlet) cells, which were used to estimate the relative proportions of intra- and inter-tetrad mating. *k* sporulation rate, *m* mating efficiency, *s* proportion of intra-tetrad mating, *d1/h1* and *d2/h2* observed diploid-to-haploid ratios at the corresponding stages. Genotype: GG, GFP/GFP; MM, mScarlet/mScarlet; OO, $-/-$. (**B**) Representative microscopy images of cells after sporulation (before vegetative growth). Bright-field images are shown on the left, and the merged GFP and mScarlet fluorescence images are shown on the right. Representative cells marked by white boxes are magnified besides. (**C**) Representative microscopy images of cells after mating. Bright-field images are shown on the left, and merged GFP and mScarlet fluorescence images are shown on the right. Representative cells marked by white boxes are magnified besides. (**D**) Sporulation rates of the four different initial diploid genotypes. Data were shown as mean ± SD from three biological replicates per group ($n = 3$ biological replicates). No significant difference among groups was detected by one-way ANOVA ($p = 0.97$). (**E**) Mating efficiency of the reciprocal crosses. Data are shown as mean ± SD from three biological replicates per group ($n = 3$ biological replicates). No significant difference was detected by a two-sided Student's *t*-test ($p = 0.66$). (**F**) Proportion of inter-tetrad mating of the reciprocal crosses. Data were shown as mean ± SD from three biological replicates per group ($n = 3$ biological replicates). No significant difference was detected by a two-sided Student's *t*-test ($p = 0.99$).

© The Author(s)

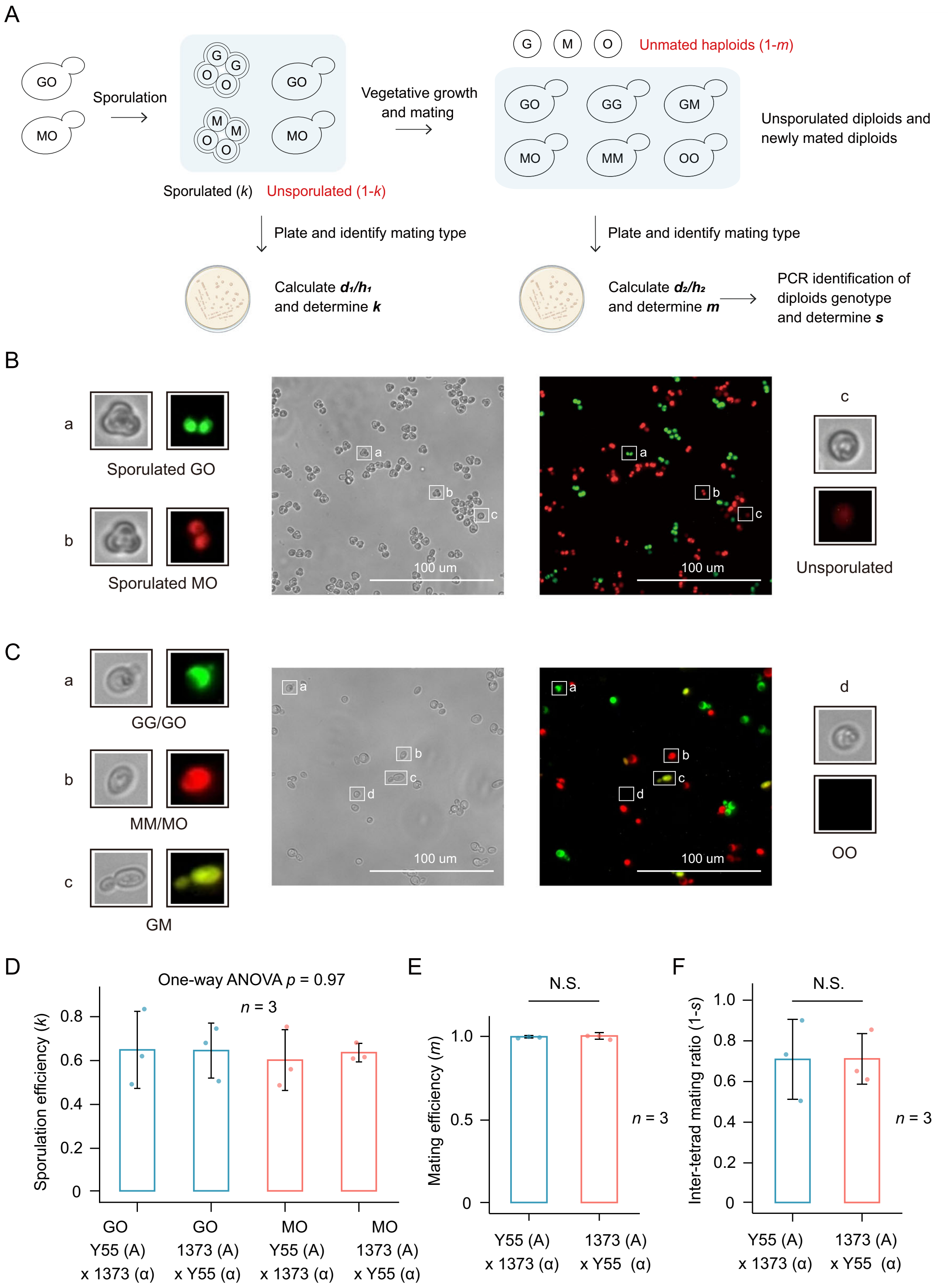
A
GO
MO
Sporulation
G G O O
M M O O
GO
MO
Sporulated (k) Unsporulated (1-k)
Vegetative growth and mating
G M O Unmated haploids (1-m)
GO GG GM
MO MM OO
Unsporulated diploids and newly mated diploids
Plate and identify mating type
Calculate d1/h1 and determine k
Plate and identify mating type
Calculate d2/h2 and determine m
PCR identification of diploids genotype and determine s
B
a
Sporulated GO
b
Sporulated MO
c
Unsporulated
100 um
C
a
GG/GO
b
MM/MO
c
GM
d
OO
100 um
D
One-way ANOVA p = 0.97
n = 3
Sporulation efficiency (k)
0 0.2 0.4 0.6 0.8
GO Y55 (A) x 1373 (α)
GO 1373 (A) x Y55 (α)
MO Y55 (A) x 1373 (α)
MO 1373 (A) x Y55 (α)
E
N.S.
n = 3
Mating efficiency (m)
0 0.5 1.0
Y55 (A) x 1373 (α)
1373 (A) x Y55 (α)
F
N.S.
n = 3
Inter-tetrad mating ratio (1-s)
0 0.2 0.4 0.6 0.8 1.0
Y55 (A) x 1373 (α)
1373 (A) x Y55 (α)

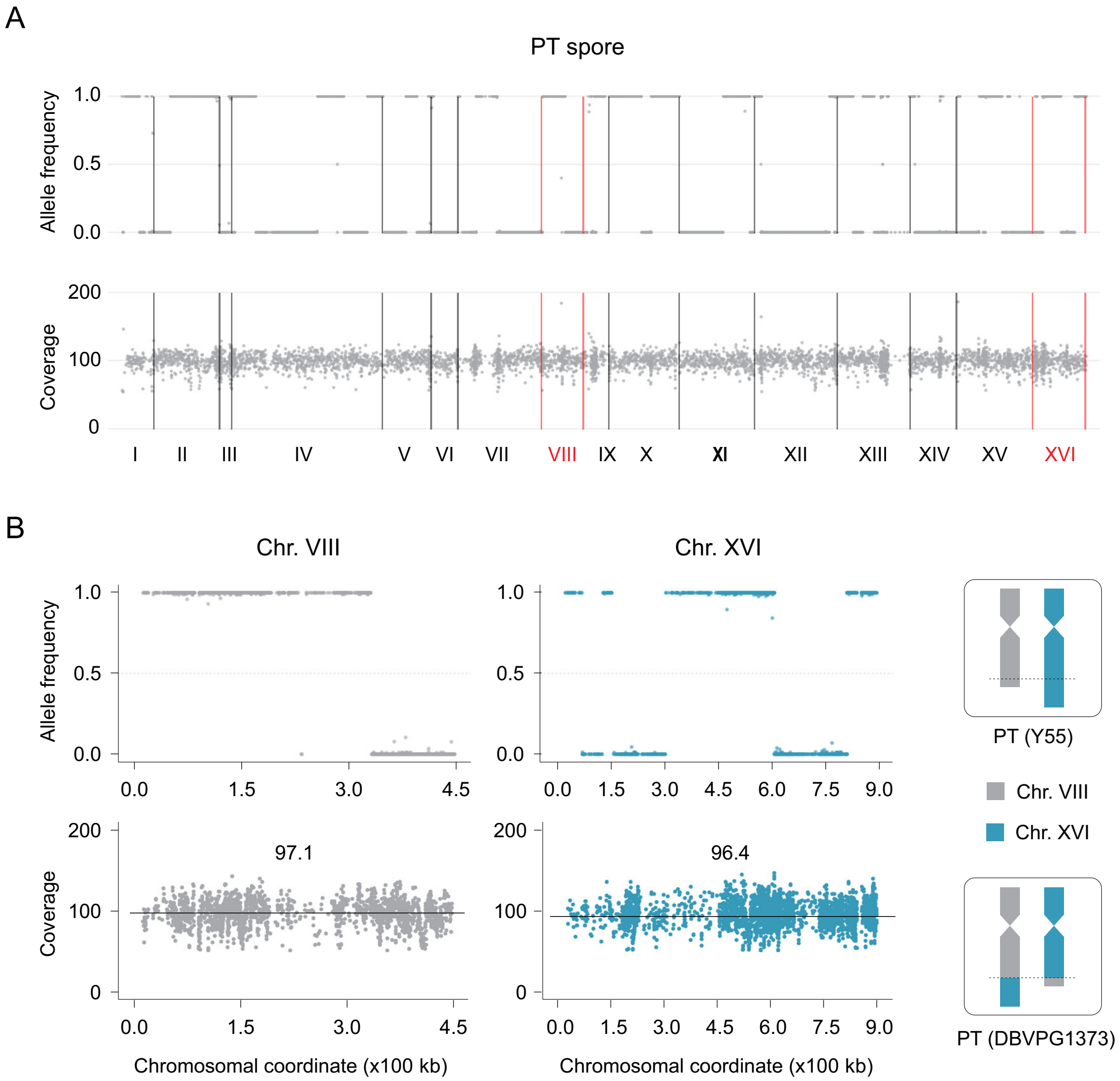

Figure EV2. Allele frequencies and sequencing coverage of SNP markers in PT spores.

(**A**) The upper panel shows the genome-wide distribution of allele frequencies, and the lower panel shows the corresponding sequencing coverage of a representative PT spore. (**B**) Enlarged views of Chr. VIII and Chr. XVI. The upper panel shows the allele frequencies along Chr. VIII and Chr. XVI, and the lower panels show the corresponding sequencing coverage. The black solid lines on the coverage panels indicate the average coverage, with the exact value shown above. In PT spores, the allele at each genomic position is expected to originate from either Y55 or DBVPG1373, and the sequencing coverage is uniform across the genome.

 © The Author(s)

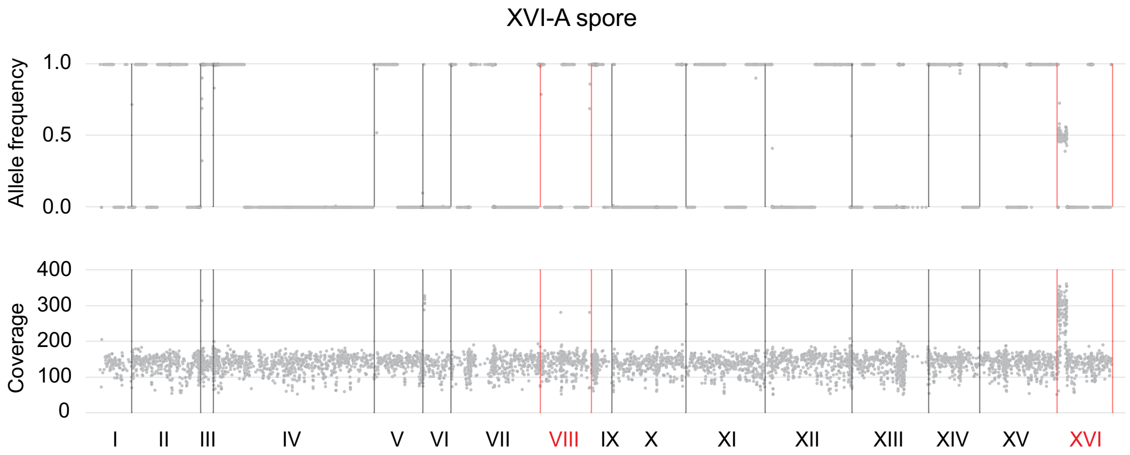

Figure EV3. Allele frequencies and sequencing coverage of SNP markers in XVI-A spores.

The upper panel shows the genome-wide distribution of allele frequencies, and the lower panel shows the corresponding sequencing coverage of a representative XVI-A spore.

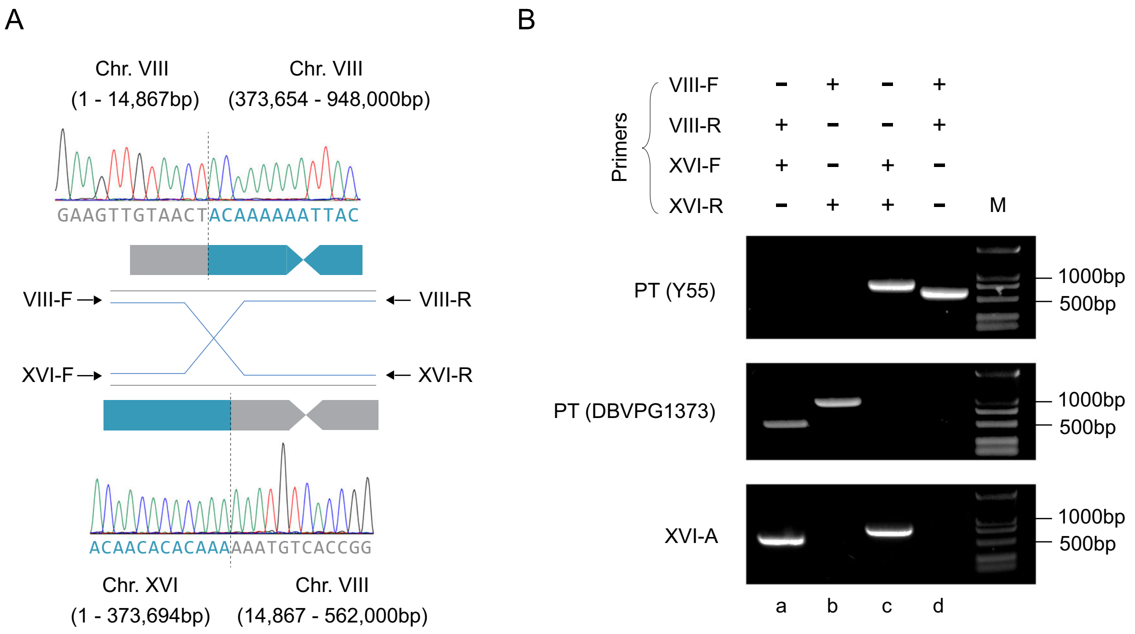

Figure EV4. Schematic of the PCR-based karyotype analysis method.

(**A**) To determine the chromosomal combinations of spores, four primers (VIII-F, VIII-R, XVI-F, and XVI-R) were used to amplify regions flanking the chromosomal breakpoints on Chr. VIII and Chr. XVI. The dashed lines indicate the position of chromosomal breakpoints and the Sanger sequencing results of these regions are shown on the figure. (**B**) The PCR result of different primer sets. If only VIII-F + VIII-R and XVI-F + XVI-R or VIII-F + XVI-R and XVI-F + VIII-R amplified successfully, the karyotype should be PT (Y55 or DBVPG1373). If only XVI-F + XVI-R and XVI-F + VIII-R amplified successfully, the karyotype is XVI-A. The PCR marker is DS2000, and the 500 and 1000 bp bands are labeled. Source data are available online for this figure.

© The Author(s)
