# Supplementary material for: Rapid evolution driven by translocation-associated selection during meiosis
Source: EMBO Rep. 2026 Jun 16;27(14):4011–28. doi: 10.1038/s44319-026-00820-6 (PMC13400751; doi:10.1038/s44319-026-00820-6)
Supplement: Supplementary file 2 — Peer Review File [file 44319_2026_820_MOESM2_ESM.pdf]

EMBO reports

Rapid evolution driven by translocation-associated selection during meiosis

Xuming Zeng, Mengdong Zhang, Xuanxuan Liu, Xionglei He, and Li Liu

Corresponding author(s): Li Liu (liul47@mail.sysu.edu.cn) , Xionglei He (hexiongl@mail.sysu.edu.cn), Li Liu (liul47@mail.sysu.edu.cn)

Review Timeline:

Submission Date:	9th Sep 25
Editorial Decision:	6th Oct 25
Revision Received:	22nd Jan 26
Editorial Decision:	18th Mar 26
Revision Received:	29th Apr 26
Editorial Decision:	7th May 26
Revision Received:	10th May 26
Accepted:	22nd May 26

Editor: Yehu Moran

Transaction Report:

(Note: With the exception of the correction of typographical or spelling errors that could be a source of ambiguity, letters and reports are not edited. Depending on transfer agreements, referee reports obtained elsewhere may or may not be included in this compilation. Referee reports are anonymous unless the Referee chooses to sign their reports.)

Dear Dr. He

Thank you for the submission of your manuscript to EMBO Reports. We have now received the full set of referee reports that are pasted below.

As you will see, the referees acknowledge that the findings are potentially interesting. However, they also raise significant comments and concerns that require your attention.

I would thus like to invite you to revise your manuscript with the understanding that the referee concerns must be fully addressed and their suggestions taken on board. Please address all referee concerns in a complete point-by-point response. Acceptance of the manuscript will depend on a positive outcome of a second round of review. It is EMBO reports policy to allow a single round of major revision only and acceptance or rejection of the manuscript will therefore depend on the completeness of your responses included in the next, final version of the manuscript.

We realize that it is difficult to revise to a specific deadline. In the interest of protecting the conceptual advance provided by the work, we recommend a revision within 3 months (6th Jan 2026). Please discuss the revision progress ahead of this time with the editor if you require more time to complete the revisions.

You can either publish the study as a short report or as a full article. For short reports, the revised manuscript should not exceed 27,000 characters (including spaces but excluding materials & methods and references) and 5 main plus 5 expanded view figures. The results and discussion sections must further be combined, which will help to shorten the manuscript text by eliminating some redundancy that is inevitable when discussing the same experiments twice. For a normal article there are no length limitations, but it should have more than 5 main figures and the results and discussion sections must be separate. In both cases, the entire materials and methods must be included in the main manuscript file.

IMPORTANT NOTE: we perform an initial quality control of all revised manuscripts before re-review. Your manuscript will FAIL this control and the handling will be DELAYED if the following APPLIES:
1) A data availability section providing access to data deposited in public databases is missing. If you have not deposited any data, please add a sentence to the data availability section that explains that.
2) Your manuscript contains statistics and error bars based on n=2. Please use scatter blots in these cases. No statistics should be calculated if n=2.

When submitting your revised manuscript, please carefully review the instructions that follow below. Failure to include requested items will delay the evaluation of your revision.

1) a .docx formatted version of the manuscript text (including legends for main figures, EV figures and tables). Please make sure that the changes are highlighted to be clearly visible.

2) individual production quality figure files as .eps, .tif, .jpg (one file per figure). See https://wol-prod-cdn.literatumonline.com/pb-assets/embo-site/EMBOPress_Figure_Guidelines_061115-1561436025777.pdf for more info on how to prepare your figures.

3) We replaced Supplementary Information with Expanded View (EV) Figures and Tables that are collapsible/expandable online. A maximum of 5 EV Figures can be typeset. EV Figures should be cited as 'Figure EV1, Figure EV2" etc... in the text and their respective legends should be included in the main text after the legends of regular figures.

- For the figures that you do NOT wish to display as Expanded View figures, they should be bundled together with their legends in a single PDF file called *Appendix*, which should start with a short Table of Content. Appendix figures should be referred to in the main text as: "Appendix Figure S1, Appendix Figure S2" etc. See detailed instructions regarding expanded view here: <https://www.embopress.org/page/journal/14693178/authorguide#expandedview>

- Additional Tables/Datasets should be labeled and referred to as Table EV1, Dataset EV1, etc. Legends have to be provided in a separate tab in case of .xls files. Alternatively, the legend can be supplied as a separate text file (README) and zipped together with the Table/Dataset file.

4) a .docx formatted letter INCLUDING the reviewers' reports and your detailed point-by-point responses to their comments. As part of the EMBO Press transparent editorial process, the point-by-point response is part of the Review Process File (RPF), which will be published alongside your paper.

5) a complete author checklist, which you can download from our author guidelines <https://www.embopress.org/page/journal/14693178/authorguide>. Please insert information in the checklist that is also reflected in the manuscript. The completed author checklist will also be part of the RPF.

6) Please note that all corresponding authors are required to supply an ORCID ID for their name upon submission of a revised manuscript (<https://orcid.org/>). Please find instructions on how to link your ORCID ID to your account in our manuscript tracking system in our Author guidelines <https://www.embopress.org/page/journal/14693178/authorguide#authorshipguidelines>

7) Before submitting your revision, primary datasets produced in this study need to be deposited in an appropriate public database (see https://www.embopress.org/page/journal/14693178/authorguide#datadeposition). Please remember to provide a reviewer password if the datasets are not yet public. The accession numbers and database should be listed in a formal "Data Availability" section placed after Materials & Method (see also https://www.embopress.org/page/journal/14693178/authorguide#datadeposition). Please note that the Data Availability Section is restricted to new primary data that are part of this study. * Note - All links should resolve to a page where the data can be accessed. *
If your study has not produced novel datasets, please mention this fact in the Data Availability Section.

8) At EMBO Press we ask authors to provide source data for the main manuscript figures. You will receive a separate email with instructions for providing source data with your revised manuscript, including how to upload and organize the files.

9) Our journal also encourages inclusion of *data citations in the reference list* to directly cite datasets that were re-used and obtained from public databases. Data citations in the article text are distinct from normal bibliographical citations and should directly link to the database records from which the data can be accessed. In the main text, data citations are formatted as follows: "Data ref: Smith et al, 2001" or "Data ref: NCBI Sequence Read Archive PRJNA342805, 2017". In the Reference list, data citations must be labeled with "[DATASET]". A data reference must provide the database name, accession number/identifiers and a resolvable link to the landing page from which the data can be accessed at the end of the reference. Further instructions are available at https://www.embopress.org/page/journal/14693178/authorguide#referencesformat

10) Regarding data quantification (see Figure Legends: https://www.embopress.org/page/journal/14693178/authorguide#figureformat)

The following points must be specified in each figure legend:

- the name of the statistical test used to generate error bars and P values,

- the number (n) of independent experiments (please specify technical or biological replicates) underlying each data point,

- the nature of the bars and error bars (s.d., s.e.m.),

- If the data are obtained from n Program fragment delivered error ``Can't locate object method "less" via package "than" (perhaps you forgot to load "than"?) at //ejpvfs23/sites23b/embor_www/letters/embor_decision_revise_and_review.txt line 56.' 2, use scatter blots showing the individual data points.

Discussion of statistical methodology can be reported in the materials and methods section, but figure legends should contain a basic description of n, P and the test applied.

- Please also include scale bars in all microscopy images.

11) The journal requires a statement specifying whether or not authors have competing interests (defined as all potential or actual interests that could be perceived to influence the presentation or interpretation of an article). In case of competing interests, this must be specified in your disclosure statement. Further information: https://www.embopress.org/competing-interests

12) All Materials and Methods need to be described in the main text using our 'Structured Methods' format, which is required for all research articles. According to this format, the Methods section includes a Reagents and Tools Table (listing key reagents, experimental models, software and relevant equipment and including their sources and relevant identifiers) followed by a Methods and Protocols section describing the methods using a step-by-step protocol format. The aim is to facilitate adoption of the methodologies across labs. More information on how to adhere to this format as well as a downloadable template (.docx) for the Reagents and Tools Table can be found in our author guidelines: https://www.embopress.org/page/journal/14693178/authorguide#structuredmethods.

An example of a Method paper with Structured Methods can be found here: https://www.embopress.org/doi/full/10.1038/s44320-024-00037-6#sec-4

We would also welcome the submission of cover suggestions, or motifs to be used by our Graphics Illustrator in designing a cover.

As part of the EMBO publication's Transparent Editorial Process, EMBO reports publishes online a Review Process File (RPF) to accompany accepted manuscripts. This File will be published in conjunction with your paper and will include the referee reports, your point-by-point response and all pertinent correspondence relating to the manuscript.

You are able to opt out of this by letting the editorial office know (emboreports@embo.org). If you do opt out, the Review Process File link will point to the following statement: "No Review Process File is available with this article, as the authors have chosen not to make the review process public in this case."

I look forward to seeing a revised form of your manuscript when it is ready.

Yours sincerely,

Yehu Moran
Academic Editor
EMBO Reports

Referee #1:

This study monitors the allele frequency in a hybrid population generated from a cross between two yeast strains (Y55 and DBVPG1373) over several rounds of meiosis. It shows that a unique chromosomal translocation between chromosomes VIII and XVI leads to inviable spores and contributes to some of the changed allele frequencies in the evolved population. Chromosomal translocations are known to generate inviable or unhealthy gametes from a hybrid cross, resulting in changed allele frequency in the progeny population. This is especially well-documented in yeast (e.g., PMID 24814147, PMID 16489235, PMID 27571751, PMID 16951060, PMID 25039979, among others), for precisely the same reasons discussed by the authors (lines 303-307). In a series of studies done by Delneri et al. (PMID 12621434 and PMID 15105830), they even engineered translocated chromosomes to beautifully demonstrate their effects on spore viability and mitotic fitness. However, the authors did not mention these studies in shaping the manuscript and treated this phenomenon as a neglected question or underappreciated force (lines 28-29, 58, 72-74, 269-271, 324, and 332-333). It is misleading and disrespectful to existing literature. Moreover, several serious issues exist with the experimental designs and data interpretation. I have listed my specific comments below.

Major comments:

1) Although the authors emphasize that the primary selection in their experiments is from meiosis, the experimental design did not completely remove unsporulated diploid cells after meiosis, and did not control the ratio of a and alpha cells during the mating. Therefore, any allele that can enhance lyticase resistance (and the survival of diploids), mating efficiency, and sporulation efficiency will be selected throughout the entire process, leading to a biased allele frequency.
2) I do not understand why the right side of the breakpoint in chromosome XVI (Figure 1d) does not drop at a rate similar to the left end of chromosome VIII (Figure 1c). In the XVI-A spores, the immediate right side of the breakpoint always comes from Y55 (as shown in Figure 2e). Additionally, the frequency of this part in Figure 1e fluctuates significantly between cycles.
3) In lines 82-84, it is a wrong statement here since the allele frequency change exactly resulted from individual gamete fitness (i.e., some types of spores are dead).
4) In lines 205-222 and 285-294, the authors misinterpret the data of previous studies, leading to a misleading statement that alternate segregation is the dominant mode and the current data is very different from previous data. In those studies, the translocations involve large chromosomal fragments, which influence both meiotic recombination patterns and spore viability. Since many essential genes are included in both reciprocal translocated fragments, most adjacent-1 segregation events will yield no viable gametes. However, in the current case (Y55 x DBVPG1373), chromosome VIII in DBVPG137 only loses a small fragment (~15kb) that does not contain any essential genes, so XVI-A spores are still viable. It explains why a high proportion of adjacent-1 segregate products are observed. The differences in the translocated chromosomal size and gene content are sufficient to explain both results.
5) In lines 216-219, the data does not suggest that "frequent crossover events near the chromosome VIII breakpoint". Any crossover between the centromere and the breakpoint can generate this pattern and the crossover frequency is proportional to the distance between these two loci.
5) In lines 231-234, the authors need to conduct competition assays to conclude that there is no fitness difference between PT and XVI-A. Measuring growth curves is not sensitive enough to detect any fitness differences smaller than 5%.
6) In lines 250-253, I hope that the authors understand that crossovers occur between homologous chromosomes during meiosis I and their frequency depends on the distance between the breakpoint and centromere.
7) In lines 282-284, why do the authors think meiotic selection operates independently of gamete fitness here (and in many other places in the manuscript)? Their experiments show that observed segregation biases result from the inviability of a specific type of spores.

Minor comments:

1) In Figures 1c and 1d, please specify at what time points the meiotic and mitotic products were collected.
2) In Figure 2a, it should be ANOVA with Post Hoc Tests.
3) In Supplementary Figure 1, the cell and tetrad images are too small to be seen.
4) In Supplementary Figure 4, the authors need to provide more explanations to understand the figure.

Referee #2:

In this study Zeng et al. created a hybrid diploid baker's yeast strain by crossing the haploids Y55 and DBVPG1373. They identified 65,830 heterozygous SNP markers in the hybrid and used them to characterize marker segregations in meiosis. They observed the following:

1. Tetrads from sporulated hybrid strains yielded mostly 2 or 3 viable spores, whereas tetrads obtained from single strain backgrounds displayed mostly 4 viable spores.

2. Nanopore sequencing identified in DVBPG1373 a reciprocal translocation between a 15 kb region on Chr. VIII and a 373 kb region on Chr. XVI.

3. The most common tetrad pattern obtained by analyzing the sporulated Y55/DBVPG1373 hybrid was one which displayed crossover-associated adjacent-1 segregation (as detected in XVI-A spores). This pattern appeared more common than alternate segregation, which was thought to be the more common pattern formed by strains heterozygous for a reciprocal translocation. The authors provide an explanation for this difference that relates to the methods used to identify segregation events (complete tetrad analysis vs. random spore).

4. An implication of the crossover-associated adjacent-1 segregation pattern is that it "ultimately results in XVI-A spores persisting while their complementary VIII-A spores die in the population, thereby affecting allele frequencies." To test this idea the authors performed successive meiotic transfer experiments. This work demonstrated preferential inheritance of the 300 kb region in DBVPG1373 (part of the reciprocal translocation) in the meiotic transfer experiments. The authors performed fitness tests to argue that the preferential inheritance was not due to short-term effects on fitness, though long-term effects were not tested.

5. An analysis of 142 yeast strains identified on average 4 translocations per strain, indicating that translocations are common in yeast. The authors examined reciprocal translocation hybrids in strains with significant differences in similarity but containing the same reciprocal translocation as was seen in DBVPG1373. They showed that "Among the adjacent-1 segregation events, crossover-associated patterns were predominant, accounting for 76% to 92% of cases, highlighting RT-associated segregation distortion operates through chromosomal-level mechanisms that simultaneously affect multiple linked loci across extended genomic regions." They then argue that "Meiotic selection operates independently of gamete fitness, functioning through segregation biases that favor certain allelic combinations over others."

Major comments

This work shows that meiotic segregation patterns of a hybrid strain containing heterozygous reciprocal translocations can result in viable spore progeny that show in the translocated regions a departure from 50:50 allele frequencies. In the case examined it appears that specific combinations of Chr VIII and XVI regions from DBCPG1373 and Y55 were required for viability. Such an interaction, coupled with adjacent segregation, explains the observed segregation biases. In general, the work appears well executed and controlled and the results are interesting.

1. The authors argue that their findings "establish segregation bias associated with inter chromosomal translocation as a potent and underappreciated evolutionary force" but provide little evidence that such events are seen in nature. I feel that this conclusion is overstated. Two issues that were not discussed in the paper are: i. Do the authors have evidence indicating that outcrossing that would yield strains that display heterozygous reciprocal translocations? Values for outcrossing of yeasts vary widely with frequencies calculated to be as low as 1 in 100 to 1 in 50,000 vegetative divisions. Is there evidence for heterozygous reciprocal translocations in the ~3000 sequenced yeast genomes? ii. Is there any evidence of widespread duplications and deletions in yeast strains (perhaps clinical isolates) that would be consistent with crossover-associated adjacent-1 segregation from strains heterozygous for a reciprocal translocation? I wonder if such large duplications and deletions (in this case a 300 kb duplication and a 15 kb deletion) would be maintained due to fitness defects. In the experiments presented fitness effects were measured over a very small number of generations.

2. In my opinion the paper would be stronger if the authors first described the detection by nanopore sequencing of a reciprocal translocation in the DBVPG1373 strain and how crossing this strain to Y55 resulted in the observed spore viability patterns. These analyses, shown in Figures 2 and 3, set the stage for the meiotic transfer experiments presented in Figure 1.

3. The methods for how the meiotic transfer experiments were performed are unclear. On line 96 the authors indicate that meiotic products experienced a period of mitotic growth (16 hrs in YPD) "and were induced to cross again by changing the culture conditions subsequently." A puzzle to me is why the authors don't believe that spores in a single tetrad did not germinate and mate directly to each other in the mitotic growth phase. Such a protocol would indicate to me that the mating was not random and was not "induced." Such non-random mating could skew the patterns observed. On another note, the authors should indicate the genotype of the HO locus in the strains analyzed. My assumption is that they were both heterothallic (ho).

4. Line 468. "A total of 88 PT spores and 20 XVI-A spores were grown..." Do the authors mean spore clones?

5. Line 201. "We validated our method through WGS of 16 spores from 6 tetrads, which demonstrated perfect concordance between PCR-based determinations and sequencing-derived chromosomal states (Supplementary Fig. 11)." My assumption is that no four-spore viable tetrads were obtained in this set. I'm curious if the authors attempted to calculate the number of breakpoints that could provide an estimate of the number of crossovers detected in these crosses.

Point-by-Point Response

Editor:

1) A data availability section providing access to data deposited in public databases is missing. If you have not deposited any data, please add a sentence to the data availability section that explains that.

Response: Thank you for your reminder. We have now made the data publicly accessible, and the relevant information has been added to the Data Availability section.

2) Your manuscript contains statistics and error bars based on n=2. Please use scatter blots in these cases. No statistics should be calculated if n=2.

Response: Thank you for your comment. We have carefully reviewed all figures and statistical analyses in our manuscript and did not identify any instances where statistics or error bars were calculated based on n=2 biological replicates. Regarding the initial version of Figure 3g, which involves a comparison between two groups, we would like to clarify that this analysis compares two groups of data, each containing multiple samples, rather than two individual data points (n=2).

Referee #1:

This study monitors the allele frequency in a hybrid population generated from a cross between two yeast strains (Y55 and DBVPG1373) over several rounds of meiosis. It shows that a unique chromosomal translocation between chromosomes VIII and XVI leads to inviable spores and contributes to some of the changed allele frequencies in the evolved population. Chromosomal translocations are known to generate inviable or unhealthy gametes from a hybrid cross, resulting in changed allele frequency in the progeny population. This is especially well-documented in yeast (e.g., PMID 24814147, PMID 16489235, PMID 27571751, PMID 16951060, PMID 25039979, among others), for precisely the same reasons discussed by the authors (lines 303-307). In a series of studies done by Delneri et al. (PMID 12621434 and PMID 15105830), they even engineered translocated chromosomes to beautifully demonstrate their effects on spore viability and mitotic fitness. However, the authors did not mention these studies in shaping the manuscript and treated this phenomenon as a neglected question or underappreciated force (lines 28-29, 58, 72-74, 269-271, 324, and 332-333). It is misleading and disrespectful to existing literature. Moreover, several serious issues exist with the experimental designs and data interpretation. I have listed my specific comments below.

Response: We sincerely thank the reviewer for this important critique. We want to clarify that

our intention was never to suggest that the role of chromosomal translocations in generating inviable gametes is a novel or neglected phenomenon. We deeply respect the foundational contributions establishing the role of chromosomal rearrangements in speciation and reproductive isolation. While previous studies have extensively characterized how translocations act as reproductive barriers by producing inviable offspring, our study specifically investigates whether translocation-associated selection operates within the meiotic process itself to bias segregation pattern, ultimately influencing allele frequencies in populations. We have reorganized the Introduction and revised the related texts to address the misleading. We are grateful to the reviewer for the opportunity to improve the clarity of our manuscript. The relevant texts are as follows:

In Abstract: "*... Given the prevalence of inter-chromosomal translocations, biased segregation pattern may complement the established role of translocations in shaping evolutionary outcomes.*"

In Introduction: "*The effect of chromosomal rearrangements in producing unbalanced gametes with reduced viability has been extensively studied in the context of speciation and species isolation barriers (Charron et al, 2014; Delneri et al, 2003; Hou et al, 2014; Koszul et al, 2006; Leducq et al, 2016; Liti et al, 2006). From this perspective, chromosomal rearrangements act as physical barriers, especially in interspecies contexts, and represent important evolutionary forces. However, we sought to determine whether selection operate within meiotic processes in this context to influence gamete production. Traditionally, gametic selection in reproductive biology refers to differential gamete viability, motility advantages in sperm competition, and preferential fertilization success (Immler & Otto, 2018). Beyond these post-meiotic mechanisms, we reasoned that selection may act directly during the meiotic process, creating biased segregation pattern that significantly deviate from Mendelian expectations. Delneri et al have reported offspring viability rates exceeding theoretical expectations in hybrids carrying heterozygous reciprocal translocations (RTs), raising the possibility that non-random segregation patterns may occur during meiosis (Delneri et al., 2003)…*

…Detailed genomic and tetrad analyses uncovered that at translocation-linked loci, frequency changes stemmed from biased segregation pattern in translocation-containing quadrivalents, where adjacent-1 segregation associated with crossovers was strongly favored over alternate segregation, yielding gametes with improved viability compared to classical adjacent-1 products but altered genotypic compositions. This segregation bias shifts the distribution of gametic genotypes, thereby driving changes in population allele frequencies. Our study reveals that meiotic selection through biased segregation pattern can contribute significantly to allele frequency dynamics in populations."

In Results: "*…Given the prevalence of translocation, the segregation bias observed in*

our study may have implications for understanding allele frequency dynamics in natural populations where such structural variants are segregating."

In Discussion: "*In conclusion, our findings suggest that segregation bias associated with inter-chromosomal translocation may represent an important factor influencing allele frequency dynamics… Despite these limitations, our findings highlight evolutionary dynamics at the meiotic level that may help connect chromosomal biology and population genetics, providing insights into the forces that could contribute to genetic diversity in natural populations.*"

Major comments:

1) Although the authors emphasize that the primary selection in their experiments is from meiosis, the experimental design did not completely remove unsporulated diploid cells after meiosis, and did not control the ratio of a and alpha cells during the mating. Therefore, any allele that can enhance lyticase resistance (and the survival of diploids), mating efficiency, and sporulation efficiency will be selected throughout the entire process, leading to a biased allele frequency.

Response: We thank the reviewer for raising this important concern regarding potential confounding factors in our experimental design. We acknowledge that our protocol did not completely remove unsporulated diploid cells or control the MATa:MATα ratio during mating. However, we believe these factors are unlikely to account for the observed allele frequency changes for the following reasons. Regarding unsporulated diploid removal, common methods such as heat treatment or flow cytometry each introduce complications. Heat treatment can remove most diploid cells but causes substantial cell loss and introduces strong heat-stress selection. Flow cytometry requires nuclear staining with dyes such as Sytox Green, which may interfere with subsequent meiotic processes. Moreover, the sporulation efficiency in our experiments was relatively high (52.6%–58.6%), and the mitotic growth phase in each cycle was kept short, minimizing the potential contribution of unsporulated diploids to allele frequency changes.

Regarding MATa:MATα proportions, we analyzed the mating types of 212 randomly isolated spores and found no significant deviation from the expected 1:1 ratio in either the Y55 × DBVPG1373 cross (62 MATa vs. 49 MATα, $P = 0.22$) or the reciprocal cross (49 MATa vs. 52 MATα, $P = 0.76$), indicating no systematic bias between mating types. We also performed control crosses using YJM978 × DBVPG1373 and Y55 × BY4742, in which YJM978 shares the same chromosomal configuration as DBVPG1373, and BY4742 shares the same configuration as Y55, such that neither cross carries heterozygous reciprocal translocations. As shown in the figure below, we observed no significant allele frequency

changes near the breakpoints in these control crosses, whereas such changes were detected only in crosses carrying the translocations. This demonstrates that the observed allele frequency shifts are specifically associated with the translocation rather than selection on mating or sporulation efficiency.

We have incorporated these results into the revised manuscript. The relevant text is as follows:

"*...Furthermore, we analyzed the mating types of the sequenced spores and found no significant deviation from the expected 1:1 ratio in either the Y55 × DBVPG1373 cross (62 MATa vs. 49 MATα, chi-square test: P = 0.22) or the reciprocal cross (49 MATa vs. 52 MATα, chi-square test: P = 0.76), confirming that viability and genomic composition of spores are independent of mating type (Dataset EV5).*

To confirm that the observed allele frequency changes on chromosomes VIII and XVI are specifically associated with the reciprocal translocation, we performed additional control crosses using YJM978 × DBVPG1373 and Y55 × BY4742. In these crosses, YJM978 shares the same chromosomal configuration as DBVPG1373 on chromosomes VIII and XVI, and BY4742 shares the same configuration as Y55, such that neither cross carries heterozygous reciprocal translocations. We did not observe significant allele frequency changes near the breakpoints in these control crosses, whereas such changes were detected only in crosses carrying the translocations (Fig. 3B). These results indicate that the observed allele frequency changes on chromosomes VIII and XVI are specifically associated with the presence of chromosomal rearrangements rather than other factors."

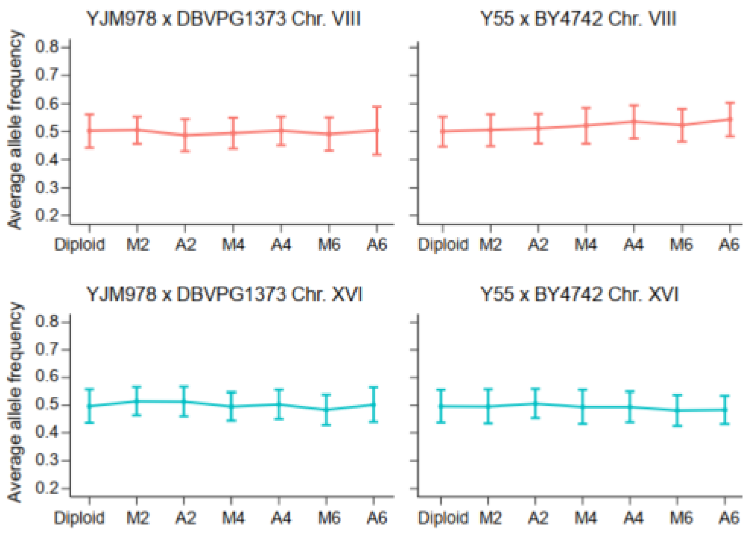

2) I do not understand why the right side of the breakpoint in chromosome XVI (Figure 1d) does not drop at a rate similar to the left end of chromosome VIII (Figure 1c). In the XVI-A spores, the immediate right side of the breakpoint always comes from Y55 (as shown in

Figure 2e). Additionally, the frequency of this part in Figure 1e fluctuates significantly between cycles.

Response: We thank the reviewer for this careful observation. The frequency changes in these two regions are actually consistent. The apparent discrepancy arose from the curve-fitting strategy used in the original figures. In the original Figures 1c–d, discrete SNP frequency data were fitted into continuous curves without accounting for SNP density differences along the chromosomes. Regions with higher SNP density contributed disproportionately to the fitted curves, making frequency changes appear more pronounced. On the right side of the chromosome XVI breakpoint, where SNP density is lower, local frequency changes were smoothed out by signals from more distant regions.

To address this, we revised our fitting approach by selecting one SNP every 200 bp to ensure equal contribution from different genomic regions. We also directly calculated allele frequency changes within 50 kb of the breakpoints on both chromosomes. As shown in the figure below, both regions start at 0.5 and change synchronously, stabilizing at approximately 0.6 for chromosome VIII and 0.4 for chromosome XVI after generation A3. This figure has been added to the revised manuscript as Appendix Figure S4.

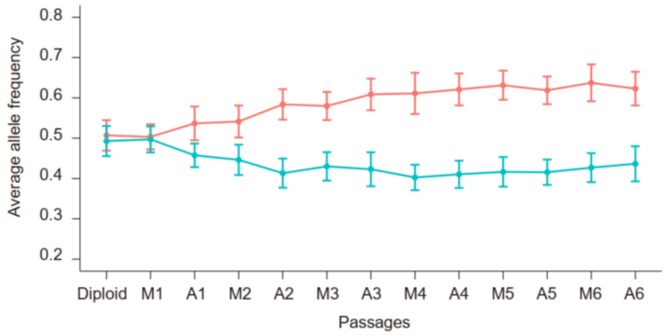

3) In lines 82-84, it is a wrong statement here since the allele frequency change exactly resulted from individual gamete fitness (i.e., some types of spores are dead).

Response: We thank the reviewer for pointing out this confusing statement. We have revised the description for clarity. The relevant text is as follows:

"*...Detailed genomic and tetrad analyses uncovered that at translocation-linked loci, frequency changes stemmed from biased segregation pattern in translocation-containing quadrivalents, where adjacent-1 segregation associated with crossovers was strongly favored over alternate segregation, yielding gametes with improved viability compared to classical adjacent-1 products but altered genotypic compositions. This segregation bias shifts the distribution of gametic genotypes, thereby driving changes in population allele frequencies...*"

4) In lines 205-222 and 285-294, the authors misinterpret the data of previous studies, leading

to a misleading statement that alternate segregation is the dominant mode and the current data is very different from previous data. In those studies, the translocations involve large chromosomal fragments, which influence both meiotic recombination patterns and spore viability. Since many essential genes are included in both reciprocal translocated fragments, most adjacent-1 segregation events will yield no viable gametes. However, in the current case (Y55 x DBVPG1373), chromosome VIII in DBVPG137 only loses a small fragment (~15kb) that does not contain any essential genes, so XVI-A spores are still viable. It explains why a high proportion of adjacent-1 segregate products are observed. The differences in the translocated chromosomal size and gene content are sufficient to explain both results.

Response: We thank the reviewer for this insightful comment. The reviewer correctly points out that in many previous studies involving large reciprocal translocations, adjacent-1 segregation products are often inviable because both translocated segments contain essential genes, leading to an apparent predominance of alternate segregation among viable progeny. In our system, XVI-A spores remain viable because the small translocated fragment on chromosome VIII lacks essential genes, allowing detection of adjacent-1 segregation events that might otherwise be masked. However, we noted that in Loidl et al. (1998), the translocation t(I,VI) had breakpoints at 10–14 kb on *I*L and 133–145 kb on *VI*L, resulting in a translocated segment length comparable to ours. There are also no essential genes within the 1-15 kb region on *I*L. Despite this similarity, predominant alternate segregation was observed in that study, suggesting that segment length alone may not fully explain the segregation pattern in Y55 × DBVPG1373 hybrids. We have revised the Discussion to clarify this comparison. The relevant text is as follows:

"In quadrivalents formed by heterozygous RT carriers, alternate segregation is typically the most frequently observed mode, followed by adjacent-1 segregation (Benet et al, 2005; Loidl et al., 1998; Oliver-Bonet et al, 2001; Verdoni et al, 2021). In some of these studies, adjacent-1 segregation products may be underrepresented among viable progeny when both translocated segments contain essential genes, potentially contributing to the observed predominance of alternate segregation. In our system, XVI-A spores remain viable because the small translocated fragment on chromosome VIII does not contain essential genes, allowing us to detect adjacent-1 segregation events that might otherwise be masked. However, our methodological approach, combining tetrad dissection with PCR-based karyotype analysis, revealed a strikingly different segregation pattern preference. In Y55 (MATa) and DBVPG1373 (MATα) hybrids, we observed an alternate : adjacent-1 segregation ratio of 1:2.7. This unusual pattern does not appear to result solely from the relatively short length of the translocated segment on chromosome VIII. In a previous study of heterozygous translocations involving t(I,VI) in yeast, breakpoints were mapped between positions 10-14 kb

on IL and 133-145 kb on VIL (Loidl et al., 1998). Importantly, there are no essential genes within the 1-15 kb region on IL. Although the translocated segment length in t(I,VI) is comparable to that in our study, predominant alternate segregation was observed."

5) In lines 216-219, the data does not suggest that "frequent crossover events near the chromosome VIII breakpoint". Any crossover between the centromere and the breakpoint can generate this pattern and the crossover frequency is proportional to the distance between these two loci.

Response: We thank the reviewer for this insightful comment. We agree that our original phrasing was inaccurate. As the reviewer correctly noted, any crossover within the interstitial interval (between the centromere and the breakpoint) can generate the observed segregation pattern. Upon further analysis, we recognized that crossovers in either chromosome VIII or XVI can produce the pattern of two PT spores and one XVI-A spore—a possibility we had overlooked. We examined crossover events in both chromosomes using sequenced spores. Among 88 PT spores, we detected 9 crossovers on chromosome XVI versus 5 on chromosome VIII. In the reciprocal cross (84 PT spores), we detected 14 crossovers on chromosome XVI versus 7 on chromosome VIII. Mapping confirmed that these events were distributed throughout the intervals with no positional bias. We have revised the manuscript accordingly. The relevant text is as follows:

"Most remarkably, we discovered a prevalent novel pattern (28.1%, 25 tetrads) characterized by two PT spores and one XVI-A spore (Fig. 4A). The most parsimonious explanation for this pattern is that it results from adjacent-1 segregation coupled with a crossover event occurring in the interstitial region between the centromere and the breakpoint of either chromosome VIII or XVI. Such a crossover would alter the linkage between the centromere and the translocation breakpoint, resulting in the production of two PT spores, one XVI-A spore, and one inviable VIII-A spore (Fig. 4D).

To test this hypothesis, we analyzed the genomic composition of randomly selected spores to identify recombination events. Specifically, we searched for genotype transitions indicative of crossovers (i.e., a switch in parental genotype extending from the recombination site to the breakpoint) in chromosomes VIII and XVI in PT spores, which could arise either from alternate segregation or as viable products of crossover-associated adjacent-1 segregation (Methods, Fig. 4E). Among 88 PT spores, 5 and 9 spores exhibited valid crossovers on chromosomes VIII and XVI, respectively. Similarly, in 84 PT spores derived from the reciprocal cross, we identified 7 and 14 crossovers on chromosomes VIII and XVI, respectively. These findings provide molecular evidence supporting the occurrence of crossovers in the predicted intervals, with a higher frequency observed on chromosome XVI.

The distribution of crossover sites appeared random, with no significant positional bias (Fig. 4E). Compared to canonical adjacent-1 segregation which producing two XVI-A spores, this segregation pattern yields a greater number of viable spores."

5) In lines 231-234, the authors need to conduct competition assays to conclude that there is no fitness difference between PT and XVI-A. Measuring growth curves is not sensitive enough to detect any fitness differences smaller than 5%.

Response: We thank the reviewer for this suggestion. As recommended, we performed 8 independent competitive growth experiments between PT and XVI-A spores. The proportions of the two spore types remained stable over four passages of cultivation (Appendix Fig. S10, shown below). Both the result and the methodology for the competitive growth experiments have been added to the revised manuscript. The relevant texts are as follows:

In Results: *"…We also performed eight independent pairwise competitive growth experiments between PT and XVI-A spores, and the results showed that the proportions of the two spore types remained relatively stable over four passages of cultivation (Appendix Fig. S10)…"*

In Methods: *"Pairwise competition experiments were conducted between the PT and XVI-A strains collected from random spore analysis. The strains were independently grown in YPD medium to stationary phase. Random pairwise strains with the same mating type were then mixed at a 1:1 ratio and inoculated into fresh YPD medium at a 1:300 dilution. Cells were incubated at 30 °C with shaking for 12 h, corresponding to the exponential growth phase (8 generations). Cultures were subsequently passaged into fresh medium using the same dilution ratio. At each passage, samples were collected for genomic DNA extraction and library preparation, following the procedures described in the Genome extraction and sequencing section. To quantify the relative abundance of PT and XVI-A cells at each passage, parental SNP loci were used as genetic markers. Whole-genome sequencing data from each parental strain were subjected to variant calling using bcftools, with parameters identical to those described in the Detection of SNP markers between parental strains section. The relative proportions of PT and XVI-A cells of each pair were determined by the mean allele frequencies of the SNP locus from the initial strains."*

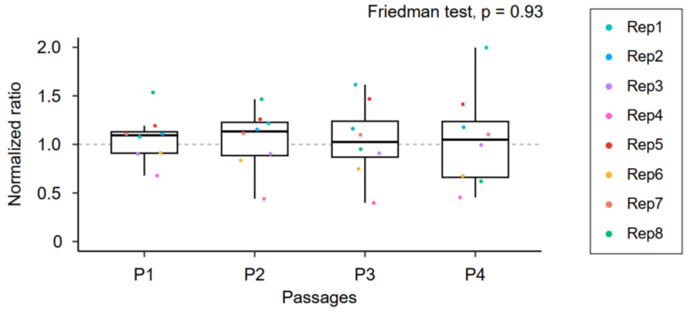

6) In lines 250-253, I hope that the authors understand that crossovers occur between homologous chromosomes during meiosis I and their frequency depends on the distance between the breakpoint and centromere.

Response: We agree that our original wording was imprecise. We have removed the phrase "highlighting the widespread occurrence of crossover events during adjacent-1 segregation" from the revised manuscript.

7) In lines 282-284, why do the authors think meiotic selection operates independently of gamete fitness here (and in many other places in the manuscript)? Their experiments show that observed segregation biases result from the inviability of a specific type of spores.

Response: We have revised this and similar statements throughout the manuscript.

Minor comments:

1) In Figures 1c and 1d, please specify at what time points the meiotic and mitotic products were collected.

Response: We have specified the time points for collection of meiotic and mitotic products in the figure legend of Figures 1. The relevant text is as follows:

"*(A) Flowchart of successive cross-meiosis experiments in a yeast hybrid from the cross between Y55 and DBVPG1313. Mn represents the nth round of meiotic products, and An represents the nth round of mitotic growth products. Genomic DNA was extracted at both Mn and An time points for allele frequency determination by sequencing.*

(C-D), Allele frequency across chromosomes VIII and XVI changes from the initial hybrid diploid to the sixth round. The dotted line represents the initial hybrid, the solid lines represent the meiotic products collected after 7 days of sporulation (M1-M6), and the dashed lines are mitotic growth products collected after 16 hours of growth in YPD medium (A1-A6). Curves were fitted from discrete SNP allele frequencies and one SNP marker was selected every 200 bp for curve fitting for minimizing the effect of variable SNP density across regions. Values from the same round are shown in the same color. The cycle of sporulation (7 days) followed by mitotic growth (16 hours) and mating (72 hours) was repeated for six rounds."

2) In Figure 2a, it should be ANOVA with Post Hoc Tests.

Response: We have revised Figure 2a to use ANOVA with post hoc tests as suggested.

3) In Supplementary Figure 1, the cell and tetrad images are too small to be seen.

Response: We have enlarged the cell and tetrad images in this figure (now Appendix Fig. S1)

to improve visibility.

4) In Supplementary Figure 4, the authors need to provide more explanations to understand the figure.

Response: We have expanded the figure legend (now Fig. EV2) with more detailed explanations to facilitate understanding of the data. The same applies to Supplementary Figure 5 (now Fig. EV3).

Referee #2:

In this study Zeng et al. created a hybrid diploid baker's yeast strain by crossing the haploids Y55 and DBVPG1373. They identified 65,830 heterozygous SNP markers in the hybrid and used them to characterize marker segregations in meiosis. They observed the following:

1. Tetrads from sporulated hybrid strains yielded mostly 2 or 3 viable spores, whereas tetrads obtained from single strain backgrounds displayed mostly 4 viable spores.
2. Nanopore sequencing identified in DVBPG1373 a reciprocal translocation between a 15 kb region on Chr. VIII and a 373 kb region on Chr. XVI.
3. The most common tetrad pattern obtained by analyzing the sporulated Y55/DBVPG1373 hybrid was one which displayed crossover-associated adjacent-1 segregation (as detected in XVI-A spores). This pattern appeared more common than alternate segregation, which was thought to be the more common pattern formed by strains heterozygous for a reciprocal translocation. The authors provide an explanation for this difference that relates to the methods used to identify segregation events (complete tetrad analysis vs. random spore).
4. An implication of the crossover-associated adjacent-1 segregation pattern is that it "ultimately results in XVI-A spores persisting while their complementary VIII-A spores die in the population, thereby affecting allele frequencies." To test this idea the authors performed successive meiotic transfer experiments. This work demonstrated preferential inheritance of the 300 kb region in DBVPG1373 (part of the reciprocal translocation) in the meiotic transfer experiments. The authors performed fitness tests to argue that the preferential inheritance was not due to short-term effects on fitness, though long-term effects were not tested.
5. An analysis of 142 yeast strains identified on average 4 translocations per strain, indicating that translocations are common in yeast. The authors examined reciprocal translocation hybrids in strains with significant differences in similarity but containing the same reciprocal translocation as was seen in DBVPG1373. They showed that "Among the adjacent-1 segregation events, crossover-associated patterns were predominant, accounting for 76% to 92% of cases, highlighting RT-associated segregation distortion operates through

chromosomal-level mechanisms that simultaneously affect multiple linked loci across extended genomic regions." They then argue that "Meiotic selection operates independently of gamete fitness, functioning through segregation biases that favor certain allelic combinations over others."

Major comments

This work shows that meiotic segregation patterns of a hybrid strain containing heterozygous reciprocal translocations can result in viable spore progeny that show in the translocated regions a departure from 50:50 allele frequencies. In the case examined it appears that specific combinations of Chr VIII and XVI regions from DBCPG1373 and Y55 were required for viability. Such an interaction, coupled with adjacent segregation, explains the observed segregation biases. In general, the work appears well executed and controlled and the results are interesting.

Response: It is our pleasure to thank the reviewer for the evaluation and recognition of our work.

1. The authors argue that their findings "establish segregation bias associated with inter chromosomal translocation as a potent and underappreciated evolutionary force" but provide little evidence that such events are seen in nature. I feel that this conclusion is overstated. Two issues that were not discussed in the paper are: i. Do the authors have evidence indicating that outcrossing that would yield strains that display heterozygous reciprocal translocations? Values for outcrossing of yeasts vary widely with frequencies calculated to be as low as 1 in 100 to 1 in 50,000 vegetative divisions. Is there evidence for heterozygous reciprocal translocations in the ~3000 sequenced yeast genomes? ii. Is there any evidence of widespread duplications and deletions in yeast strains (perhaps clinical isolates) that would be consistent with crossover-associated adjacent-1 segregation from strains heterozygous for a reciprocal translocation? I wonder if such large duplications and deletions (in this case a 300 kb duplication and a 15 kb deletion) would be maintained due to fitness defects. In the experiments presented fitness effects were measured over a very small number of generations.

Response: We thank the reviewer for these thoughtful comments. Regarding outcrossing frequency, while early estimates suggested rates as low as once per 50,000 generations (Ruderfer et al., 2006), more recent studies indicate that outcrossing may occur at considerably higher frequencies under natural conditions. Murphy and Zeyl (2010) demonstrated that outcrossing rates can reach 10–25% when dense populations of sporulated yeast germinate simultaneously. Furthermore, population genomic analyses of 1,011 *S. cerevisiae* isolates revealed mosaic genome structures in approximately 15% of strains, providing direct evidence of historical outcrossing events (Peter et al., 2018). Given that

reciprocal translocations are widespread among *S. cerevisiae* strains, heterozygous configurations could arise in nature more frequently than previously appreciated.

Regarding whether large-scale chromosomal imbalances could be maintained in natural populations, Peter et al. (2018) found that 19.1% of natural *S. cerevisiae* strains contain chromosomal aneuploidies and large segmental duplications, and O'Donnell et al. (2023) identified approximately 44% of strains harboring deletions exceeding 15 kb among 140 telomere-to-telomere assemblies. These observations indicate that chromosomal imbalances of similar magnitude to those arising from adjacent-1 segregation can be tolerated in natural populations. In response to these valuable suggestions, we have revised our concluding statements and incorporated these points into the Discussion section while acknowledging the limitations of our study. The relevant text is as follows:

"Chromosomal rearrangements such as RTs are widespread across diverse organisms (Damas et al., 2021; Mérot et al., 2020). While outcrossing rates in S. cerevisiae have been estimated to be as low as once per 50,000 generations under certain assumptions (Ruderfer et al, 2006), recent studies suggest that outcrossing may occur at considerably higher frequencies under natural conditions. When entire dense populations of sporulated yeast germinate simultaneously, outcrossing can occur at rates of 10-25% (Murphy & Zeyl, 2010). Furthermore, population genomic analyses of 1,011 S. cerevisiae isolates have revealed mosaic genome structures in 150 strains, indicative of historical outcrossing and hybridization events (Peter et al, 2018). These findings suggest that opportunities for generating heterozygous reciprocal translocations through outcrossing are more frequent than previously appreciated.

...

Although XVI-A spores in our study carried a ~300 kb duplication and a ~15 kb deletion, such chromosomal imbalances may nonetheless persist in populations over extended periods. Population genomic analyses of 1,011 S. cerevisiae isolates revealed that 19.1% of natural strains contain chromosomal aneuploidies and large segmental duplications (Peter et al., 2018). Furthermore, genome structural analysis of 140 telomere-to-telomere assemblies identified 62 strains harboring deletions exceeding 15 kb (O'Donnell et al., 2023). These observations indicate that large-scale chromosomal imbalances, including those potentially arising from adjacent-1 segregation, can be tolerated and maintained in natural populations."

2. In my opinion the paper would be stronger if the authors first described the detection by nanopore sequencing of a reciprocal translocation in the DBVPG1373 strain and how crossing this strain to Y55 resulted in the observed spore viability patterns. These analyses,

shown in Figures 2 and 3, set the stage for the meiotic transfer experiments presented in Figure 1.

Response: We thank the reviewer for this suggestion. After careful consideration, we have retained the current structure as it better reflects our study's central focus: demonstrating rapid genomic evolution driven by meiotic-level selection. Our “phenomenon-to-mechanism” organization allows readers to first appreciate the striking observation of dramatic allele frequency shifts within a remarkably short timeframe (Figure 1), before understanding the underlying chromosomal mechanism (Figures 2-4). Beginning with the reciprocal translocation characterization would shift emphasis toward chromosomal structure, potentially obscuring our central message that meiotic-level selection acts as a rapid evolutionary force. We believe the current structure more effectively conveys the evolutionary significance of our findings.

3. The methods for how the meiotic transfer experiments were performed are unclear. On line 96 the authors indicate that meiotic products experienced a period of mitotic growth (16 hrs in YPD) "and were induced to cross again by changing the culture conditions subsequently." A puzzle to me is why the authors don't believe that spores in a single tetrad did not germinate and mate directly to each other in the mitotic growth phase. Such a protocol would indicate to me that the mating was not random and was not "induced." Such non-random mating could skew the patterns observed. On another note, the authors should indicate the genotype of the HO locus in the strains analyzed. My assumption is that they were both heterothallic (ho).

Response: In *Saccharomyces cerevisiae*, diploidy can be restored after meiosis through intratetrad mating (between spores from the same tetrad) or outcrossing (between spores from different tetrads). Previous studies estimated that approximately 10–25% of matings occur via outcrossing, with the majority arising from intratetrad mating (Murphy and Zeyl, 2010, PMID: 20463964; Goddard et al., 2010, PMID: 19691498). However, these estimates were based on laboratory strains. Wild-type yeast isolates exhibit delayed interest in sex after germination, characterized by asynchronous germination timing among spores, whereby spores undergo several rounds of budding before mating, rupturing the ascus wall and reducing intratetrad mating (McClure et al., 2018, PMID: 30355051). This effect is likely more pronounced in our system, as the heterozygosity of our parental strains further enhances asynchronous germination among spores within the same tetrad. Additionally, all mating steps were conducted in liquid culture, promoting thorough mixing and random mating among spores from different tetrads.

To directly test mating randomness, we performed a fluorescent marker assay. MATα spores from two different tetrads were labeled with GFP or mScarlet, respectively, and mixed

at equal ratios to mate with MATa spores. As the figure below showed, flow cytometry revealed no significant difference in the proportions of GFP- and mScarlet-positive diploids, confirming random mating. This result has been added to the Results section. Regarding the HO locus, both parental strains (Y55 and DBVPG1373) are heterothallic, which we have now clarified in the Methods section. The relevant text in Result is as follows:

"…To confirm that mating was random and not biased toward sibling spores, we performed a fluorescent marker assay. MATα spores from two different tetrads were labeled with GFP or mScarlet, respectively, and mixed at equal ratios to mate with MATa spores. Flow cytometry revealed no significant difference in the proportions of GFP- and mScarlet-positive diploids (Fig. EV1)…"

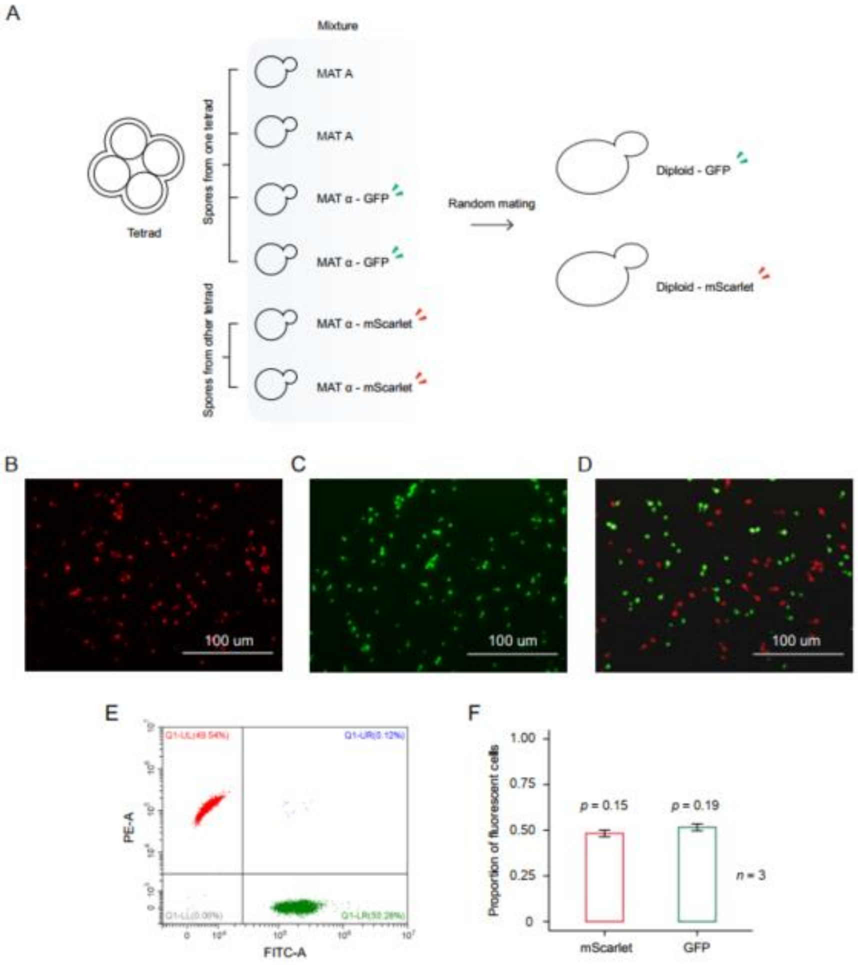

4. Line 468. "A total of 88 PT spores and 20 XVI-A spores were grown..." Do the authors mean spore clones?

Response: Yes, we meant spore clones. We have revised the text accordingly.

5. Line 201. "We validated our method through WGS of 16 spores from 6 tetrads, which demonstrated perfect concordance between PCR-based determinations and

sequencing-derived chromosomal states (Supplementary Fig. 11)." My assumption is that no four-spore viable tetrads were obtained in this set. I'm curious if the authors attempted to calculate the number of breakpoints that could provide an estimate of the number of crossovers detected in these crosses.

Response: Regarding the first point, the reviewer's assumption is correct—no four-spore viable tetrads were obtained in this validation set. Regarding crossover analysis, we did examine crossover events in the sequenced spores by scanning SNP markers within the interstitial intervals (regions between centromeres and translocation breakpoints) of chromosomes VIII and XVI. A crossover was identified when the genotype at the centromere differed from that at the breakpoint. Among 88 PT spores, we detected 5 and 9 crossovers on chromosomes VIII and XVI, respectively. In the reciprocal cross (85 PT spores), we detected 7 and 14 crossovers on chromosomes VIII and XVI, respectively, with no apparent positional bias. These findings provide molecular evidence for the "crossover-associated adjacent-1 segregation", wherein crossovers in the interstitial region rescue spore viability by altering segregation outcomes. We have added detailed descriptions of crossover detection methods and results to the revised manuscript. The relevant text in Result is as follows:

"To test this hypothesis, we analyzed the genomic composition of randomly selected spores to identify recombination events. Specifically, we searched for genotype transitions indicative of crossovers (i.e., a switch in parental genotype extending from the recombination site to the breakpoint) in chromosomes VIII and XVI in PT spores, which could arise either from alternate segregation or as viable products of crossover-associated adjacent-1 segregation (Methods, Fig. 4E). Among 88 PT spores, 5 and 9 spores exhibited valid crossovers on chromosomes VIII and XVI, respectively. Similarly, in 84 PT spores derived from the reciprocal cross, we identified 7 and 14 crossovers on chromosomes VIII and XVI, respectively. These findings provide molecular evidence supporting the occurrence of crossovers in the predicted intervals, with a higher frequency observed on chromosome XVI. The distribution of crossover sites appeared random, with no significant positional bias (Fig. 4E). Compared to canonical adjacent-1 segregation which producing two XVI-A spores, this segregation pattern yields a greater number of viable spores."

1st Revision - Editorial Decision 18th Mar 2026

Dear Dr. Liu

Thank you for the submission of your revised manuscript to our offices. We have now received the enclosed reports from the referees that were asked to assess it. As you will see below, EMBOR-2025-62721V2 still has comments that I would like you to address before we can proceed with the final decision on your manuscript.

In particular, Referee #2 remains unconvinced regarding the question of random mating. This is not a minor point and requires further attention. I would therefore ask you to carefully consider the experiment suggested by the referee. If this experiment is feasible and can be carried out within a reasonable timeframe, I encourage you to perform it.

If, however, you believe that performing this experiment is not straightforward, please provide a detailed explanation of the technical or conceptual limitations, and clarify why you consider the experiment unnecessary.

Please note that, according to our standard editorial policy, manuscripts at this stage are normally not considered for further experimental revision and would typically be rejected. I am therefore making an exception in this case and offering you the opportunity to address this point before a final decision is reached.

I look forward to seeing a new revised version of your manuscript as soon as possible.

Kind regards,
Yehu Moran
Academic Editor
EMBO Reports

Referee #1:

The authors have corrected all inaccurate statements and addressed most of my previous concerns. The revised manuscript is much improved.

Referee #2:

In general the authors worked hard to respond to my concerns. However, at least for me the question about random mating doesn't seem to have been addressed. The authors tested mating randomness by integrating GFP or mScarlet into MATalpha spore clones from two different tetrads, after which the spore clones were mixed at equal ratios to mate with MATa spores. It is not surprising that they obtained similar proportions of GFP and mScarlet positive diploids because this involves the mating of vegetative cells rather than asking if there is a bias in intra-tetrad mating due to MATa and MATalpha spores present within a tetrad.

Perhaps the simplest test would be if the authors created two Y55 x DBVPG1373 diploid strains, one of which was heterozygous for a GFP marker (at a specific locus in Y55 that does not impact fitness and is not linked to mating type or to chromosomes involved in the translocation) and another which was heterozygous for a mScarlet marker at the same locus. One could then perform the sporulation procedure described in the methods (7 day sporulation), then mix the two sporulated cultures prior to the 16 hr mitotic growth step described in the methods. The mixed culture after a 16 hr mitotic growth would then be plated to single colonies on YPD to determine the frequency of red only, green only and red + green colonies. If there was a strong bias towards intra-tetrad mating then one would expect a low proportion (<1/8 of the colonies that form diploids, I believe) of red + green colonies.

Referee #1:

The authors have corrected all inaccurate statements and addressed most of my previous concerns. The revised manuscript is much improved.

Response: We sincerely thank the reviewer for the positive assessment of our revised manuscript.

Referee #2:

In general the authors worked hard to respond to my concerns. However, at least for me the question about random mating doesn't seem to have been addressed. The authors tested mating randomness by integrating GFP or mScarlet into MATalpha spore clones from two different tetrads, after which the spore clones were mixed at equal ratios to mate with MATa spores. It is not surprising that they obtained similar proportions of GFP and mScarlet positive diploids because this involves the mating of vegetative cells rather than asking if there is a bias in intra-tetrad mating due to MATa and MATalpha spores present within a tetrad.

Perhaps the simplest test would be if the authors created two Y55 x DBVPG1373 diploid strains, one of which was heterozygous for a GFP marker (at a specific locus in Y55 that does not impact fitness and is not linked to mating type or to chromosomes involved in the translocation) and another which was heterozygous for a mScarlet marker at the same locus. One could then perform the sporulation procedure described in the methods (7 day sporulation), then mix the two sporulated cultures prior to the 16 hr mitotic growth step described in the methods. The mixed culture after a 16 hr mitotic growth would then be plated to single colonies on YPD to determine the frequency of red only, green only and red + green colonies. If there was a strong bias towards intra-tetrad mating then one would expect a low proportion (<1/8 of the colonies that form diploids, I believe) of red + green colonies.

Response: We thank the reviewer for this insightful suggestion. Following the reviewer's recommendation, we performed a new fluorescent marker experiment specifically designed to distinguish inter-tetrad from intra-tetrad mating during the cross-sporulation cycle (Response Fig. 1; also shown in Fig. EV1). Under idealized conditions, the expected frequency of G+M diploids would be 1/8. In the actual experiment, however, this observed frequency is necessarily reduced by incomplete sporulation, limited spore viability, and less-than-perfect mating efficiency. Therefore, a G+M frequency below 1/8 does not by itself indicate that intra-tetrad mating is dominant.

To account for these factors, we measured the relevant parameters experimentally and incorporated them into a quantitative analysis. Across six experiments, including three biological replicates for each reciprocal cross, the estimated inter-tetrad mating frequency

ranged from 51.2% to 91.5%, with a mean of 73.0%. These results show that mating in our experimental system is not dominated by intra-tetrad mating; rather, inter-tetrad mating is the major route of diploid re-formation under our conditions. We apologize that the wording in the previous version was overly strong and have revised the relevant text accordingly. The estimation procedure has been added to the Appendix Supplementary Methods.

Because intra-tetrad mating is nevertheless present, we further evaluated its impact on allele-frequency dynamics (Appendix Supplementary Methods). Our simulations indicate that intra-tetrad mating moderates the effect by slowing allele-frequency change relative to a fully inter-tetrad or fully random mating regime, but does not alter the central conclusion that translocation-associated selection during meiosis can drive substantial and sustained allele-frequency change in this system (Response Fig. 2; also shown in Appendix Fig. S13).

We have incorporated these results into the Results and Discussion sections, and the revised text is as follows:

In Results: *"…Then the meiotic products from each group experienced a period of mitotic growth and were induced to cross again by changing the culture conditions subsequently. To determine whether mating during this step occurred predominantly within tetrads or between tetrads, we performed a fluorescent marker assay. We estimated that approximately 73% of newly formed diploids arose from inter-tetrad mating (Fig. EV1; Appendix Supplementary Methods), indicating that inter-tetrad mating accounts for the majority of diploid re-formation events under our experimental conditions. The products of the secondary round of cross were then subjected to the next round of…"*

In Discussion: *"Our marker-based assay showed that diploid re-formation during the cross-sporulation cycle is dominated by inter-tetrad mating, with intra-tetrad mating accounting for the remaining fraction. We therefore explicitly evaluated the impact of intra-tetrad mating on allele-frequency dynamics. These analyses showed that intra-tetrad mating moderates the effect by slowing allele-frequency change relative to a fully inter-tetrad or fully random mating regime (Appendix Fig. S13, Appendix Supplementary Methods). Nevertheless, it does not alter the central conclusion that translocation-associated selection during meiosis can drive rapid allele-frequency change in this system."*

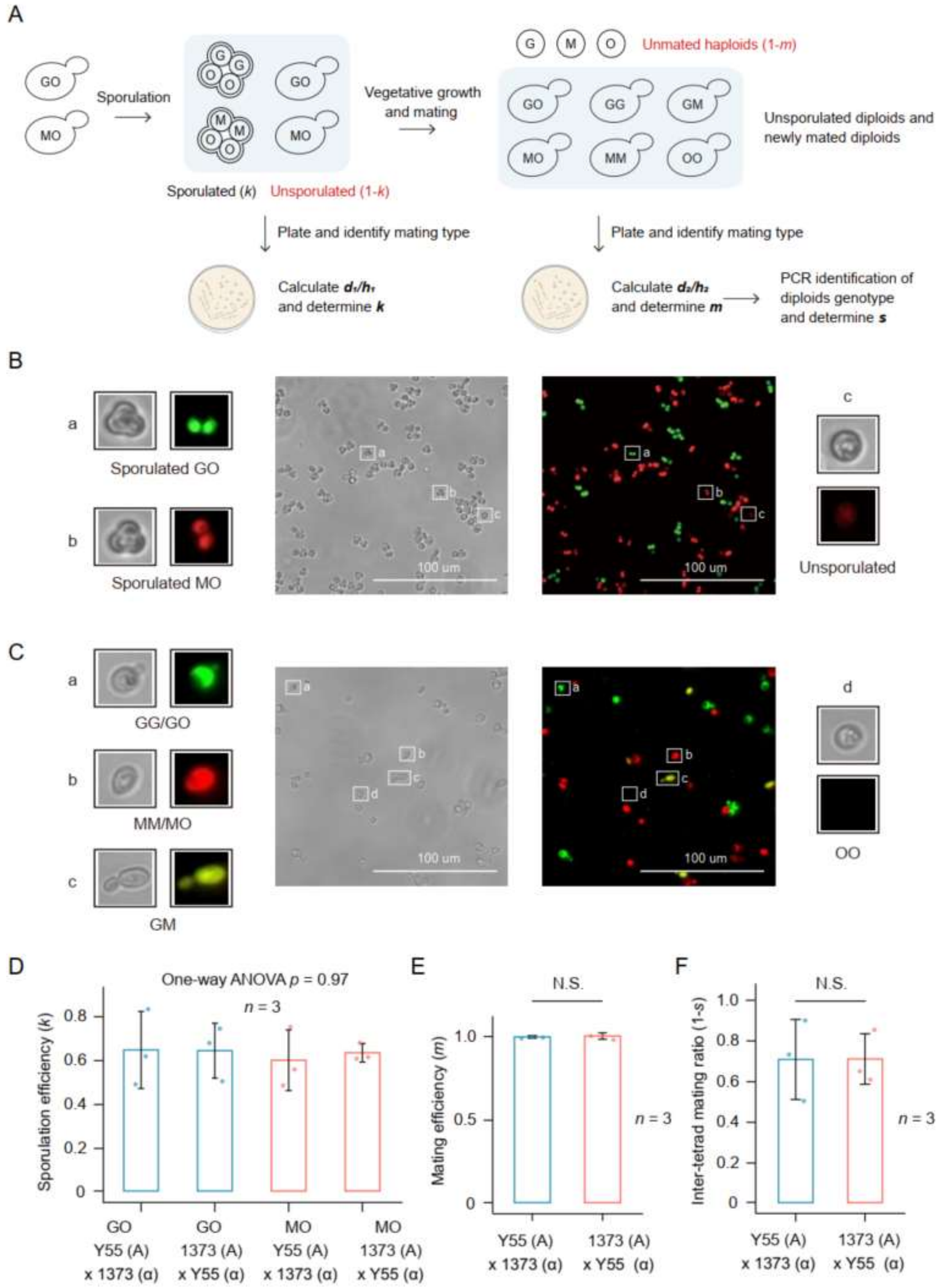

Response Fig. 1: Quantitative analysis of intra- and inter-tetrad mating of spores. (A) Schematic illustration of the experiment. GO (GFP/-) and MO (mScarlet/-) strains were sporulated separately and then mixed at equal proportions in rich medium to allow vegetative growth and mating. Cell populations after sporulation and after mating were plated to determine the numbers of haploids and diploids, as well as the proportion of GM (GFP/mScarlet) cells, which were used to estimate the relative proportions of intra- and inter-tetrad mating. *k*,

sporulation rate; *m*, mating efficiency; *s*, proportion of intra-tetrad mating; d1/h1 and d2/h2, observed diploid-to-haploid ratios at the corresponding stages. Genotype: GG, GFP/GFP; MM, mScarlet/mScarlet; OO, -/-.

(B) Representative microscopy images of cells after sporulation (before vegetative growth). Bright-field images are shown on the left, and the merged GFP and mScarlet fluorescence images are shown on the right. Representative cells marked by white boxes are magnified besides.

(C) Representative microscopy images of cells after mating. Bright-field images are shown on the left, and merged GFP and mScarlet fluorescence images are shown on the right. Representative cells marked by white boxes are magnified besides.

(D) Sporulation rates of the four different initial diploid genotypes. Each group includes three biological replicates. No significant difference among groups was detected by one-way ANOVA ($p = 0.97$).

(E) Mating efficiency of the reciprocal crosses. Each group includes three biological replicates. No significant difference was detected by two-sided Student's t-test ($p = 0.66$).

(F) Proportion of inter-tetrad mating of the reciprocal crosses. Each group includes three biological replicates. No significant difference was detected by two-sided Student's t-test ($p = 0.99$).

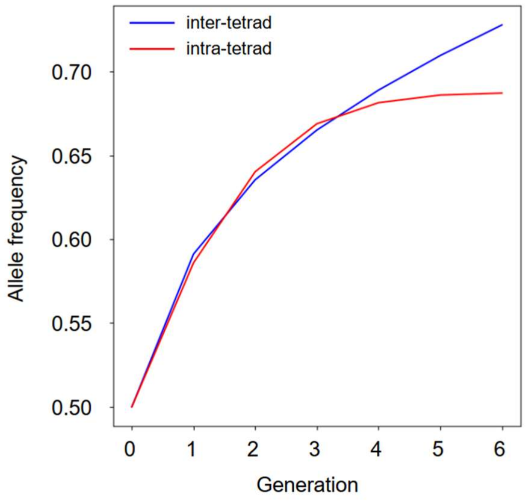

Response Fig. 2: *In silico* simulations of allele-frequency shifts across consecutive cycles of sporulation and mating under different mating modes.

2nd Revision - Editorial Decision 7th May 2026

Dear Dr. Liu

Thank you for the submission of your revised manuscript to our offices. Please note our editorial assistance team flagged several technical issues that must be addressed and fixed before we can accept your paper.

I look forward to seeing a new revised version of your manuscript as soon as possible.

Yehu Moran
Academic Editor
EMBO Reports

FIGURES
- Each main and EV figure needs to be provided as individual production quality Figure file.

MANUSCRIPT FILE

- Place Disclosure and competing interest statement after Acknowledgments
- Remove Author contributions from the manuscript text since you already provided the roles and contributions for each author via our submission system.
- "Supplementary" should not be used as nomenclature, so please just use "Appendix" instead of "Appendix Supplementary Methods"

APPENDIX
- Provide a title page that has the title (Appendix to [Title of the Manuscript]) and a short table of contents listing each item and its page number
- Provide page numbers throughout the file
- Of the Appendix Methods need to remain in the file, please label them as Appendix Methods and remove the "Supplementary"

DATASETS
- Upload each Dataset as a separate Data set file (Dataset EV1, Dataset EV2, etc.)
- The sheet name in Dataset EV1 and EV2 needs to be corrected to Dataset EV1 and Dataset EV2 (instead of Table S1 and Table S2)

DATA ISSUES
Figure Legends - Comments
- Please note that the exact p values are not provided in the legend of figure 2A
- Please note that the box plots need to be defined in terms of minima, maxima, centre, bounds of box and whiskers, and percentile in the legends of figures 4A
- Please note that information related to n is missing in the legends of figures 2A, 4A
- Although 'n' is provided, please describe the nature of entity for 'n' in the legends of figures EV1 F
- Please note that the error bars are not defined in the legends of figures 2A, EV1 F

SOURCE DATA
- Upload each figure folder separately as individual zip folders: Figure 1 Source Data, Figure 2 Source Data, etc.
- Upload the completed source data checklist as a Related Manuscript File

SYNOPSIS
- Please provide a short (1-2 sentences) summary of the findings and their significance and 2-3 bullet points highlighting key results. You can upload this text as a Related Manuscript File. You are encouraged to look on recently published papers in EMBO Reports to get a better idea of the style used for the synopsis.
- Provide a schematic summary figure that provides a sketch of the major findings (not a data image). Provide this as a separate file in PNG/JPG/TIF/EPS (but not PDF!) and make sure it is exactly 550x300 pixels (width x height).

FIGURE ISSUE
- Figure EV4 Blots are over exposed. Please provide the source data for this figure (uploaded as Figure EV4 Source Data)

OTHER ISSUES
- Xionglei He needs ORCID in the account. We have sent the author another request to do so. Please note that all corresponding authors need to have their ORCID linked in our system. Please note that we cannot do this on the author's behalf for security reasons.

- Complete the corresponding author's name, journal and manuscript number in the Author Checklist
- Remove the Reagents table from the manuscript file. We need this uploaded separately. Please use the attached Word template and upload it as the reagents table
- Remove Guangdong Basic and Applied Basic Research Foundation (2025A1515011510) from the Comments box in the system and provide it as a separate funder (the blank entry above).

The authors have addressed all minor editorial requests.

3rd Revision - Editorial Decision 22nd May 2026

Dr. Li Liu
Sun Yat-Sen University
School of Life Sciences
#135 Xingang Xi Road
Guangzhou, Guangdong 510275
China

Dear Dr. Liu,

I am very pleased to accept your manuscript for publication in the next available issue of EMBO reports. Thank you for your contribution to our journal.

Your manuscript will be processed for publication by EMBO Press. It will be copy edited and you will receive page proofs prior to publication. Please note that you will be contacted by Springer Nature Author Services to complete licensing and payment information.

You may qualify for financial assistance for your publication charges - either via a Springer Nature fully open access agreement or an EMBO initiative. Check your eligibility: https://link.springer.com/journal/44319/how-to-publish-with-us

Should you be planning a Press Release on your article, please notify the production team via this form in order to coordinate publication and release dates: https://forms.cloud.microsoft/e/eH4A8JVP6g

If you have any questions, please do not hesitate to contact the Editorial Office. Thank you for your contribution to EMBO Reports.

Yours sincerely,

Yehu Moran
Academic Editor
EMBO Reports

>>> Please note that it is EMBO Reports policy for the transcript of the editorial process (containing referee reports and your response letter) to be published as an online supplement to each paper. If you do NOT want this, you will need to inform the Editorial Office via email immediately. More information is available here: https://link.springer.com/partners/embo-press/editorial-policies#Peer%20review

EMBO Press Author Checklist

Corresponding Author Name: Li Liu, Xionglei He	
Journal Submitted to: EMBO Reports	
Manuscript Number: EMBOR-2025-62721V3	

USEFUL LINKS FOR COMPLETING THIS FORM
The EMBO Journal - Author Guidelines
EMBO Reports - Author Guidelines
Molecular Systems Biology - Author Guidelines
EMBO Molecular Medicine - Author Guidelines

Reporting Checklist for Life Science Articles (updated January
This checklist is adapted from Materials Design Analysis Reporting (MDAR) Checklist for Authors. MDAR establishes a minimum set of requirements in transparent reporting in the life sciences (see Statement of Task: 10.31222/osf.io/9sm4x). Please follow the journal's guidelines in preparing your
Please note that a copy of this checklist will be published alongside your article.

Abridged guidelines for figures
1. Data
The data shown in figures should satisfy the following conditions:
- the data were obtained and processed according to the field's best practice and are presented to reflect the results of the experiments in an accurate and unbiased manner.
- ideally, figure panels should include only measurements that are directly comparable to each other and obtained with the same assay.
- plots include clearly labeled error bars for independent experiments and sample sizes. Unless justified, error bars should not be shown for technical
- if n<5, the individual data points from each experiment should be plotted. Any statistical test employed should be justified.
- Source Data should be included to report the data underlying figures according to the guidelines set out in the authorship guidelines on Data

2. Captions
Each figure caption should contain the following information, for each panel where they are relevant:
- a specification of the experimental system investigated (eg cell line, species name).
- the assay(s) and method(s) used to carry out the reported observations and measurements.
- an explicit mention of the biological and chemical entity(ies) that are being measured.
- an explicit mention of the biological and chemical entity(ies) that are altered/varied/perturbed in a controlled manner.
- the exact sample size (n) for each experimental group/condition, given as a number, not a range;
- a description of the sample collection allowing the reader to understand whether the samples represent technical or biological replicates (including how many animals, litters, cultures, etc.).
- a statement of how many times the experiment shown was independently replicated in the laboratory.
- definitions of statistical methods and measures:
  - common tests, such as t-test (please specify whether paired vs. unpaired), simple χ2 tests, Wilcoxon and Mann-Whitney tests, can be unambiguously identified by name only, but more complex techniques should be described in the methods section;
  - are tests one-sided or two-sided?
  - are there adjustments for multiple comparisons?
  - exact statistical test results, e.g., P values = x but not P values < x;
  - definition of 'center values' as median or average;
  - definition of error bars as s.d. or s.e.m.

Please complete ALL of the questions below.
Select "Not Applicable" only when the requested information is not relevant for your study.

Materials

Newly Created Materials	Information included in the manuscript?	In which section is the information available? (Reagents and Tools Table, Materials and Methods, Figures, Data Availability Section)
New materials and reagents need to be available; do any restrictions apply?	Not Applicable	

Antibodies	Information included in the manuscript?	In which section is the information available? (Reagents and Tools Table, Materials and Methods, Figures, Data Availability Section)
For **antibodies** provide the following information:<br>- Commercial antibodies: RRID (if possible) or supplier name, catalogue number and or/clone number<br>- Non-commercial: RRID or citation	Not Applicable	

DNA and RNA sequences	Information included in the manuscript?	In which section is the information available? (Reagents and Tools Table, Materials and Methods, Figures, Data Availability Section)
**Short novel DNA or RNA including primers, probes**: provide the sequences.	Yes	Dataset EV9

Cell materials	Information included in the manuscript?	In which section is the information available? (Reagents and Tools Table, Materials and Methods, Figures, Data Availability Section)
**Cell lines:** Provide species information, strain. Provide accession number in repository **OR** supplier name, catalog number, clone number, and/**OR** RRID.	Not Applicable	
**Primary cultures:** Provide species, strain, sex of origin, genetic modification status.	Not Applicable	
Report if the cell lines were recently **authenticated** (e.g., by STR profiling) and tested for mycoplasma contamination.	Not Applicable	

Experimental animals	Information included in the manuscript?	In which section is the information available? (Reagents and Tools Table, Materials and Methods, Figures, Data Availability Section)
**Laboratory animals or Model organisms:** Provide species, strain, sex, age, genetic modification status. Provide accession number in repository **OR** supplier name, catalog number, clone number, **OR** RRID.	Not Applicable	
**Animal observed in or captured from the field:** Provide species, sex, and age where possible.	Not Applicable	
Please detail **housing and husbandry conditions**.	Not Applicable	

Plants and microbes	Information included in the manuscript?	In which section is the information available? (Reagents and Tools Table, Materials and Methods, Figures, Data Availability Section)
**Plants:** provide species and strain, ecotype and cultivar where relevant, unique accession number if available, and source (including location for collected wild specimens).	Not Applicable	
**Microbes:** provide species and strain, unique accession number if available, and source.	Yes	Methods

Human research participants	Information included in the manuscript?	In which section is the information available? (Reagents and Tools Table, Materials and Methods, Figures, Data Availability Section)
If collected and within the bounds of privacy constraints report on age, sex and gender or ethnicity for all study participants.	Not Applicable	

Core facilities	Information included in the manuscript?	In which section is the information available? (Reagents and Tools Table, Materials and Methods, Figures, Data Availability Section)
If your work benefited from core facilities, was their service mentioned in the acknowledgments section?	Not Applicable	

Design

Study protocol	Information included in the manuscript?	In which section is the information available? (Reagents and Tools Table, Materials and Methods, Figures, Data Availability Section)
If study protocol has been **pre-registered, provide DOI in the manuscript**. For clinical trials, provide the trial registration number **OR** cite DOI.	Not Applicable	
Report the **clinical trial registration number** (at ClinicalTrials.gov or equivalent), where applicable.	Not Applicable	

Laboratory protocol	Information included in the manuscript?	In which section is the information available? (Reagents and Tools Table, Materials and Methods, Figures, Data Availability Section)
Provide DOI OR other citation details if **external detailed step-by-step protocols** are available.	Not Applicable	

Experimental study design and statistics	Information included in the manuscript?	In which section is the information available? (Reagents and Tools Table, Materials and Methods, Figures, Data Availability Section)
Include a statement about **sample size** estimate even if no statistical methods were used.	Yes	Methods
Were any steps taken to minimize the effects of subjective bias when allocating animals/samples to treatment (e.g. **randomization procedure**)? If yes, have they been described?	Not Applicable	
Include a statement about **blinding** even if no blinding was done.	Not Applicable	
Describe **inclusion/exclusion criteria** if samples or animals were excluded from the analysis. Were the criteria pre-established?<br><br>If sample or data points were omitted from analysis, report if this was due to attrition or intentional exclusion and provide justification.	Not Applicable	
For every figure, are **statistical tests** justified as appropriate? Do the data meet the assumptions of the tests (e.g., normal distribution)? Describe any methods used to assess it. Is there an estimate of variation within each group of data? Is the variance similar between the groups that are being statistically compared?	Yes	Figures

Sample definition and in-laboratory replication	Information included in the manuscript?	In which section is the information available? (Reagents and Tools Table, Materials and Methods, Figures, Data Availability Section)
In the figure legends: state number of times the experiment was **replicated** in laboratory.	Yes	Figures
In the figure legends: define whether data describe **technical or biological replicates**.	Yes	Figures

Ethics

Ethics	Information included in the manuscript?	In which section is the information available? (Reagents and Tools Table, Materials and Methods, Figures, Data Availability Section)
Studies involving **human participants**: State details of **authority granting ethics approval** (IRB or equivalent committee(s), provide reference number for approval.	Not Applicable	
Studies involving **human participants**: Include a statement confirming that **informed consent** was obtained from all subjects and that the experiments conformed to the principles set out in the WMA Declaration of Helsinki and the Department of Health and Human Services Belmont Report.	Not Applicable	
Studies involving **human participants:** For publication of **patient photos**, include a statement confirming that consent to publish was obtained.	Not Applicable	
Studies involving experimental **animals**: State details of **authority granting ethics approval** (IRB or equivalent committee(s), provide reference number for approval. Include a statement of compliance with ethical regulations.	Not Applicable	
Studies involving **specimen and field samples:** State if relevant **permits** obtained, provide details of authority approving study; if none were required, explain why.	Not Applicable	

Dual Use Research of Concern (DURC)	Information included in the manuscript?	In which section is the information available? (Reagents and Tools Table, Materials and Methods, Figures, Data Availability Section)
Could your study fall under dual use research restrictions? Please check biosecurity documents and list of **select agents and toxins** (CDC): https://www.selectagents.gov/sat/list.htm	Not Applicable	
If you used a select agent, is the security level of the lab appropriate and reported in the manuscript?	Not Applicable	
If a study is subject to dual use research of concern regulations, is the name of the **authority granting approval and reference number** for the regulatory approval provided in the manuscript?	Not Applicable	

Reporting

The MDAR framework recommends adoption of discipline-specific guidelines, established and endorsed through community initiatives. Journals have their own policy about requiring specific guidelines and recommendations to complement MDAR.

Adherence to community standards	Information included in the manuscript?	In which section is the information available? (Reagents and Tools Table, Materials and Methods, Figures, Data Availability Section)
State if relevant guidelines or checklists (e.g., **ICMJE, MIBBI, ARRIVE, PRISMA**) have been followed or provided.	Not Applicable	
For **tumor marker prognostic studies,** we recommend that you follow the **REMARK** reporting guidelines (see link list at top right). See author guidelines, under 'Reporting Guidelines'. Please confirm you have followed these guidelines.	Not Applicable	
For **phase II and III randomized controlled trials**, please refer to the **CONSORT** flow diagram (see link list at top right) and submit the CONSORT checklist (see link list at top right) with your submission. See author guidelines, under 'Reporting Guidelines'. Please confirm you have submitted this list.	Not Applicable	

Data Availability

Data availability	Information included in the manuscript?	In which section is the information available? (Reagents and Tools Table, Materials and Methods, Figures, Data Availability Section)
Have **primary datasets** been deposited according to the journal's guidelines (see 'Data Deposition' section) and the respective accession numbers provided in the Data Availability Section?	Yes	
Were **human clinical and genomic datasets** deposited in a public access-controlled repository in accordance to ethical obligations to the patients and to the applicable consent agreement?	Not Applicable	
Are **computational models** that are central and integral to a study available without restrictions in a machine-readable form? Were the relevant accession numbers or links provided?	Not Applicable	
If publicly available data were reused, provide the respective **data citations in the reference list**.	Yes	
